# De-motif sampling: an approach to decompose hierarchical motifs with applications in T cell recognition

**DOI:** 10.1093/bib/bbaf221

**Published:** 2025-05-16

**Authors:** Xinyi Tang, Ran Liu

**Affiliations:** Department of Mathematics, Statistics and Insurance, The Hang Seng University of Hong Kong, Hang Shin Link, Siu Lek Yuen, Shatin, N.T., Hong Kong SAR, China; Department of Statistics, The Chinese University of Hong Kong, Shatin, N.T., Hong Kong SAR, China; Department of Statistics, Faculty of Arts and Sciences, Beijing Normal University, No. 18 Jinfeng Road, Xiangzhou District, Zhuhai, Guangdong, 519087, China

**Keywords:** hierarchical motif, motif discovery, T cell recognition, epitope prediction

## Abstract

T cell immune recognition requires the interactions among antigen peptides, Major Histocompatibility Complex (MHC) molecules, and T cell receptors (TCRs). While research into the interactions between MHC and peptides is well established, the specific preferences of TCRs for peptides remain less understood. This gap largely stems from the requirement that antigen peptides must be bound to MHC and presented on the cell surface prior to recognition by TCRs. Typically, motifs related to TCR recognition are influenced by MHC characteristics, limiting the direct identification of TCR-specific motifs. To address this challenge, this study introduces a Bayesian method designed to decompose hierarchical motifs independently of MHC constraints. This model, rigorously tested through comprehensive simulation experiments and applied to real data, establishes a clear hierarchical structure for motifs related to T cell recognition.

## Introduction

Sequence motifs in biological contexts are defined as short, recurrent patterns within DNA, RNA, or protein sequences that are believed to play functional roles. These motifs are crucial for molecular binding interactions, such as protein-DNA binding, which influences transcriptional activity, and the interactions between RNA molecules and proteins, affecting RNA stability and translation. The identification and analysis of these motifs are essential for constructing detailed models of cellular mechanisms at the molecular level. Such insights are vital for advancing both fundamental biological research and applied biomedical sciences [1].

A key application of motif analysis lies in elucidating the interactions among Major Histocompatibility Complex (MHC) molecules, peptides, and T cell receptors (TCRs), which are crucial for immune recognition processes [2, 3]. MHC molecules bind to peptide fragments derived from pathogens and display these peptides on the cell surface. TCRs then recognize and bind to these MHC-presented peptides, triggering an immune response [4, 5]. Both MHC molecules and TCRs have specific preferences for binding certain peptide types, typically dictated by distinct motifs within the peptide sequences. These motifs are essential for the formation of stable complexes that are necessary for effective T-cell activation. Understanding these molecular interactions through motif analysis not only enhances our comprehension of immune recognition but also aids in the design of vaccines and immunotherapies by predicting peptide binding affinities and T-cell responses.

Due to the availability of extensive MHC binding data, common motif discovery algorithms are frequently applied to identify motifs within peptide sequences that bind to MHC molecules. In contrast, the landscape of binding motifs for peptide sequences that interact with TCRs remains largely uncharted. This gap primarily arises because TCR recognition is contingent upon MHC presentation; peptides must first bind to MHC molecules before they can interact with TCRs. This prerequisite complicates the direct identification of TCR-specific motifs, necessitating analysis of the MHC-peptide complexes involved. Currently, no computational algorithms have successfully identified TCR recognition motifs without considering MHC constraints. Advancing our understanding of TCR recognition motifs independently of MHC interactions could significantly enhance our knowledge of the amino acid (AA) preferences of TCRs and their specific binding mechanisms. Such insights are crucial for elucidating the selective interactions between TCRs and peptides, potentially leading to the development of more targeted immunotherapies and vaccines.

Hierarchical motifs refer to multiple motifs that arise from sequential biological processes. In such cases, a peptide that participates in a later step (e.g. T cell response) must have passed through all previous steps (e.g. MHC binding and presentation), and thus its sequence must satisfy the motif requirements of those earlier stages. Therefore, the motif observed in such a peptide is not purely determined by the final step alone, but rather reflects the cumulative influence of all prior motif constraints. This makes it difficult to study the motif associated with a single downstream process in isolation, as it is inherently shaped by the filtering imposed by upstream processes. In this study, we define five motifs with a hierarchical structure related to T cell recognition, based on their distinct functional roles:

- T Cell Response Motif: this motif encompasses the whole progression of events required for triggering a T cell response. It includes the binding of the peptide sequence to MHC, its presentation on the cell surface, and the subsequent T cell activation.
- TCR Recognition Motif: this motif represents the specific pattern within peptide sequences that are recognized by TCRs. It is characterized by a unique focus on the peptide–TCR interaction, independent of MHC restriction. It is important to note that this motif might not exist in natural peptide sequences as all peptides are presented to TCRs post-MHC presentation.
- MHC B&P (Binding and Presentation) Motif: this motif describes the patterns within peptide sequences that are bound by MHC and subsequently presented on the cell surface.
- MHC Binding Motif: involves specific sequences within peptides that exhibit a high affinity for MHC molecules.
- MHC Presentation Motif: defined to reflect the sequences that are preferred for MHC presentation.

**Figure 1:**
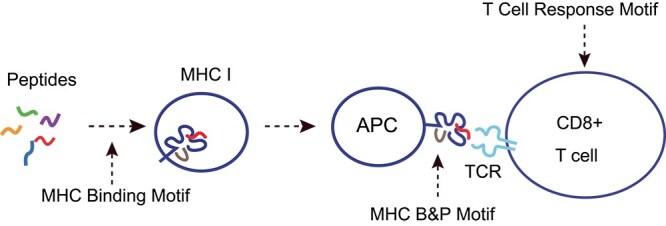
**T cell immune recognition process.**An antigenic peptide must bind to MHC and be presented on the surface of antigen-presenting cells before a TCR can recognize it.

Figure 1illustrates the immune recognition process and associated motifs. The MHC Presentation Motif is not explicitly shown in the figure because a peptide must first bind to MHC before it can be presented. As a result, only the MHC Binding Motif and MHC B&P Motif appear in the biological process. Similarly, the TCR Recognition Motif remains hidden, as a peptide must first bind to and be presented by MHC before it can be recognized by a TCR. We consider T Cell Response Motif as a composite of the TCR Recognition Motif and the MHC B&P Motif. The MHC B&P Motif itself is composed of both the MHC Binding Motif and the MHC Presentation Motif. Our goal is to analyze these motifs within their hierarchical structure and decompose them to uncover hidden motifs, such as the TCR Recognition Motif, independent of MHC constraints.

The field of motif discovery has made substantial progress due to the development of diverse statistical and computational techniques. Traditionally, these approaches have assumed that DNA bases or AAs at each binding position in a sequence conform to a categorical distribution—with K=4 for DNA and K=20 for AAs. Furthermore, these techniques often presuppose statistical independence among the positions within the motif. As a result, a position-specific probability matrix (PSPM) [6] is commonly utilized to represent the binding motifs. This matrix features columns that correspond to the parameters of the categorical distribution for each specific position in the motif. Simultaneously, DNA bases or AAs at nonbinding positions are considered independent samples from a background distribution.

The MEME Suite [1] is a comprehensive collection of tools for motif analysis, widely recognized in the bioinformatics community. Among its primary tools is MEME (Multiple EM for Motif Elicitation) [7], which utilizes an expectation maximization (EM) algorithm to infer motifs, treating binding positions as latent variables. For the discovery of short, ungapped motifs, the suite has introduced STREME (Discriminative Regular Expression Motif Elicitation) [8], which employs a suffix tree approach. STREME is noted for its speed and efficiency, particularly in handling large datasets. For motifs that include gaps, GLAM2 (Gapped Local Alignment of Motifs) [9] offers a strategy based on local alignments. Additional tools within the suite, such as MAST (Motif Alignment and Search Tool) [10] and FIMO (Find Individual Motif Occurrences) [11], are instrumental in searching for sequences containing identified motifs and assessing their significance. MAST focuses on the potential biological relevance of these motifs, while FIMO is dedicated to locating these motifs within larger sequences for detailed functional analysis.

Beyond the EM algorithm, Gibbs sampling is another prevalent method [12, 13]. This approach treats both latent variables and parameters as random variables, incorporating prior distributions into the analysis. A variant known as collapsed Gibbs sampling simplifies the process by integrating out certain parameters, thus avoiding direct sampling, and has been effectively applied in gene regulation studies [14]. Tools like Align ACE [15] employ an iterative masking strategy to identify multiple distinct motifs, while BioProspector [16] enhances analytical flexibility by relaxing positional constraints and using higher order Markov models to accommodate the biological intricacies of DNA sequences, particularly where each codon consists of three bases. Detailed reviews of these methodologies can be found in prior publications [17, 18].

However, a limitation of these methods is their inadequacy for analyzing hierarchical motifs, where dependencies exist between different levels of motifs.

In this study, we introduced a novel Bayesian approach specifically designed to decompose a composite hierarchical motif into two distinct motifs. Through a series of simulation studies, we evaluated the performance of our model under a range of scenarios. The results demonstrated that the parameter estimations closely approximated the actual values, indicating a high level of accuracy in our model’s predictive capabilities. Further, we applied our Bayesian model to problems involving T cell recognition, shedding new light on the motifs associated with this critical immune process. The insights gained from our analysis have potential implications for the design of a prediction pipeline for T cell epitopes.

## Methods

Although the model is inspired by hierarchical motifs related to T cell recognition, it can also be used for other biological bindings with hierarchical motifs. Therefore, the model is described in a general way for all biological sequences.

### Model

Assume that we have $n$biological sequences, represented by $\boldsymbol{R}=\left (\boldsymbol{r}_{1}, \boldsymbol{r}_{2}, \ldots , \boldsymbol{r}_{n}\right )$, in which two types of binding events or selective biological processes occur. The vectors $\boldsymbol{W} = (w_{1}, w_{2}, \cdots , w_{n})$and $\boldsymbol{G} = (g_{1}, g_{2}, \cdots , g_{n})$correspond to the presence of these two bindings in each sequence. For instance, the binary $w_{i}$(either 1 or 0) indicates whether the first type of binding is present (1) or absent (0) in the $i$th sequence $\boldsymbol{r}_{i}$. Similarly, $g_{i}$indicates the presence (1) or absence (0) of the second binding in the $i$th sequence $\boldsymbol{r}_{i}$. We denote $\boldsymbol{W}_{U} = \{w_{u_{1}}, w_{u_{2}},\cdots , w_{u_{l}}\}$and $\boldsymbol{W}_{U^{c}} = \boldsymbol{W}-\boldsymbol{W}_{U}$as the unknown and known label vectors, respectively, for the first binding. Here the minus operator for two sets means set difference. Similarly, $\boldsymbol{G}_{\widetilde{U}} = \{g_{\widetilde{u}_{1}}, g_{\widetilde{u}_{2}},\cdots , g_{\widetilde{u}_{\widetilde{l}}}\}$and $\boldsymbol{G}_{\widetilde{U}^{c}} = \boldsymbol{G}-\boldsymbol{G}_{\widetilde{U}}$are designated as the unknown and known label vectors for the second binding, respectively. The number of unknown $\boldsymbol{W}$is $l$and the number of unknown $\boldsymbol{G}$is $\widetilde{l}$.

Consider $\boldsymbol{A}=\left [a_{i j}\right ]_{1 \leq i \leq n, 1 \leq j \leq J}$and $\boldsymbol{B}=\left [b_{i j}\right ]_{1 \leq i \leq n, 1 \leq j \leq \widetilde{J}}$as the position matrices for binding sites, with known motif lengths $J$and $\widetilde{J}$, symbolizing the first and second bindings, respectively. In this context, $a_{i,j}$serves as the index that represents the $j$th binding sites of the first binding process on the $i$th sequence. $b_{i,j}$serves as the index that represents the $j$th binding sites of the second binding process on the $i$th sequence. We assume the binding positions for a single sequence are continuous. Once $a_{i1}$(or $b_{i1}$) is established, it automatically determines $\boldsymbol{a}_{i}$(or $\boldsymbol{b}_{i}$). We assume that letters at the binding locations stem from one of two distinct product categorical distributions. If a position coincides with both bindings, we assume that the letter at this position is sampled from the second product categorical distribution. The position probability matrices for the first and second bindings are denoted as $[\boldsymbol{\Theta }]_{K \times J}$and $[\widetilde{\boldsymbol{\Theta }}]_{K \times \widetilde{J}}$, respectively. The $j$th columns ${\boldsymbol{\Theta }}_{j}$and $\widetilde{\boldsymbol{\Theta }}_{j}$represent the probability parameters of the multinomial distribution for the $j$th positions of the first and second bindings. The number of rows, $K$, is equal to the count of letter types—4 for DNA sequences and 20 for AA sequences. For all letters not situated at a binding position, we assume that they come from another categorical distribution with background probability $\boldsymbol{\theta }_{0}$.

**Figure 2:**
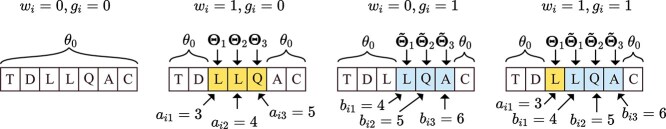
**A toy example showing the AAs with the corresponding parameters under all four cases.**In the first case, no binding occurs, and all residues follow the background distribution. In the second case, the first motif binds at positions 3, 4, and 5 (highlighted in yellow), and these residues follow the motif distribution, while the remaining residues follow the background. In the third case, the second motif binds at positions 4, 5, and 6 (highlighted in blue), which follow a different motif distribution, and the rest follow the background. In the fourth case, both motifs bind; the overlapping positions (4 and 5) are assigned to the second motif distribution, the non-overlapping position (3) follows the first motif distribution, and all other residues follow the background.

There are four binding cases for a sequence $ r_{i} $:

1. 
$ w_{i}=0, g_{i}=0 $ : this label represents that no binding occurs; all residues (AAs or DNA bases) in the sequence are drawn from the background distribution $ \boldsymbol{\theta _{0}} $. The likelihood is given by $ \boldsymbol{\theta }_{0}^{h\left (r_{i}\right )} $.
2. 
$ w_{i}=1, g_{i}=0 $ : this label represents that the first binding occurs, while the second binding does not. The residues at the first binding positions $ \boldsymbol{a}_{i} $are drawn from the first motif distribution $ \boldsymbol{\Theta } $, with each binding residue coming from one of the columns $ \boldsymbol{\Theta }_{j} $. The remaining residues follow the background distribution. The likelihood is given by
  \begin{align*} & \boldsymbol{\theta}_{0}^{h\left(r_{i},\{\boldsymbol{a}_{i}^{c}\}\right)}\prod_{j=1}^{J}\boldsymbol{\Theta}_{j}^{h(r_{i,a_{ij}})}. \end{align*}
3. 
$ w_{i}=0, g_{i}=1 $ : this label represents that the second binding occurs, while the first binding does not. Similarly, the residues at the second binding positions $ \boldsymbol{b}_{i} $are drawn from the second motif distribution $ \widetilde{\boldsymbol{\Theta }} $, while the remaining residues follow the background distribution. The likelihood is given by
  \begin{align*} & \boldsymbol{\theta}_{0}^{h\left(r_{i},\{\boldsymbol{b}_{i}^{c}\}\right)}\prod_{j=1}^{\tilde{J}}\widetilde{\boldsymbol{\Theta}}_{j}^{h(r_{i,b_{ij}})}. \end{align*}
4. 
$ w_{i}=1, g_{i}=1 $ : this label represents that both bindings occur. In this case, $ \boldsymbol{a}_{i} $and $ \boldsymbol{b}_{i} $may overlap. Due to the hierarchy, we assume that residues at the overlapping positions are drawn from the second motif distribution, while residues at the nonoverlapping positions of $ \boldsymbol{a}_{i} $are drawn from the first motif distribution. The remaining residues follow the background distribution. The likelihood is given by
  \begin{align*} & \boldsymbol{\theta}_{0}^{h\left(r_{i},\{\boldsymbol{a}_{i}\cup \boldsymbol{b}_{i}\}^{c}\right)}\prod_{j=1}^{\tilde{J}}\widetilde{\boldsymbol{\Theta}}_{j}^{h(r_{i,b_{ij}})}\prod_{\{j:a_{ij}\notin \boldsymbol{b}_{i}, 1\leq j\leq J\}}\boldsymbol{\Theta}_{j}^{h(r_{i,a_{ij}})}. \end{align*}
  A toy example is illustrated in Fig. 2. Suppose we have an AA sequence $ r_{i} $, “TDLLQAC.” The lengths of both the first and second motifs are 3, with $ \boldsymbol{a}_{i}=\{3,4,5\} $and $ \boldsymbol{b}_{i}=\{3,4,5\} $. Figure 2shows the AAs with the corresponding parameters under all four cases.

We have the observed data likelihood for all sequences:


\begin{gather*} \mathbf{P}\left(\boldsymbol{R},\boldsymbol{W}_{U^{c}},\boldsymbol{G}_{\widetilde{U}^{c}}\mid \boldsymbol{W}_{U}, \boldsymbol{G}_{\widetilde{U}},\boldsymbol{A},\boldsymbol{B}, \boldsymbol{\Theta},\widetilde{\boldsymbol{\Theta}},\boldsymbol{\theta}_{0}\right) \\ \propto\prod_{i=1}^{n}\left[\boldsymbol{\theta}_{0}^{h\left(r_{i},\{\boldsymbol{a}_{i}\cup \boldsymbol{b}_{i}\}^{c}\right)}\prod_{j=1}^{\tilde{J}}\widetilde{\boldsymbol{\Theta} }_{j}^{h(r_{i,b_{ij}})}\prod_{\{j:a_{ij}\notin \boldsymbol{b}_{i}, 1\leq j\leq J\}}\boldsymbol{\Theta}_{j}^{h(r_{i,a_{ij}})}\right]^{I(w_{i}=1,g_{i}=1)}\\ \times\left[\boldsymbol{\theta}_{0}^{h\left(r_{i},\{\boldsymbol{a}_{i}^{c}\}\right)}\prod_{j=1}^{J}\boldsymbol{\Theta}_{j}^{h(r_{i,a_{ij}})}\right]^{I(w_{i}=1,g_{i}=0)}\times\left[\boldsymbol{\theta}_{0}^{h\left(r_{i}\right)}\right]^{I(w_{i}=0,g_{i}=0)}\\ \times\left[\boldsymbol{\theta}_{0}^{h\left(r_{i},\{\boldsymbol{b}_{i}^{c}\}\right)}\prod_{j=1}^{\tilde{J}}\widetilde{\boldsymbol{\Theta}}_{j}^{h(r_{i,b_{ij}})}\right]^{I(w_{i}=0,g_{i}=1)}. \end{gather*}

The term $\boldsymbol{\Theta }_{j}^{h(r_{i, a_{i j}})}$denotes the likelihood of the letter $r_{i, a_{i j}}$occurring at the $j$th binding site of the $i$th sequence. Suppose we have $k$kinds of letters $\{O_{1},O_{2},\cdots , O_{K}\}$; we can express $\boldsymbol{\Theta }_{j}^{h(r_{i,a_{ij}})}$as $\prod _{k=1}^{K}\boldsymbol{\Theta }_{kj}^{I(r_{i,a_{ij}}=O_{k})}$. $\boldsymbol{a}_{i}$refers to the $i$th row of the binding location matrix $\boldsymbol{A}$, and ${\boldsymbol{a}_{i}^{c}}$is the location vector of $\boldsymbol{r}_{i}$excluding the binding site $\boldsymbol{a}_{i}$. Similarly, we can define $\boldsymbol{b}_{i}$and ${\boldsymbol{b}_{i}^{c}}$.

### Bayesian inference

We employed Markov Chain Monte Carlo (MCMC) methods to conduct Bayesian inference, leveraging a Markov chain to sample from the posterior distribution. The process begins with an initial distribution and gradually converges to the stationary distribution, which corresponds to the desired posterior distribution. In this study, we utilized two specific MCMC algorithms—Gibbs sampling and the Metropolis-Hastings algorithm—to sample the model parameters and latent variables. Conjugate priors were assigned to the unknown parameters:


\begin{gather*} \boldsymbol{\theta}_{0} \sim Dirichlet\left(\boldsymbol{\alpha}_{0}\right),\\ \boldsymbol{\Theta}_{j} \sim Dirichlet\left(\boldsymbol{\alpha}_{j}\right),\,1\leq j\leq J \,,\\ \widetilde{\boldsymbol{\Theta}}_{j} \sim Dirichlet\left(\widetilde{\boldsymbol{\alpha}}_{j}\right),\,1\leq j\leq \widetilde{J}. \end{gather*}

Additionally, conjugate priors were assigned to the following latent variables:


\begin{gather*} a_{i1} \sim Cat(L_{i}-J+1,\boldsymbol{\pi}_{0,a_{i}}), \quad 1\leq i\leq n,\\ b_{i1} \sim Cat(L_{i}-\widetilde{J}+1,\boldsymbol{\pi}_{0,b_{i}}), \quad 1\leq i\leq n,\\ w_{u_{i}}\sim Bernoulli\left(p_{0}\right),\,1\leq i\leq l,\\ g_{\widetilde{u}_{i}}\sim Bernoulli\left(p_{0}\right),\,1\leq i\leq \tilde{l}, \end{gather*}

where $L_{i}$is the length of the $i$th sequence. Next, we aim to obtain the full conditional distributions for each parameter. For the parameters $\boldsymbol{\Theta }_{j}$, $\widetilde{\boldsymbol{\Theta }}_{j}$, and $\boldsymbol{\theta _{0}}$, the full conditional posterior distributions are as follows:


\begin{gather*} \boldsymbol{\Theta}_{j}\,|\,- \sim Dirichlet\left(\boldsymbol{H}_{\boldsymbol{A}_{j}}+\boldsymbol{\alpha}_{j}\right),\,1<j<J,\\ \widetilde{\boldsymbol{\Theta}}_{j}\,|\,- \sim Dirichlet\left(\boldsymbol{H}_{\boldsymbol{B}_{j}}+\widetilde{\boldsymbol{\alpha}}_{j}\right),\,1<j<\widetilde{J},\\ \boldsymbol{\Theta}_{0}\,|\,- \sim Dirichlet\left(\boldsymbol{H}_{0}+\boldsymbol{\alpha}_{0}\right), \end{gather*}

where


\begin{gather*} \boldsymbol{H}_{\boldsymbol{A}_{j}}=\sum_{i=1}^{n} h(r_{i,a_{ij}})\cdot I(w_{i}=1,g_{i}=1)I(a_{ij}\notin \boldsymbol{b}_{i})\\ +\sum_{i=1}^{n} h(r_{i,a_{ij}})\cdot I(w_{i}=1,g_{i}=0),\\ \boldsymbol{H}_{\boldsymbol{B}_{j}}=\sum_{i=1}^{n} h(r_{i,b_{ij}})\cdot\left[I(w_{i}=1,g_{i}=1)+I(w_{i}=0,g_{i}=1)\right],\\ \boldsymbol{H}_{0}=\sum_{i=1}^{n} h(r_{i,\{\boldsymbol{a}_{i}\cup \boldsymbol{b}_{i}\}^{c}})I(w_{i}=1,g_{i}=1)+\sum_{i=1}^{n} h(r_{i})I(w_{i}=0,g_{i}=0)\\ +\sum_{i=1}^{n} h(r_{i,\boldsymbol{b}_{i}^{c}})I(w_{i}=0,g_{i}=1)+\sum_{i=1}^{n} h(r_{i,\boldsymbol{a}_{i}^{c}})I(w_{i}=1,g_{i}=0). \end{gather*}

The dashed line represents all other parameters, which include the binding label vectors $\boldsymbol{W}$and $\boldsymbol{G}$, the binding location matrices $\boldsymbol{A}$and $\boldsymbol{B}$, and the observed data $\boldsymbol{R}$.

Regarding the binding location label vectors $\boldsymbol{W}$and $\boldsymbol{G}$, we have the full conditional posterior distributions:


\begin{gather*} w_{u_{i}}\sim Bernoulli\left(p_{pos,w_{u_{i}}}\right),\,1\leq i\leq l,\\ g_{\widetilde{u}_{j}}\sim Bernoulli\left(p_{pos,g_{\tilde{u}_{j}}}\right),\,1\leq j\leq \tilde{l}, \end{gather*}

where


\begin{gather*} p_{pos,w_{u_{i}}}=\frac{f_{w}(w_{u_{i}}=1)}{f_{w}(w_{u_{i}}=1)+f_{w}(w_{u_{i}}=0)},\\ p_{pos,g_{\widetilde{u}_{j}}}=\frac{f_{g}(g_{\widetilde{u}_{j}}=1)}{f_{g}(g_{\widetilde{u}_{j}}=1)+f_{g}(g_{\widetilde{u}_{j}}=0)},\\ \end{gather*}

the “pos” subscript indicates posterior-related. And we have


\begin{align*} f_{w}(w_{u_{i}}=x) \triangleq& \mathbf{P}\left(r_{u_{i}},\boldsymbol{G}_{\widetilde{U}^{c}}\mid \boldsymbol{G}_{\widetilde{U}}, w_{u_{i}}=x,\boldsymbol{a}_{u_{i}},\boldsymbol{b}_{u_{i}}, \boldsymbol{\Theta},\widetilde{\boldsymbol{\Theta}},\boldsymbol{\theta}_{0}\right)\\ &\quad\quad\quad\times\mathbf{P}\left(w_{u_{i}}=x\right),\\ f_{g}(g_{\widetilde{u}_{j}}=x) \triangleq& \mathbf{P}\left(r_{u_{i}},\boldsymbol{W}_{U^{c}}\mid \boldsymbol{W}_{U}, g_{\widetilde{u}_{i}}=x,\boldsymbol{a}_{u_{i}},\boldsymbol{b}_{u_{i}}, \boldsymbol{\Theta},\widetilde{\boldsymbol{\Theta}},\boldsymbol{\theta}_{0}\right)\\ &\quad\quad\quad\times\mathbf{P}\left(g_{\widetilde{u}_{i}}=x\right). \end{align*}

For the binding location matrices $\boldsymbol{A}$and $\boldsymbol{B}$, each row vector is conditionally independent, allowing us to update each row vector in parallel:


\begin{gather*} a_{i1} \sim Cat(L_{i}-J+1,\boldsymbol{\pi}_{pos,a_{i}}),\quad 1\leq i\leq n, \\ b_{i1} \sim Cat(L_{i}-\widetilde{J}+1,\tilde{\boldsymbol{\pi}}_{pos,b_{i}}), \quad 1\leq i\leq n, \end{gather*}

with $\boldsymbol{\pi }_{pos,a_{i}} = \{\pi _{pos,a_{i},1},\pi _{pos,a_{i},2},\cdots ,\pi _{pos,a_{i},L_{i}-J+1}\}$and $\tilde{\boldsymbol{\pi }}_{pos,b_{i}} = \{\pi _{pos,b_{i},1},\pi _{pos,b_{i},2},\cdots ,\pi _{pos,b_{i},L_{i}-\widetilde{J}+1}\}$. Here, $\boldsymbol{\pi }_{pos,a_{i}}$and $\tilde{\boldsymbol{\pi }}_{pos,b_{i}}$are


\begin{gather*} \pi_{pos,a_{i},l_{a}}=\frac{f_{a}(a_{i1}=l_{a})}{\sum_{x_{a}=1}^{L_{i}-J+1}f_{a}(a_{i1}=x_{a})},\\ \quad\pi_{pos,b_{i},l_{b}}=\frac{f_{b}(b_{i1}=l_{b})}{\sum_{x_{b}=1}^{L_{i}-\widetilde{J}+1}f_{b}(b_{i1}=x_{b})}. \end{gather*}

Given the continuity of the motif, we have $\boldsymbol{x}_{a}=\{x_{a1},x_{a2},\cdots ,x_{aJ}\} = \{x_{a},x_{a}+1,\cdots ,x_{a}+J-1\}$and $\boldsymbol{x}_{b}=\{x_{b1},x_{b2},\cdots ,x_{b\widetilde{J}}\} = \{x_{b},x_{b}+1,\cdots ,x_{b}+\widetilde{J}-1\}$:


\begin{align*} f_{a}(a_{i1}=x_{a}) \triangleq& \left[\boldsymbol{\theta}_{0}^{h\left(r_{i},\{\boldsymbol{x}_{a}\cup \boldsymbol{b}_{i}\}^{c}\right)}\prod_{j=1}^{\tilde{J}}\widetilde{\boldsymbol{\Theta} }_{j}^{h(r_{i,b_{ij}})} \times \prod_{\{j:x_{aj}\notin \boldsymbol{b}_{i}, 1\leq j\leq J\}}\boldsymbol{\Theta}_{j}^{h(r_{i,x_{aj}})}\right]^{I(w_{i}=1,g_{i}=1)}\\ &\times\left[\boldsymbol{\theta}_{0}^{h\left(r_{i},\{\boldsymbol{x}_{a}^{c}\}\right)}\prod_{j=1}^{J}\boldsymbol{\Theta}_{j}^{h(r_{i,x_{aj}})}\right]^{I(w_{i}=1,g_{i}=0)},\\ f_{b}(b_{i1}=x_{b}) \triangleq& \left[\boldsymbol{\theta}_{0}^{h\left(r_{i},\{\boldsymbol{a}_{i}\cup \boldsymbol{x}_{b}\}^{c}\right)}\prod_{j=1}^{\tilde{J}}\widetilde{\boldsymbol{\Theta} }_{j}^{h(r_{i,x_{bj}})}\times\prod_{\{j:a_{ij}\notin \boldsymbol{x}_{b}, 1\leq j\leq J\}}\boldsymbol{\Theta}_{j}^{h(r_{i,a_{ij}})}\right]^{I(w_{i}=1,g_{i}=1)}\\ &\times\left[\boldsymbol{\theta}_{0}^{h\left(r_{i},\{\boldsymbol{x}_{b}^{c}\}\right)}\prod_{j=1}^{\tilde{J}}\widetilde{\boldsymbol{\Theta}}_{j}^{h(r_{i,x_{bj}})}\right]^{I(w_{i}=0,g_{i}=1)}. \end{align*}

### Metropolis-hastings steps

To enhance the efficiency of MCMC, which frequently encounters difficulties in escaping local modes in complex, high-dimensional distributions, we introduce two novel group variable shift strategies derived from the Metropolis-Hastings (MH) algorithm.

#### Shift move for $\boldsymbol{A}$, $\boldsymbol{\Theta }$and $\boldsymbol{W}_{U}$

As outlined in the Collapsed Gibbs algorithm [14], motif sampling algorithms are prone to getting stuck in a local mode. Let us define $\mathbf{A_{(1)}^{0}} = (a_{11}^{0}, a_{21}^{0},..., a_{n1}^{0})$as the starting positions of the actual first binding locations, and assume that it lies at the true mode of the distribution. Consequently, these locations $\mathbf{A_{(1)}} = \mathbf{A_{(1)}^{0}} + \delta = (a_{11}^{0}+ \delta , a_{21}^{0}+ \delta ,..., a_{n1}^{0}+ \delta )$, where $\delta $is a small integer, are also considered local modes of the distribution. They deviate from the true mode by a consistent shift.

Given that variables in this model are highly related, such as the locations, the motif matrix, and the binding label for the first binding process, acceptance becomes challenging if we only shift the locations. Therefore, in addition to the shifted binding locations, we also propose new values for other variables:

**Step 1**: propose the candidates $\boldsymbol{A}^{*}$, $\boldsymbol{\Theta }^{*}$, and $\boldsymbol{W}^{*}_{U}$:

(1). Propose the matrix $\boldsymbol{A}^{*}$as follows: $\boldsymbol{A}^{*} = \boldsymbol{A} + \delta \boldsymbol{I}$. Here, $\boldsymbol{I}$represents an $n \times J$matrix in which all elements are 1. The variable $\delta $can take on the values of $-1$or $1$, each with a probability of $1/2$. In this way, the proposal distribution $q\left (\boldsymbol{A}^{*} \mid - \right ) = 1/2$.
(2). For $j=1,\cdots J$, we propose $\boldsymbol{\Theta }_{j}^{*}$from a Dirichlet distribution with the parameter $\boldsymbol{H}_{\boldsymbol{A}^{*}_{j}}+\boldsymbol{\alpha }_{j}$, where $\boldsymbol{H}_{\boldsymbol{A}^{*}_{j}} + \boldsymbol{\alpha }_{j} = \sum _{i=1}^{n} h(r_{i,a^{*}_{ij}}) \big [ I(w_{i} = 1, g_{i} = 1) I(a^{*}_{ij} \notin \boldsymbol{b}_{i}) + I(w_{i} = 1, g_{i} = 0) \big ].$The proposal distribution is $q\left (\boldsymbol{\Theta }^{*} \mid \boldsymbol{A}^{*}, - \right ) = \prod _{j}q\left (\boldsymbol{\Theta }_{j}^{*} \mid \boldsymbol{A}_{j}^{*}, - \right )$in which $q\left (\boldsymbol{\Theta }_{j}^{*} \mid \boldsymbol{A}_{j}^{*}, - \right )$is the probability of $\boldsymbol{\Theta }_{j}^{*}$, which follows a Dirichlet distribution with parameter $\boldsymbol{H}_{\boldsymbol{A}^{*}_{j}}+\boldsymbol{\alpha }_{j}$.
(3). For $i$ranging from $1$to $l$, we propose a value $w^{*}_{u_{i}}$selected from the set $\{0, 1\}$with associated probabilities $f^{*}(w^{*}_{u_{i}}=0)$and $f^{*}(w^{*}_{u_{i}}=1)$. Here,
  \begin{align*} f^{*}(w^{*}_{u_{i}}=z) \propto &\mathbf{P}\left(\boldsymbol{r}_{u_{i}},\boldsymbol{G}_{\widetilde{U}^{c}}\mid w^{*}_{u_{i}}=z, \boldsymbol{G}_{\widetilde{U}}, \boldsymbol{\Theta}^{*},\widetilde{\boldsymbol{\Theta}},\boldsymbol{\theta}_{0},\boldsymbol{a}^{*}_{u_{i}},\boldsymbol{b}_{u_{i}}\right) \\ &\times\mathbf{P}\left( w^{*}_{u_{i}}=z\right) \\ \triangleq& f(w^{*}_{u_{i}}=z),\\ f^{*}(w^{*}_{u_{i}}=z) =&\frac{f(w^{*}_{u_{i}}=z)}{f(w^{*}_{u_{i}}=0)+f(w^{*}_{u_{i}}=1)}. \end{align*}
  The proposal distribution is $q\left (\boldsymbol{W}_{U}^{*} \mid \boldsymbol{A}^{*}, \boldsymbol{\Theta }^{*}, - \right ) = \prod _{i=1}^{l}f^{*}\\(w^{*}_{u_{i}}=z)$.

**Step 2**: acceptance or rejection:

(1). Calculate the acceptance rate ($\alpha $):
  \begin{align*} \alpha \triangleq \min&\left\{1,\frac{\pi\left(\boldsymbol{W}_{U}^{*}, \boldsymbol{A}^{*}, \boldsymbol{\Theta}^{*} \mid - \right)}{\pi\left(\boldsymbol{W}_{U}, \boldsymbol{A}, \boldsymbol{\Theta} \mid - \right)}\right.\\ &\left.\times \frac{q\left(\boldsymbol{A} \mid - \right)q\left(\boldsymbol{\Theta} \mid \boldsymbol{A}, - \right)q\left(\boldsymbol{W}_{U} \mid \boldsymbol{A}, \boldsymbol{\Theta}, - \right)}{q\left(\boldsymbol{A}^{*} \mid - \right)q\left(\boldsymbol{\Theta}^{*} \mid \boldsymbol{A}^{*}, - \right)q\left(\boldsymbol{W}_{U}^{*} \mid \boldsymbol{A}^{*}, \boldsymbol{\Theta}^{*}, - \right)}\right\}, \end{align*}
  where joint distribution $\pi $is
  \begin{align*} &\pi\left(\boldsymbol{W}_{U}^{*}, \boldsymbol{A}^{*}, \boldsymbol{\Theta}^{*} \mid - \right)\\ \propto &\mathbf{P}\left(\boldsymbol{R},\boldsymbol{W}_{U^{c}},\boldsymbol{G}_{\widetilde{U}^{c}}\mid \boldsymbol{W}_{U}^{*}, \boldsymbol{G}_{\widetilde{U}}, \boldsymbol{\Theta}^{*},\widetilde{\boldsymbol{\Theta}},\boldsymbol{\theta}_{0},\boldsymbol{A}^{*},\boldsymbol{B}\right)\\ &\times \mathbf{P}\left(\boldsymbol{W}_{U}^{*}\right)\cdot \mathbf{P}\left(\boldsymbol{A}^{*}\right)\cdot \mathbf{P}\left(\boldsymbol{\Theta}^{*}\right), \end{align*}
  $q\left (\boldsymbol{A}^{*} \mid - \right )$, $q\left (\boldsymbol{\Theta }^{*} \mid \boldsymbol{A}^{*}, - \right )$, and $q\left (\boldsymbol{W}_{U}^{*} \mid \boldsymbol{A}^{*}, \boldsymbol{\Theta }^{*}, - \right )$are probabilities calculated by the proposal distributions mentioned above.
(2). Generate a random number $s$from $Unif(0,1)$, if $s\leq \alpha $, then we accept $\boldsymbol{A}^{*}$, $\boldsymbol{\Theta }^{*}$, and $\boldsymbol{W}^{*}_{U}$as new parameter values, otherwise, reject them.

#### Shift move for $\boldsymbol{B}$, $\widetilde{\boldsymbol{\Theta }}$, and $\boldsymbol{G}_{\widetilde{U}}$

The MH step for $\boldsymbol{B}$, $\widetilde{\boldsymbol{\Theta }}$, and $\boldsymbol{G}_{\widetilde{U}}$is similar to that for $\boldsymbol{A}^{*}$, $\boldsymbol{\Theta }^{*}$, and $\boldsymbol{W}^{*}_{U}$, which is presented as follows:

**Step 1**: propose the candidates $\boldsymbol{B}^{*}$, $\widetilde{\boldsymbol{\Theta }}^{*}$, and $\boldsymbol{G}^{*}_{\widetilde{U}}$

(1). Propose matrix $\boldsymbol{B}^{*}$as follows: $\boldsymbol{B}^{*} = \boldsymbol{B} + \delta \boldsymbol{I}$. Here, $\boldsymbol{I}$represents an $n \times \tilde{J}$matrix in which all elements are 1. The variable $\delta $can take on the values of $-1$or $1$, each with a probability of $1/2$. In this way, the proposal distribution $q\left (\boldsymbol{B}^{*} \mid - \right ) = 1/2$.
(2). For $j=1,\cdots \tilde{J}$, we propose $\widetilde{\boldsymbol{\Theta }}_{j}^{*}$from a Dirichlet distribution with parameter $\boldsymbol{H}_{\boldsymbol{B}^{*}_{j}}+\widetilde{\boldsymbol{\alpha }}_{j}$, where $\boldsymbol{H}_{\boldsymbol{B}^{*}_{j}}=\sum _{i=1}^{n} h(r_{i,b^{*}_{ij}})\cdot \left [I(w_{i}=1,g_{i}=1)+I(w_{i}=0,g_{i}=1)\right ]$. Then, the proposal distribution is $q\left (\widetilde{\boldsymbol{\Theta }}^{*} \mid \boldsymbol{B}^{*}, - \right ) = \prod _{j}q\left (\widetilde{\boldsymbol{\Theta }}_{j}^{*} \mid \boldsymbol{B}_{j}^{*}, - \right )$in which $q\left (\widetilde{\boldsymbol{\Theta }}_{j}^{*} \mid \boldsymbol{B}_{j}^{*}, - \right )$is the probability of $\widetilde{\boldsymbol{\Theta }}_{j}^{*}$, which follows a Dirichlet distribution with parameter $\boldsymbol{H}_{\boldsymbol{B}^{*}_{j}}+\widetilde{\boldsymbol{\alpha }}_{j}$.
3). For $i$ranging from $1$to $\tilde{l}$, we propose a value $g^{*}_{\tilde{u}_{i}}$selected from the set $0$, $1$with associated probabilities $f^{*}(g^{*}_{\tilde{u}_{i}}=0)$and $f^{*}(g^{*}_{\tilde{u}_{i}}=1)$. Here,
  \begin{align*} f^{*}(g^{*}_{\tilde{u}_{i}}=z) \propto& \mathbf{P}\left(\boldsymbol{r}_{u_{i}},\boldsymbol{W}_{U^{c}}\mid g^{*}_{\tilde{u}_{i}}=z, \boldsymbol{W}_{U}, \boldsymbol{\Theta},\widetilde{\boldsymbol{\Theta}}^{*},\boldsymbol{\theta}_{0},\boldsymbol{a}_{u_{i}},\boldsymbol{b}^{*}_{u_{i}}\right)\\ &\times \mathbf{P}\left( g^{*}_{\tilde{u}_{i}}=z\right) \\ \triangleq& f(g^{*}_{\tilde{u}_{i}}=z),\\ f^{*}(g^{*}_{\tilde{u}_{i}}=z) =& \frac{f(g^{*}_{\tilde{u}_{i}}=z)}{f(g^{*}_{\tilde{u}_{i}}=0)+f(g^{*}_{\tilde{u}_{i}}=1)} \end{align*}
  Then, the proposal distribution is $q\left (\boldsymbol{G}_{\widetilde{U}}^{*} \mid \boldsymbol{B}^{*}, \boldsymbol{\Theta }^{*}, - \right ) = \prod _{i=1}^{l}f^{*}(g^{*}_{\tilde{u}_{i}}=z).$

**Step 2**: acceptance or rejection:

(1). Calculate the acceptance rate ($\alpha $):
  \begin{align*} \alpha \triangleq \min&\left\{1,\frac{\pi\left(\boldsymbol{G}_{\widetilde{U}}^{*}, \boldsymbol{B}^{*}, \widetilde{\boldsymbol{\Theta}}^{*} \mid - \right)}{\pi\left(\boldsymbol{G}_{\widetilde{U}}, \boldsymbol{B}, \widetilde{\boldsymbol{\Theta}} \mid - \right)}\right.\\ &\left.\quad\times \frac{q\left(\boldsymbol{B} \mid - \right)q\left(\widetilde{\boldsymbol{\Theta}} \mid \boldsymbol{B}, - \right)q\left(\boldsymbol{G}_{U} \mid \boldsymbol{B}, \widetilde{\boldsymbol{\Theta}}, - \right)}{q\left(\boldsymbol{B}^{*} \mid - \right)q\left(\widetilde{\boldsymbol{\Theta}}^{*} \mid \boldsymbol{B}^{*}, - \right)q\left(\boldsymbol{G}_{\widetilde{U}}^{*} \mid \boldsymbol{B}^{*}, \widetilde{\boldsymbol{\Theta}}^{*}, - \right)}\right\}, \end{align*}
  where joint distribution $\pi $is
  \begin{align*} &\pi\left(\boldsymbol{G}_{\widetilde{U}}^{*}, \boldsymbol{B}^{*}, \widetilde{\boldsymbol{\Theta}}^{*} \mid - \right)\\ \propto& \mathbf{P}\left(\boldsymbol{R},\boldsymbol{W}_{U^{c}},\boldsymbol{G}_{\widetilde{U}^{c}}\mid \boldsymbol{W}_{U}, \boldsymbol{G}_{\widetilde{U}}^{*}, \boldsymbol{\Theta},\widetilde{\boldsymbol{\Theta}}^{*},\boldsymbol{\theta}_{0},\boldsymbol{A},\boldsymbol{B}^{*}\right)\\ &\times \mathbf{P}\left(\boldsymbol{G}_{\widetilde{U}}^{*}\right)\cdot \mathbf{P}\left(\boldsymbol{B}^{*}\right)\cdot \mathbf{P}\left(\widetilde{\boldsymbol{\Theta}}^{*}\right), \end{align*}
  $q\left (\boldsymbol{B}^{*} \mid - \right )$, $q (\widetilde{\boldsymbol{\Theta }}^{*} \mid \boldsymbol{B}^{*}, - )$, and $q\left (\boldsymbol{G}_{\widetilde{U}}^{*} \mid \boldsymbol{B}^{*}, \boldsymbol{\Theta }^{*}, - \right )$are probabilities calculated by the proposal distributions mentioned above.
(2). Propose a sample $s$from $Unif(0,1)$, if $s\leq \alpha $, then we accept $\boldsymbol{B}^{*}$, $\boldsymbol{\Theta }^{*}$, and $\boldsymbol{G}^{*}_{\widetilde{U}}$as new parameter values, otherwise, reject them.

### Algorithm details

We begin by initializing $\boldsymbol{W}_{U}$, $\boldsymbol{G}_{\widetilde{U}}$, $\boldsymbol{A}$, $\boldsymbol{B}$, $\boldsymbol{\Theta }$, $\widetilde{\boldsymbol{\Theta }}$, and $\boldsymbol{\theta }_{0}$. Each element of $\boldsymbol{W}_{U}$(or $\boldsymbol{G}_{\widetilde{U}}$) is drawn from a Bernoulli distribution with parameter $1/2$. Each row of $\boldsymbol{A}$(or $\boldsymbol{B}$) is sampled from a Categorical distribution with equal probabilities. Subsequently, the $j$th column $\boldsymbol{\Theta _{j}}$(or $\widetilde{\boldsymbol{\Theta }}$and $\boldsymbol{\theta }_{0}$) is drawn from a Dirichlet distribution, $ Dirichlet(\boldsymbol{1})$.

Following the initialization of all parameters, we sequentially update $\boldsymbol{W}_{U}$, $\boldsymbol{G}_{\widetilde{U}}$, $\boldsymbol{A}$, $\boldsymbol{B}$, $\boldsymbol{\Theta }$, $\widetilde{\boldsymbol{\Theta }}$, and $\boldsymbol{\theta }_{0}$through the full conditional posterior distribution. The first type of shift move is performed every five iterations, while the second type is performed every 10 iterations.

After the burn-in phase, we collect posterior samples for all parameters and latent variables. The point estimates for these parameters are calculated using the maximum a posteriori (MAP) estimation, which selects the parameter values that maximize the joint posterior distribution of parameters. The De-motif Algorithm is outlined below (Algorithm 1).

## Results

### Simulation study

We conducted simulation studies to evaluate the model’s performance by generating data based on true parameter values and subsequently estimating these parameters from the generated data. The effectiveness of the model is assessed by comparing the estimated parameters with the actual values, aiming for close alignment.

We generated sequences of equal length, where the background distribution, $\boldsymbol{\theta }_{0}$, is drawn from a Dirichlet distribution, $\textrm{Dirichlet}(\boldsymbol{1})$. The first binding motif distribution, $\boldsymbol{\Theta }_{j}$, is sampled from $\textrm{Dirichlet}(\eta )$, while the second binding motif distribution, $\widetilde{\boldsymbol{\Theta }}_{j}$, follows $\textrm{Dirichlet}(\gamma )$. The motif lengths are set to 9 for the first binding process and 5 for the second. The binding site locations within the sequences are uniformly distributed, represented by matrices $\boldsymbol{A}$and $\boldsymbol{B}$. 
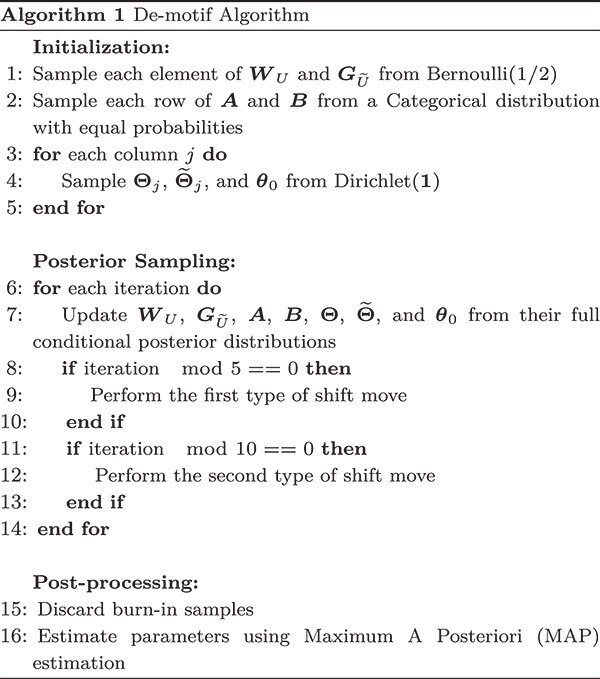


The Bernoulli parameters governing the generation of binding labels $\boldsymbol{G}$and $\boldsymbol{W}$are both set to 0.3. Additionally, we introduce label masking in $\boldsymbol{G}$, where some labels are designated as “NA” to simulate missing or unobservable data. The missing proportion of $g_{i}$for $w_{i} = 1$is 0.1, while for $w_{i} = 0$, the missing proportion is denoted as $\lambda $. This setup reflects realistic biological conditions, particularly the challenges in obtaining data for the second binding process when the first binding fails.

We conduct the MCMC algorithm using noninformative (uniform) priors, which aim to assign equal probability to all possible parameter values within a given range. This minimizes the influence of subjective assumptions and ensures that the posterior distributions are primarily shaped by the observed data. As a result, our inference remains data-driven and objective, allowing the results to faithfully reflect the underlying patterns in the data. We perform 100 iterations, with the first 50 iterations designated as the burn-in period.

Model performance is evaluated through several key metrics. The error curves for $\boldsymbol{\theta }_{0}$, $\boldsymbol{\Theta }$, and $\widetilde{\boldsymbol{\Theta }}$illustrate the normalized L1 norm of the absolute errors over time, indicating estimation precision. The normalized L1-norm represents the average absolute error per element, obtained by summing the absolute errors across all components and dividing by the total number of elements. The accuracy curves for the latent variables $\boldsymbol{G}$, $\boldsymbol{A}$, and $\boldsymbol{B}$represent the proportion of correct predictions, reflecting the model’s effectiveness in identifying these hidden variables. The likelihood curve, which plots log-likelihood values across iterations, serves as an indicator of model fit, where stability and an upward trend suggest good convergence. Additionally, sequence logos of the estimated motifs compared with the true motifs provide a visual assessment of motif estimation accuracy.

To demonstrate performance under different missing proportions $\lambda $and Dirichlet parameters $\eta $and $\gamma $, we fix the number of sequences at 200 and sequence length at 15. The possible missing proportions of $g_{i}$for $w_{i} = 0$, denoted as $\lambda $, take values in $\{0, 0.5, 1\}$. The Dirichlet parameter $\eta $for the first binding motif distribution assumes values in $\{0.05, 0.1, 0.2\}$across different simulations, while $\gamma $, the parameter for the second binding motif distribution, also takes values in $\{0.05, 0.1, 0.2\}$. These variations simulate different levels of motif conservation—lower values (e.g. 0.05) lead to more concentrated distributions dominated by a few AAs, whereas higher values (e.g. 0.2) result in flatter distributions.

**Figure 3:**
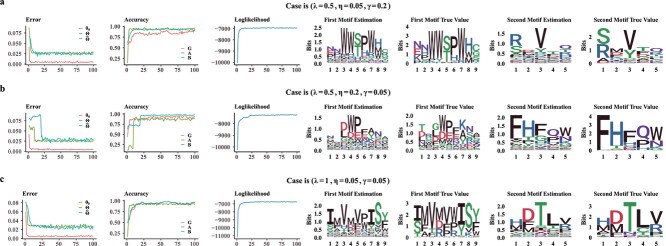
**Simulation results.**The first column presents trace plots of errors, where red, green, and blue curves correspond to $\boldsymbol{\theta }_{0}$, $\boldsymbol{\Theta }$, and $\widetilde{\boldsymbol{\Theta }}$, respectively. The second column shows accuracy trace plots for $\boldsymbol{G}$(red), $\boldsymbol{A}$(green), and $\boldsymbol{B}$(blue). The third column illustrates the log-likelihood trace plots. The fourth through seventh columns display sequence logos: estimated $\boldsymbol{\Theta }$, true $\boldsymbol{\Theta }$, estimated $\widetilde{\boldsymbol{\Theta }}$, and true $\widetilde{\boldsymbol{\Theta }}$, respectively.

Due to the page limit, we only present three cases in the main content, with the corresponding simulation results shown in Fig. 3. Each case is characterized by a tuple $(\lambda , \eta , \gamma )$, where $\lambda $represents the proportion of unknown $\boldsymbol{G}$labels, while $\eta $and $\gamma $are the Dirichlet parameters for the first and second binding motifs, respectively. Results for other cases are provided in the supplementary material. From Fig. 3, we observe that the model performs well across different scenarios. Notably, even when the missing proportion of $g_{i}$for $w_{i} = 0$reaches 1, the model successfully identifies the second binding motif.

The second row of Fig. 3illustrates a scenario where the first binding motif exhibits lower conservation (i.e. accommodates a broader range of AAs), while the second motif demonstrates higher conservation (i.e. dominated by specific AAs). Notably, during the iterations, significant “jumps” occur in the error and accuracy curves—specifically, for the first motif’s position matrix $\boldsymbol{A}$at time = 10 and for the second motif’s position matrix $\boldsymbol{B}$at time = 20. These jumps are driven by the two shift moves.

**Figure 4:**
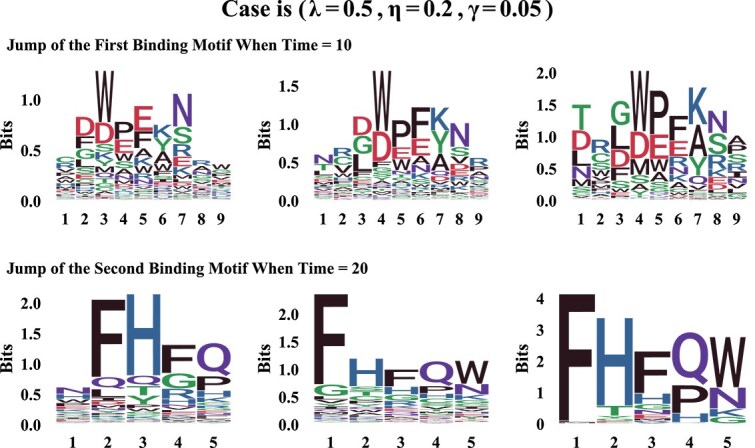
**Sequence logos at “jump” events.**Each row represents a specific time point associated with a jump event for the first binding motif (at time = 10) and the second binding motif (at time = 20). Within each row, the three plots depict the motif before the jump, the motif after the jump, and the true motif, respectively.

Figure 4illustrates motif changes during these jump events. Each row corresponds to a specific time point, showing the motif before the jump, after the jump, and the true motif. Comparing the first and second columns reveals the impact of the jump event on the motif distribution, while the third column provides a reference for evaluating accuracy. These visual comparisons highlight the significant role of MH shift moves in enhancing model performance.

**Figure 5:**
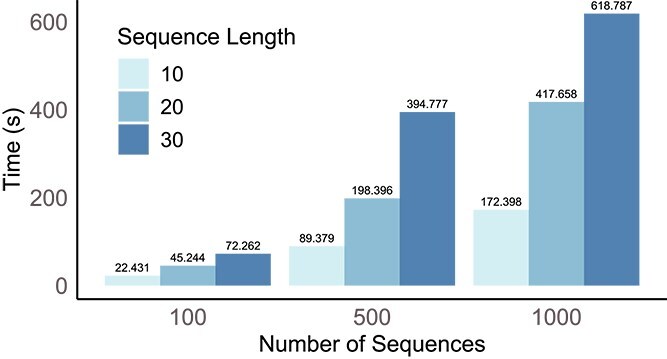
**Runtime across different dataset sizes and motif lengths.**The number on top of each bar represents the running time in seconds.

We conducted additional simulation studies using varying sequence counts and lengths. Specifically, we evaluated performance with 100, 500, and 1000 sequences and sequence lengths of 10, 20, and 30, respectively. The tests were performed with fixed values $\lambda = 1$, $\eta = 0.05$, and $\gamma = 0.05$. Detailed performance results are provided in the supplementary. Additionally, we recorded runtime under these conditions (Fig. 5). The evaluations were conducted on a 12th Gen Intel(R) Core(TM) i7-12700, 2.1 GHz CPU. Our method exhibits approximately linear scaling with both the number of sequences and motif length.

Overall, these findings demonstrate the robustness and efficiency of the De-motif algorithm in exploring complex parameter spaces. For detailed results on additional cases, please refer to the supplementary.

### Real applications

After the evaluation by the simulation studies, we applied our model to the analysis of T cell recognition processes.

#### Decomposition of T Cell Response Motif

We aim to decompose the T Cell Response Motif into two key components: the TCR Recognition Motif and the MHC B&P (Binding and Presentation) Motif. To accomplish this, we apply a labeling system to the peptide sequences. Specifically, peptide sequences capable of binding to MHC and being presented on the cell surface are assigned the label $ w_{i}=1 $(MHC B&P Motif). Furthermore, peptide sequences that can bind to TCRs are given the label $ g_{i}=1 $(TCR Recognition Motif). Therefore, the peptide sequences, which can trigger T cell response, are labeled as $w_{i}=1$and $g_{i}=1$. The peptide sequences, which cannot trigger T cell response but can be bound to MHC and presented, are labeled as $w_{i}=1$and $g_{i}=0$.

**Figure 6:**
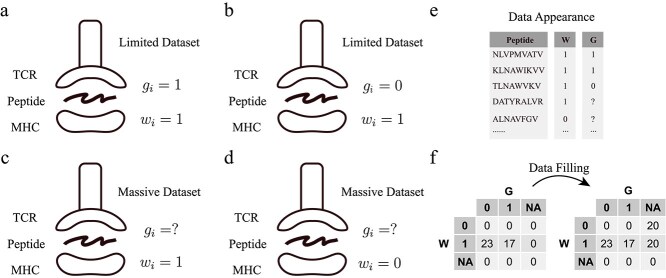
**Data appearance. a, b**. T cell response data. The amount of this type of data is limited. **c, d**. MHC EL data. The amount of MHC EL data is large. **e**. Data appearance. The first column represents the sequences. The rest two columns are labels. **f**. Contingency tables. The left table presents the original data distribution, while the right table shows the distribution after filling in MHC EL data.

In the context of research papers, there is often a focus on reporting positive outcomes, particularly concerning sequences that are presented by MHC and trigger immune responses. However, sequences that are presented by MHC but fail to induce immune responses are less frequently documented, despite their potential value in training machine learning algorithms. In this study, we leveraged a dataset of Vaccinia Virus (VACV) peptides that includes this type of data [19, 20]. All peptides in this dataset were tested for T cell immune responses in infected mice and were confirmed to be eluted from MHC molecules, indicating their binding to and presentation by MHC. We focused our study on the MHC allele H2-Kb. Each sequence in this dataset was labeled $ w_{i}=1 $and either $ g_{i}=1 $or $ g_{i}=0 $, based on their immunogenicity (Fig. 6a,b). The dataset comprises 40 sequences, with 23 being non-immunogenic and 17 immunogenic.

To complement this dataset, additional data were necessary, specifically sequences where $ w_{i} $is zero, indicating no MHC binding or presentation. For this purpose, we accessed the eluted ligand (EL) dataset from the training set of NetMHCpan-4.1 [21], selecting sequences corresponding to the same MHC allele, H2-Kb. This dataset contains >10 000 sequences, providing information on whether peptides can bind to MHC and be presented. To maintain balance in our analysis, we randomly selected 40 sequences from this set, with an equal split of 20 positive (bound and presented) and 20 negative (neither bound nor presented). Because the MHC EL data were not tested for T cell immune response, the label $ g_{i} $is omitted for these sequences (Fig. 6c,d,f).

We defined the length of the MHC B&P Motif $ J $as 9, which is consistent with the commonly accepted size for MHC-related motifs. The length of the TCR Recognition Motif $ \tilde{J} $, however, remains undetermined. To address this, we experimented with various lengths for the TCR Recognition Motif and assessed their performance to select the most appropriate size. The possible peptide lengths range from 2 to 9, with 9 chosen as the maximum because peptides must first bind to MHC before being recognized by TCR, and MHC (H2-Kb) preferentially binds to peptides of this length. Consequently, nearly all sequences in the dataset are 9 AAs. Specifically, out of 80 sequences, 73 are exactly 9 AAs long, while only seven exceed this length. Due to the limited number of longer sequences, results for peptides longer than 9 may not be reliable.

Given that the actual TCR Recognition Motif is unknown, we implemented a strategy to mask $\sim $20% of the known $ G $labels. Then we utilized the MAP algorithm to predict these masked labels. The accuracy of these predictions served as the primary criterion for selecting the optimal motif length.

**Figure 7:**
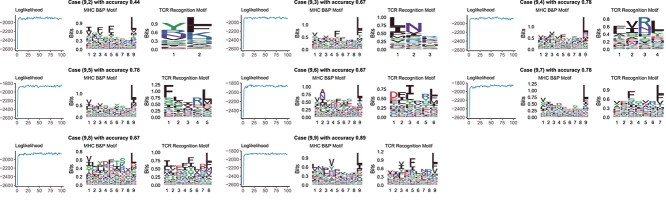
**Results for the decomposition of T Cell Response Motif.**The MHC B&P Motif is fixed at a length of 9, while the TCR Recognition Motif varies in length from 2 to 9. The subtitle provides the prediction accuracy after manually masking the variable $G$.

The hyperparameters for the prior distributions were maintained consistent with those used in the simulation studies. The performance of different motif lengths was detailed in Fig. 7. We selected a length of 9 for the TCR Recognition Motif, as it yielded the highest prediction accuracy, recorded at 0.89.

#### Comparisons with existing methods

To further evaluate our method, we compared it with two immunogenicity prediction algorithms: DeepNeo [22] and RRIME [23], both of which provide an immunogenic score for peptide to trigger TCR recognition. Using DeepNeo and RRIME, we assessed the scores for the masked 20% of known $ G $labels. A threshold was then applied to define the predicted $ G $labels based on these immunogenic scores, and the predicted labels were compared with the actual $ G $labels to determine accuracy.

For the DeepNeo algorithm, scores were only available for peptides of length 9. Since some of the masked $ G $label sequences exceeded this length, we divided these longer sequences into smaller peptides of length 9. Each of these smaller peptides was processed using DeepNeo to generate a score, and the average score of these smaller peptides was assigned as the score for the original sequence. If a default threshold of 0.5 was used, DeepNeo achieved an accuracy of 0.5556. By varying the threshold from 0 to 1 in increments of 0.001, the highest accuracy achieved by DeepNeo was 0.6667.

Similarly, the RRIME algorithm was used to generate scores for peptides with lengths ranging from 9 to 14. When a threshold of 0.5 was applied, RRIME achieved an accuracy of 0.5556. By adjusting the threshold from 0 to 1 in increments of 0.001, the highest accuracy attained by RRIME was also 0.6667. In contrast, our algorithm achieved an accuracy of 0.89, outperforming both DeepNeo and RRIME.

To further validate our model’s generalizability, we evaluated it on an independent dataset [24] comprising peptides derived from recombinant vesicular stomatitis virus pseudotyped with glycoprotein (VSV-GP). This dataset, consisting of 24 sequences (20 non-immunogenic and 4 immunogenic), was not used in model training or development, serving as an unbiased external benchmark. Following the same evaluation procedure, DeepNeo and RRIME both achieved a maximum accuracy of 0.8333, while our method outperformed them with an accuracy of 0.875.

#### Decomposition of MHC B&P Motif

Our next objective is to decompose the MHC B&P Motif into the MHC Binding Motif and the MHC Presentation Motif. This involves categorizing peptide sequences based on their ability to bind to MHC and their capacity to be presented by MHC. Specifically, we assign the label $ w_{i}=1 $to peptide sequences that can bind to MHC (MHC Binding Motif). Peptide sequences that MHC can present are labeled $ g_{i}=1 $(MHC Presentation Motif). Thus, sequences that both bind to and are presented by MHC are doubly labeled $ w_{i}=1 $and $ g_{i}=1 $. Conversely, sequences capable of binding to MHC but not presented are labeled $ w_{i}=1 $and $ g_{i}=0 $.

For this analysis, we utilized the EL data that were selected during the previous phase of decomposition. In this dataset, sequences labeled originally with EL 0, which indicates that MHC does not present them, were assigned $ g_{i}=0 $and $ w_{i} $was set to “NA.” These labels indicate that while these sequences are not presented by MHC, it does not necessarily mean they are incapable of binding to MHC. On the other hand, sequences that were originally labeled with EL 1, indicating successful MHC presentation, received both $ g_{i}=1 $and $ w_{i}=1 $.

To enhance our dataset and provide a more robust analysis, we incorporated additional data concerning $ w_{i} $, specifically targeting the binding affinity of peptides to MHC. We accessed the binding affinity (BA) dataset from the training set of NetMHCpan-4.1 [21], which contains continuous scores that quantify the strength of MHC-peptide binding. These scores are crucial as they directly relate to the likelihood of a peptide’s ability to bind to MHC molecules. To convert these continuous scores into binary labels indicative of binding status, we applied a threshold defined in a previous study [25] for the corresponding MHC allele. This threshold specifies the minimum score required for a peptide to be considered capable of binding to the MHC.

To ensure a balanced analysis, we randomly selected 40 sequences from the MHC BA data, with 20 labeled as negative (not meeting the binding threshold) and 20 as positive (meeting or exceeding the binding threshold). Since the MHC BA dataset does not provide information on whether the peptides are presented by MHC, the label $ g_{i} $was designated as “NA” for these sequences.

**Figure 8:**
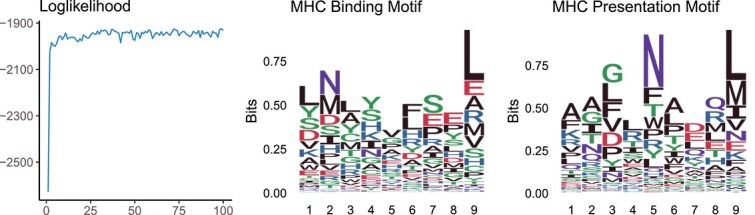
**Results for the decomposition of MHC B&P Motif.**The lengths of MHC B&P Motif and TCR Recognition Motif are both fixed to 9.

Both the MHC Binding Motif and the MHC Presentation Motif were set at a length of 9. To evaluate the effectiveness of our model, we implemented a procedure where 20% of the known $ G $values were manually masked, and predictions were made using MAP prediction. The results of this analysis are illustrated in Fig. 8, where the prediction accuracy was found to be 0.7. This level of accuracy indicates a reasonable degree of reliability in our model’s ability to predict and differentiate between the binding and presentation stages of peptides for MHC.

#### Hierarchy of motifs

**Figure 9:**
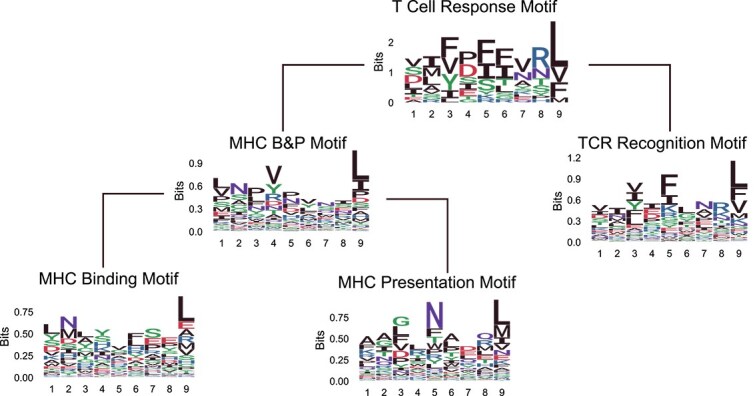
**Hierarchy of motifs.**T Cell Response Motif is a composite of MHC B&P Motif and TCR Recognition Motif. MHC B&P Motif is a composite of MHC Binding Motif and MHC Presentation Motif.

Combining the results from the two decomposition phases, we present the hierarchical organization of motifs in Fig. 9. At the top of this hierarchy, the T Cell Response Motif is derived from sequences that successfully trigger a T cell response in the VACV dataset.

The figure shows that the AA “F” frequently appears at three positions within the T Cell Response Motif. Upon decomposition, these occurrences of “F” are concentrated at the fifth position in the TCR Recognition Motif, highlighting its specificity in TCR recognition. Additionally, the MHC B&P Motif shows a distinct preference for the AA “V” at its fourth position. These observations validate the rationality of our decomposition approach, illustrating that the MHC B&P Motif and TCR Recognition Motif exhibit specific and different preferences at certain positions, aligning with their distinct functional roles.

In further analyzing the decomposition of the MHC B&P Motif, we note specific AA preferences that emerge distinctly in the MHC Binding and Presentation Motifs. For instance, the MHC Binding Motif prefers the AA “N” at the second position, a residue commonly recognized as an anchor in MHC binding processes [26]. In contrast, the MHC Presentation Motif shows a preference for “N” at the fifth position. We found that the same location can have different importance in the MHC binding and presentation processes. De-motif sampling effectively captures and clearly illustrates these differences.

## Discussion

In this study, we introduced an innovative approach to decompose hierarchical motifs, validated through simulation studies and real-world application to T cell recognition problems. We defined five distinct motifs with varying functionalities and established a clear hierarchy among them, as illustrated in Fig. 9. Given the novelty of some motifs, optimal lengths, and sequence logos were initially unknown and had to be determined computationally. We achieved this by setting a portion of the known $ G $labels to “NA” and employing MAP prediction to derive the most effective motif lengths. The prediction accuracy for the T Cell Response Motif and MHC B&P Motif was found to be 0.89 and 0.7, respectively, with the optimal length for the TCR Recognition Motif determined to be 9.

Note that TCR recognition and TCR binding are not strictly equivalent. TCR recognition involves downstream signaling events leading to immune activation, whereas TCR binding refers solely to the physical interaction between a TCR and a pMHC complex. Some strongly bound peptides fail to trigger T cell responses, while moderately binding peptides can induce activation. However, most TCR specificity databases, such as VDJdb [27] and McPAS-TCR [28], as well as widely used prediction algorithms like ERGO2 [29], pMTnet [30], DLpTCR [31], and PanPep [32], do not differentiate between binding and functional assays. Explicit assay information is needed for further analysis and model refinement.

De-motif Sampling outperforms traditional motif inference methods by leveraging hierarchical relationships between binding events, which traditional motif discovery approaches. Unlike standard methods that treat motifs independently, our model explicitly incorporates dependencies, making it particularly effective in cases like T cell recognition, where MHC binding is a prerequisite for TCR recognition. Traditional approaches require separate, well-labeled datasets ($w_{i}=1,~g_{i}=0$, and $w_{i}=0,~g_{i}=1$), but in biological scenarios where sequences labeled as “$w_{i}=0, g_{i}=1$” may not exist, they fail to infer TCR-specific motifs independently. De-motif sampling overcomes this limitation by utilizing Bayesian inference to extract motif information even in the absence of direct observations, allowing it to make use of all available data rather than analyzing subsets in isolation. Additionally, our model introduces two novel MH shift moves that improve sampling efficiency and prevent the algorithm from getting trapped in local optima, leading to more accurate and robust motif discovery.

The de-motif sampling technique not only provided the position-specific probability matrices for hierarchical motifs but also enabled the generation of peptide sequences detached from one of the underlying motifs. These sequences can be essential for specific binding, such as TCR recognition, free from MHC restriction influences. This capability can improve T cell epitope prediction, allowing researchers to separately analyze sequences bound and presented by MHC and those recognized by TCRs. A prediction pipeline trained on these two types of independent data could potentially enhance the performance and accuracy of immune response predictions.

However, this study faces limitations due to the scarcity of suitable data. The primary dataset utilized, the VACV dataset, lacks detailed descriptions for TCR, suggesting that the derived TCR Recognition Motif may represent a composite of multiple TCRs. This could dilute the specificity of AA preferences at each position. Ideally, a dataset encompassing a single TCR and MHC pair with adequate positive and negative labels would be more suitable. Although databases such as NeoTCR [33], VDJdb [27], and IEDB [34] provide some peptide-TCR pairings under a single TCR and MHC context, the number of associated epitopes remains too limited for robust motif inference. Even the most frequently observed TCR in VDJdb is linked to only 12 unique epitopes, which constrains the performance of de-motif sampling. To address these challenges, we plan to enhance de-motif sampling by incorporating mixture motifs in the future.

For MHC binding, we assume that the binding positions form a contiguous AA stretch, which is consistent with known structural data. For TCR binding, while interaction sites may be spatially dispersed due to TCR loop flexibility [35], we approximate them as a contiguous motif to facilitate computational modeling. This simplification is a common practice in motif discovery for short peptide sequences and does not preclude future extensions to incorporate noncontiguous binding sites. Future work could extend our model to incorporate noncontiguous binding sites, potentially improving accuracy in capturing the interaction patterns between TCRs and peptides.

**Key Points**

- We propose a rigorous statistical model specifically designed to decompose hierarchical motifs, enabling the identification of motifs in sequences that carry dual labels.
- De-motif sampling incorporates two innovative shift moves to avoid local optima, and the effectiveness has been validated through extensive simulation studies.
- This study establishes a clear hierarchical structure for motifs related to T cell recognition. It marks the first attempt to derive the TCR recognition motif without MHC restrictions, potentially aiding in the refinement of T cell epitope prediction by enabling separate analyses of MHC-bound and TCR-recognized sequences.

## Supplementary Material

supplementary_250225_bbaf221

## Data Availability

The raw data and the source codes are available on GitHub (https://github.com/RanLIUaca/Demotif).
