# Supplementary material for: De-motif sampling: an approach to decompose hierarchical motifs with applications in T cell recognition
Source: Brief Bioinform. 2025 May 16;26(3):bbaf221. doi: 10.1093/bib/bbaf221 (PMC12082833; doi:10.1093/bib/bbaf221)
Supplement: supplementary_250225_bbaf221 [file supplementary_250225_bbaf221.pdf]

Supplementary Materials for
“De-motif Sampling: An Approach to Decompose Hierarchical Motifs with Applications in T Cell Recognition”

1. Performance under Different Missing Proportions and Dirichlet Parameters

The absolute errors for $\widehat{\boldsymbol{\theta}}_0$, $\widehat{\boldsymbol{\Theta}}$, and $\widehat{\widetilde{\boldsymbol{\Theta}}}$ in the Simulation section are reported in Tables 1 through 9. Figures 1 through 3 depict the mean error curves for parameters $\boldsymbol{\theta}_0$, $\boldsymbol{\Theta}$, and $\widetilde{\boldsymbol{\Theta}}$; accuracy curves for latent variables $\boldsymbol{G}$, $\boldsymbol{A}$, and $\boldsymbol{B}$; the likelihood curve; and the estimated values alongside true values for both $\boldsymbol{\Theta}$ and $\widetilde{\boldsymbol{\Theta}}$ under varying values of λ, $\boldsymbol{\eta}$ and $\boldsymbol{\gamma}$. The specific values of λ, $\boldsymbol{\eta}$ and $\boldsymbol{\gamma}$ are listed by the row name for each row. Figures 4 to 11 illustrate the jumps for the first and second motifs obtained using the MH algorithm under various cases of λ, $\boldsymbol{\eta}$ and $\boldsymbol{\gamma}$.

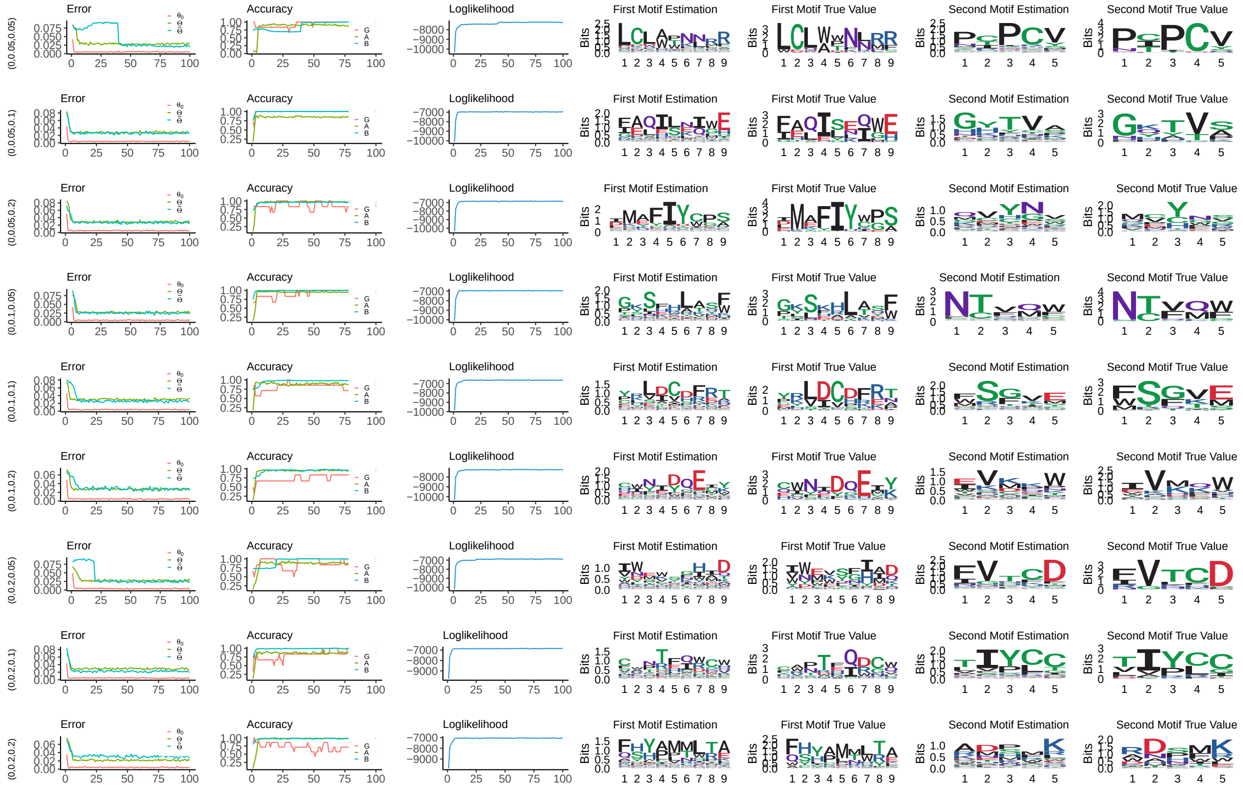

Figure 1: Results for the case of $\lambda = 0$.

Table 1: $\lambda = 0$ and $\boldsymbol{\eta} = 0.05$

$\boldsymbol{\gamma} = 0.05$

		$\lvert\widehat{\boldsymbol{\Theta}}_j - \boldsymbol{\Theta}_j\rvert$									$\lvert\widehat{\widetilde{\boldsymbol{\Theta}}}_j - \widetilde{\boldsymbol{\Theta}}_j\rvert$				
Letter	$\lvert\widehat{\boldsymbol{\theta}_0} - \boldsymbol{\theta}_0\rvert$	$j=1$	$j=2$	$j=3$	$j=4$	$j=5$	$j=6$	$j=7$	$j=8$	$j=9$	$j=1$	$j=2$	$j=3$	$j=4$	$j=5$
A	0	0.001	0.004	0.018	0.075	0.002	0.004	0.008	0.005	0.056	0.017	0	0.022	0.013	0.001
C	0	0.021	0.231	0.01	0.001	0.008	0.009	0.013	0	0.011	0.005	0.027	0.018	0.279	0.012
D	0.001	0.036	0.025	0.068	0.008	0.067	0.004	0.02	0.046	0.003	0.016	0.002	0.021	0.009	0.009
E	0.007	0.006	0.02	0.013	0	0.001	0.032	0.012	0.003	0.015	0.008	0.009	0.004	0.003	0.003
F	0.011	0	0.013	0.041	0.015	0.011	0.01	0.019	0.004	0.092	0.001	0.003	0.005	0.01	0.019
G	0.004	0.011	0.016	0.003	0.01	0.01	0.018	0.002	0.021	0.022	0.006	0.009	0.005	0.013	0.003
H	0.006	0.006	0.007	0.006	0.017	0.013	0.01	0.007	0.012	0.009	0.016	0.027	0.006	0.003	0.005
I	0.001	0.001	0.007	0	0.005	0.002	0.005	0.011	0.017	0.009	0.01	0.119	0.013	0.01	0.001
K	0.001	0.004	0.053	0.019	0.008	0.002	0.069	0.03	0.065	0.005	0.01	0.004	0.006	0.015	0.026
L	0.002	0.151	0.005	0.226	0.009	0.002	0.024	0.142	0.043	0.002	0.02	0.004	0.008	0.025	0.003
M	0.003	0.018	0.022	0.008	0.029	0.029	0.005	0.014	0.083	0.007	0.054	0.011	0.002	0.008	0.029
N	0.001	0	0.023	0.002	0.016	0.001	0.263	0.019	0.039	0.01	0.004	0.015	0.022	0.026	0.017
P	0.01	0.047	0.012	0.025	0.039	0.02	0.022	0.028	0.013	0.005	0.248	0.003	0.157	0.013	0.033
Q	0.006	0.031	0.002	0.033	0.008	0.059	0.012	0.015	0.007	0.006	0.001	0.008	0.016	0.002	0.051
R	0	0.021	0.017	0.02	0.026	0.013	0.007	0.023	0.183	0.074	0.006	0.068	0.028	0.064	0.001
S	0.002	0.001	0.003	0.008	0.008	0.01	0.017	0.019	0.024	0.013	0.038	0.016	0.002	0.022	0.008
T	0.015	0.007	0.001	0.003	0.001	0.002	0.001	0.011	0.009	0.002	0.014	0.072	0.028	0.031	0
V	0	0.016	0.028	0.012	0.012	0.011	0.01	0.007	0.004	0.022	0.023	0.028	0.001	0.009	0.104
W	0.004	0.076	0.021	0.017	0.274	0.096	0.001	0.005	0.022	0.034	0.004	0.008	0.005	0.012	0.001
Y	0.004	0.001	0.048	0.035	0.003	0.015	0.031	0.023	0.01	0.017	0.036	0.01	0.027	0.013	0.001

$\boldsymbol{\gamma} = 0.1$

		$\lvert\widehat{\boldsymbol{\Theta}}_j - \boldsymbol{\Theta}_j\rvert$									$\lvert\widehat{\widetilde{\boldsymbol{\Theta}}}_j - \widetilde{\boldsymbol{\Theta}}_j\rvert$				
Letter	$\lvert\widehat{\boldsymbol{\theta}_0} - \boldsymbol{\theta}_0\rvert$	$j=1$	$j=2$	$j=3$	$j=4$	$j=5$	$j=6$	$j=7$	$j=8$	$j=9$	$j=1$	$j=2$	$j=3$	$j=4$	$j=5$
A	0	0.002	0.025	0.023	0.001	0.002	0	0.024	0	0.027	0.007	0.012	0.08	0.008	0.01
C	0.001	0.002	0	0.008	0.019	0.001	0.012	0.019	0.001	0.008	0.005	0.037	0.025	0.038	0.018
D	0.004	0.003	0.003	0.013	0.018	0.015	0	0.023	0.012	0.008	0.001	0.005	0.011	0.002	0.022
E	0.003	0.004	0.019	0.006	0.004	0.016	0.193	0.022	0.002	0.12	0.042	0.006	0.002	0.009	0.001
F	0.002	0.225	0.028	0.005	0.003	0.012	0.021	0.007	0.001	0.007	0.002	0.012	0.031	0.008	0.003
G	0.002	0.079	0.02	0.001	0.016	0.056	0.006	0.018	0.022	0.015	0.223	0.038	0.002	0	0.054
H	0.004	0.007	0.079	0.008	0.101	0.006	0.002	0.01	0.014	0.074	0.096	0.083	0.016	0.001	0.018
I	0.006	0.039	0.015	0.015	0.27	0.018	0.002	0.032	0.032	0.036	0.003	0.005	0.004	0.034	0.02
K	0	0.037	0.002	0.007	0.021	0.002	0.012	0.005	0.044	0.004	0.011	0.146	0.002	0.008	0.049
L	0.001	0.004	0	0.07	0.013	0.104	0.064	0.003	0.028	0.002	0.018	0.01	0.002	0.021	0
M	0.011	0.019	0.011	0.011	0.011	0.002	0.014	0.013	0.029	0.003	0.017	0.003	0.001	0.003	0.026
N	0.007	0.037	0.022	0.018	0.015	0.027	0.051	0.006	0.035	0.004	0.02	0.001	0.003	0.057	0.004
P	0.005	0.008	0.007	0.002	0.045	0.007	0.021	0.011	0.004	0.003	0.03	0.07	0.024	0.043	0.03
Q	0.002	0.006	0.001	0.106	0.002	0.032	0.029	0.2	0.045	0	0.009	0.133	0.011	0.013	0.01
R	0.003	0.035	0.028	0.007	0.003	0.006	0.011	0.004	0.005	0.015	0.008	0.002	0.041	0.028	0.014
S	0.001	0.012	0.036	0.025	0.01	0.174	0.004	0.018	0.014	0.027	0.006	0.028	0.008	0.025	0.235
T	0.006	0.008	0.06	0.013	0.004	0.029	0	0.017	0.006	0.028	0.044	0.007	0.048	0.141	0.021
V	0.011	0.003	0.003	0.012	0.002	0.028	0.004	0.01	0.012	0.007	0.006	0.008	0.027	0.184	0.003
W	0	0.011	0.004	0.017	0.008	0.001	0.062	0	0.223	0.008	0.005	0.01	0.012	0.022	0.001
Y	0.001	0.005	0.003	0.011	0.001	0.078	0.014	0.034	0.032	0.001	0.001	0.105	0.046	0.004	0.021

$\boldsymbol{\gamma} = 0.2$

		$\lvert\widehat{\boldsymbol{\Theta}}_j - \boldsymbol{\Theta}_j\rvert$									$\lvert\widehat{\widetilde{\boldsymbol{\Theta}}}_j - \widetilde{\boldsymbol{\Theta}}_j\rvert$				
Letter	$\lvert\widehat{\boldsymbol{\theta}_0} - \boldsymbol{\theta}_0\rvert$	$j=1$	$j=2$	$j=3$	$j=4$	$j=5$	$j=6$	$j=7$	$j=8$	$j=9$	$j=1$	$j=2$	$j=3$	$j=4$	$j=5$
A	0.001	0.026	0.023	0.084	0.01	0.045	0.003	0.001	0.04	0.01	0.003	0.003	0.021	0.013	0.016
C	0.022	0.036	0.012	0.013	0.039	0.005	0.026	0.128	0.001	0.011	0.011	0.079	0.067	0.001	0.001
D	0.001	0.012	0.032	0.001	0.002	0.011	0.051	0.014	0.008	0.006	0.009	0.001	0.043	0.008	0.008
E	0.008	0.097	0.024	0.039	0.051	0.005	0.01	0.005	0.009	0.009	0.001	0.029	0.028	0	0.041
F	0.004	0.025	0.022	0.006	0.22	0.001	0.004	0.014	0.008	0.021	0.002	0.011	0.024	0.061	0.054
G	0.005	0.001	0.028	0.012	0.011	0.033	0.01	0.011	0.137	0.009	0.014	0.044	0.009	0.032	0.074
H	0.003	0.05	0.006	0.003	0	0.03	0.055	0.018	0.02	0.007	0.036	0.006	0.049	0.018	0.017
I	0.002	0.006	0.012	0.007	0.006	0.203	0.003	0.043	0.018	0.009	0.007	0.002	0.006	0.004	0.013
K	0	0.009	0.044	0.033	0.028	0.006	0.014	0.094	0.018	0.002	0.014	0.005	0.003	0.001	0.049
L	0.007	0.09	0.029	0.001	0.006	0.015	0.005	0.012	0.013	0.018	0.037	0.02	0	0.031	0.003
M	0.005	0.004	0.298	0.003	0.007	0.008	0.004	0.012	0.012	0.016	0.164	0.034	0.001	0.003	0.035
N	0.004	0.009	0.022	0.001	0.002	0.003	0.006	0.015	0.014	0.001	0.008	0.017	0.04	0.138	0
P	0.003	0.003	0.019	0.006	0.002	0.017	0.009	0.021	0.122	0.019	0.044	0.065	0.002	0.043	0.002
Q	0.004	0.007	0.001	0.088	0.004	0.011	0.005	0.003	0.009	0.014	0.09	0.033	0.015	0.042	0.022
R	0.015	0.019	0.027	0.004	0.01	0.019	0.026	0.013	0.02	0.063	0.033	0.011	0.021	0.003	0.083
S	0.01	0.002	0.011	0.062	0.002	0.001	0.024	0.005	0.008	0.281	0.017	0.008	0.025	0.007	0.022
T	0.004	0.014	0.016	0.018	0.017	0.019	0.022	0.003	0.023	0.013	0.009	0.035	0.009	0.008	0.027
V	0.005	0.019	0.003	0.004	0.012	0.017	0.025	0.028	0.007	0.011	0.025	0.089	0.005	0.001	0.045
W	0.002	0.012	0.004	0.004	0.013	0	0.009	0.157	0.033	0.005	0.023	0.005	0.01	0.088	0.025
Y	0.005	0.041	0.011	0.047	0.018	0.007	0.194	0.018	0.017	0.048	0.042	0.01	0.112	0.039	0.019

Table 2: $\lambda = 0$ and $\boldsymbol{\eta} = 0.1$

$\boldsymbol{\gamma} = 0.05$

		$\lvert\widehat{\boldsymbol{\Theta}}_j - \boldsymbol{\Theta}_j\rvert$									$\lvert\widehat{\widetilde{\boldsymbol{\Theta}}}_j - \widetilde{\boldsymbol{\Theta}}_j\rvert$				
Letter	$\lvert\widehat{\boldsymbol{\theta}_0} - \boldsymbol{\theta}_0\rvert$	$j=1$	$j=2$	$j=3$	$j=4$	$j=5$	$j=6$	$j=7$	$j=8$	$j=9$	$j=1$	$j=2$	$j=3$	$j=4$	$j=5$
A	0.005	0.025	0.01	0.002	0.01	0.105	0.043	0.069	0.006	0.009	0.002	0.006	0.008	0.002	0.001
C	0	0.002	0.02	0.012	0.017	0.019	0.003	0.002	0.015	0.001	0.024	0.093	0.048	0.002	0.076
D	0.002	0.023	0.005	0.012	0.032	0.018	0.052	0.012	0.015	0.004	0.005	0.007	0.002	0.03	0.016
E	0.002	0.005	0.001	0	0.01	0.008	0.007	0.003	0.034	0.001	0.003	0.005	0.015	0.001	0.022
F	0.002	0.003	0.009	0.028	0.07	0	0.001	0	0	0.163	0.025	0.006	0.142	0.012	0.115
G	0.001	0.078	0.005	0.01	0.003	0.002	0	0.003	0	0.001	0.004	0	0.003	0.005	0.042
H	0.001	0.002	0.016	0.008	0.022	0.098	0.01	0.006	0.006	0.051	0.004	0.006	0.004	0.007	0.004
I	0.002	0.036	0.099	0.091	0.023	0.004	0.031	0.03	0.01	0.034	0.007	0.015	0.008	0.005	0.011
K	0.011	0.017	0.066	0.002	0.17	0.059	0.01	0.019	0.018	0.029	0.005	0.009	0.039	0.016	0.012
L	0.013	0.008	0.048	0.119	0.002	0.003	0.243	0.041	0.005	0.002	0.029	0.004	0.002	0.017	0.037
M	0.002	0.003	0.006	0.003	0.023	0.024	0.007	0.002	0.02	0.035	0.022	0.001	0.02	0.014	0.014
N	0.004	0.004	0.007	0.007	0.013	0.004	0.009	0.009	0.004	0.002	0.132	0.006	0.029	0.02	0.002
P	0.002	0.049	0.018	0.014	0.025	0.033	0.023	0.003	0.002	0.008	0.001	0.012	0.016	0.004	0.005
Q	0.005	0.024	0.043	0.027	0.005	0.001	0.002	0.003	0.04	0.003	0.006	0.002	0.028	0.139	0.004
R	0.001	0.007	0.037	0.003	0.002	0.07	0.03	0.017	0.002	0.002	0.008	0.01	0.006	0.003	0.005
S	0.008	0.002	0.029	0.112	0.016	0.001	0.005	0.044	0.002	0.004	0.025	0.002	0.017	0.001	0.031
T	0.008	0.042	0.004	0.018	0.055	0.013	0.002	0.091	0.008	0.006	0.007	0.014	0.004	0.005	0.001
V	0	0.019	0.051	0.002	0.001	0.035	0.001	0.039	0.002	0.011	0.001	0.001	0.099	0.015	0.011
W	0.005	0.012	0	0.009	0.009	0.049	0.007	0.006	0.046	0.038	0.002	0.011	0.004	0.019	0.1
Y	0.006	0	0.045	0.007	0.01	0.041	0.002	0.008	0.034	0.017	0.004	0.005	0.009	0.027	0.004

$\boldsymbol{\gamma} = 0.1$

		$\lvert\widehat{\boldsymbol{\Theta}}_j - \boldsymbol{\Theta}_j\rvert$									$\lvert\widehat{\widetilde{\boldsymbol{\Theta}}}_j - \widetilde{\boldsymbol{\Theta}}_j\rvert$				
Letter	$\lvert\widehat{\boldsymbol{\theta}_0} - \boldsymbol{\theta}_0\rvert$	$j=1$	$j=2$	$j=3$	$j=4$	$j=5$	$j=6$	$j=7$	$j=8$	$j=9$	$j=1$	$j=2$	$j=3$	$j=4$	$j=5$
A	0.015	0.005	0.008	0.025	0.068	0.049	0.014	0.001	0.006	0.039	0.025	0.004	0.014	0.023	0.022
C	0.006	0.031	0.009	0.014	0.034	0.219	0.018	0.007	0.046	0.019	0.009	0.002	0.003	0.02	0.032
D	0.008	0.025	0.03	0.017	0.277	0.02	0.129	0.023	0.044	0.026	0.05	0.002	0.009	0.006	0.002
E	0.001	0.016	0.009	0.091	0.002	0.033	0	0.028	0.089	0.016	0.003	0.003	0.001	0	0.133
F	0.003	0.003	0.024	0.009	0.001	0.006	0.005	0.08	0.055	0.021	0.206	0.003	0.002	0.001	0.035
G	0.004	0.014	0.01	0.02	0.032	0.007	0.025	0.004	0.006	0.003	0.034	0.009	0.115	0.014	0.033
H	0.011	0.023	0.052	0.008	0.011	0.005	0.027	0.037	0.035	0.002	0.026	0.018	0.01	0.002	0.032
I	0.002	0.01	0.009	0.008	0.003	0.055	0.026	0	0.001	0.017	0.005	0.013	0.004	0.044	0.001
K	0.001	0.002	0.06	0.026	0.012	0.006	0.007	0.069	0.088	0.004	0.008	0.017	0.014	0.061	0.002
L	0.006	0.026	0.049	0.139	0.036	0.001	0.001	0.026	0	0.011	0.001	0.014	0.003	0.012	0.006
M	0.001	0.097	0.04	0.003	0.004	0.022	0.018	0.023	0.017	0.011	0.009	0	0.003	0.015	0.073
N	0	0.025	0.002	0.002	0.004	0	0.001	0.001	0.005	0.114	0.005	0.004	0.026	0.02	0.005
P	0.004	0.022	0.006	0.005	0.022	0.002	0.047	0.001	0.003	0.109	0.018	0.023	0.002	0.003	0.019
Q	0.003	0.013	0.019	0	0.005	0.012	0.024	0.002	0.012	0.002	0.024	0.034	0.05	0.013	0.007
R	0	0	0.122	0.002	0.001	0.004	0.012	0.013	0.137	0.107	0.016	0.063	0.005	0.002	0.003
S	0.001	0.002	0.011	0.022	0.001	0.002	0.007	0.013	0.005	0.01	0.049	0.129	0.028	0.015	0.063
T	0.002	0.009	0.003	0.045	0.034	0.022	0.015	0.009	0.005	0.035	0.022	0.043	0.007	0.071	0.049
V	0.004	0.018	0.024	0.092	0.002	0.006	0.027	0.01	0.036	0.057	0.005	0.004	0.006	0.099	0.007
W	0.001	0.015	0.007	0.02	0.006	0.006	0.022	0.019	0.03	0.019	0.047	0.02	0.028	0.053	0.02
Y	0.002	0.05	0.042	0.005	0.004	0.002	0.031	0.01	0.007	0.011	0.005	0.01	0.01	0.018	0.032

$\boldsymbol{\gamma} = 0.2$

		$\lvert\widehat{\boldsymbol{\Theta}}_j - \boldsymbol{\Theta}_j\rvert$									$\lvert\widehat{\widetilde{\boldsymbol{\Theta}}}_j - \widetilde{\boldsymbol{\Theta}}_j\rvert$				
Letter	$\lvert\widehat{\boldsymbol{\theta}_0} - \boldsymbol{\theta}_0\rvert$	$j=1$	$j=2$	$j=3$	$j=4$	$j=5$	$j=6$	$j=7$	$j=8$	$j=9$	$j=1$	$j=2$	$j=3$	$j=4$	$j=5$
A	0.004	0.009	0.008	0.014	0.024	0.018	0.007	0.011	0.005	0	0.051	0.017	0.068	0.029	0.003
C	0.002	0.103	0.079	0.01	0.06	0.002	0.003	0.003	0.065	0.001	0.033	0.009	0.09	0.006	0.01
D	0.002	0.003	0.003	0.015	0.037	0.247	0.005	0.013	0.029	0	0.001	0.029	0.06	0.01	0.002
E	0.005	0.047	0.05	0.003	0.013	0.003	0.019	0.204	0.003	0.022	0.109	0.002	0.003	0.002	0.009
F	0.002	0.028	0.004	0.035	0.032	0.055	0.02	0.011	0.002	0.028	0.007	0.019	0.006	0.046	0.004
G	0.001	0.024	0.022	0.035	0.014	0.006	0.051	0.02	0.001	0.002	0.017	0.006	0.012	0.017	0.005
H	0.002	0.012	0.014	0.001	0.01	0.002	0.021	0.032	0.014	0.007	0.014	0.004	0.061	0.005	0.052
I	0.003	0.005	0.023	0.001	0.027	0.001	0.016	0.002	0.179	0.032	0.14	0.011	0.023	0.059	0.002
K	0.013	0.021	0.001	0.024	0.018	0.015	0.059	0.012	0.016	0.111	0.024	0.029	0.05	0.057	0.058
L	0.002	0	0.011	0.003	0.03	0.005	0.027	0.015	0.058	0.014	0.027	0.005	0	0.03	0.02
M	0.013	0.016	0.005	0.007	0.007	0.028	0.004	0.018	0.004	0.008	0.01	0.011	0.103	0.01	0.041
N	0.001	0	0.011	0.232	0.011	0.015	0.022	0.004	0.001	0.035	0.001	0.006	0.014	0.001	0.027
P	0.002	0.028	0.012	0.001	0.001	0.008	0.033	0.029	0.025	0.009	0.003	0.014	0.009	0.059	0.023
Q	0.001	0.054	0.062	0.025	0.132	0.007	0.08	0.022	0.001	0.036	0.019	0.031	0.051	0.114	0.002
R	0.002	0.002	0.031	0.002	0.049	0.01	0.043	0.016	0.001	0.015	0.018	0.009	0.003	0.001	0
S	0.01	0.006	0.021	0.05	0.019	0	0.013	0.025	0.002	0.035	0.001	0.027	0.021	0.073	0.004
T	0.002	0.042	0.025	0.003	0.007	0.021	0.005	0.008	0.025	0.014	0.014	0.017	0.008	0.016	0.012
V	0.011	0.001	0.025	0.019	0.005	0.028	0.027	0.019	0.023	0.003	0.017	0.177	0.003	0.001	0.063
W	0.003	0.012	0.197	0.017	0.048	0.067	0.057	0.005	0.004	0.078	0	0.001	0.041	0	0.082
Y	0.013	0.006	0.005	0.073	0.009	0.111	0.013	0.004	0.005	0.208	0.005	0.02	0.005	0.008	0.027

Table 3: $\lambda = 0$ and $\boldsymbol{\eta} = 0.2$

$\boldsymbol{\gamma} = 0.05$

		$\lvert\widehat{\boldsymbol{\Theta}}_j - \boldsymbol{\Theta}_j\rvert$									$\lvert\widehat{\widetilde{\boldsymbol{\Theta}}}_j - \widetilde{\boldsymbol{\Theta}}_j\rvert$				
Letter	$\lvert\widehat{\boldsymbol{\theta}_0} - \boldsymbol{\theta}_0\rvert$	$j=1$	$j=2$	$j=3$	$j=4$	$j=5$	$j=6$	$j=7$	$j=8$	$j=9$	$j=1$	$j=2$	$j=3$	$j=4$	$j=5$
A	0	0.012	0.01	0.007	0.021	0.015	0.006	0.009	0.1	0.014	0.002	0.016	0.002	0.004	0.004
C	0.006	0	0.007	0.024	0.004	0.002	0.041	0.005	0.02	0.002	0.01	0.005	0.013	0.179	0.019
D	0.007	0.011	0.022	0.012	0.021	0.045	0.01	0.01	0.072	0.004	0.004	0.006	0.011	0.012	0.162
E	0.008	0.001	0.01	0.104	0.057	0.013	0.001	0.005	0.032	0.011	0.003	0.012	0.004	0.008	0.002
F	0.004	0.002	0.005	0.002	0.037	0.034	0.133	0.003	0.004	0.013	0.015	0.001	0.042	0.045	0.038
G	0.007	0.019	0.014	0.031	0.021	0.01	0.086	0.003	0.035	0.005	0.005	0.008	0.068	0.031	0.014
H	0	0.009	0.003	0.043	0.028	0.007	0.015	0.059	0.053	0.012	0.023	0.013	0.028	0.027	0.003
I	0.003	0.017	0.024	0.002	0.002	0.004	0.029	0.198	0.041	0.007	0.032	0.006	0.008	0.004	0.009
K	0.002	0.025	0.036	0.003	0.079	0.028	0.097	0.015	0.002	0.041	0.001	0.008	0.041	0.045	0.004
L	0	0.012	0.036	0.005	0.006	0.031	0.027	0.013	0.011	0.007	0.001	0.004	0.11	0.011	0.004
M	0	0.022	0.01	0.057	0.035	0.021	0.008	0.053	0.024	0.011	0.022	0.018	0.011	0.045	0.015
N	0.004	0.013	0.072	0.004	0.017	0.004	0.01	0.01	0.025	0.005	0.003	0.027	0.017	0.001	0.01
P	0	0.007	0	0.067	0.029	0.033	0.004	0.016	0.017	0.011	0.005	0.006	0.065	0.008	0
Q	0.001	0.023	0.002	0.005	0.002	0.04	0.017	0.065	0.006	0.099	0.008	0.024	0.005	0.008	0.001
R	0.005	0.015	0.008	0.001	0.004	0.022	0.005	0.017	0.005	0.003	0.126	0.002	0.006	0.003	0.012
S	0.003	0.002	0	0.057	0.054	0.134	0.033	0.02	0.009	0.015	0.025	0.005	0.014	0.006	0.005
T	0.011	0.019	0.009	0.016	0.011	0.015	0.036	0.011	0.011	0.024	0.018	0.011	0.21	0.03	0.041
V	0	0.051	0.015	0.007	0.088	0.031	0.015	0.027	0.002	0.026	0.006	0.179	0.013	0.038	0.009
W	0.002	0.079	0.089	0.027	0.038	0.019	0.003	0.073	0.013	0.032	0.006	0.002	0	0.007	0.005
Y	0.004	0.022	0.027	0.076	0.012	0.065	0.003	0.001	0.032	0.01	0	0.006	0.052	0.006	0.005

$\boldsymbol{\gamma} = 0.1$

		$\lvert\widehat{\boldsymbol{\Theta}}_j - \boldsymbol{\Theta}_j\rvert$									$\lvert\widehat{\widetilde{\boldsymbol{\Theta}}}_j - \widetilde{\boldsymbol{\Theta}}_j\rvert$				
Letter	$\lvert\widehat{\boldsymbol{\theta}_0} - \boldsymbol{\theta}_0\rvert$	$j=1$	$j=2$	$j=3$	$j=4$	$j=5$	$j=6$	$j=7$	$j=8$	$j=9$	$j=1$	$j=2$	$j=3$	$j=4$	$j=5$
A	0.004	0.02	0.068	0.004	0.003	0.053	0.021	0.016	0.002	0.004	0.016	0.037	0.004	0.002	0.003
C	0.003	0.039	0.008	0.009	0.005	0.038	0.048	0.063	0.134	0.018	0.007	0.007	0.001	0.021	0.079
D	0.001	0.037	0.012	0.026	0.035	0.007	0.017	0.292	0.033	0.007	0.043	0.002	0.004	0.01	0
E	0	0	0.044	0.009	0.029	0.046	0.027	0.002	0.05	0.005	0.025	0.01	0.004	0.001	0.002
F	0.009	0.05	0.009	0.007	0.036	0.062	0.018	0.038	0	0.039	0.003	0.002	0.001	0.021	0.003
G	0.003	0.003	0.005	0.002	0.022	0.018	0.005	0.006	0.099	0.031	0.002	0.001	0.007	0.002	0.055
H	0.008	0.037	0.028	0.002	0.009	0.011	0.007	0.034	0.009	0.078	0.003	0.004	0.002	0.004	0.006
I	0.001	0.015	0.009	0.018	0.018	0.026	0.049	0.018	0.031	0.012	0.033	0.086	0.014	0.014	0.058
K	0.009	0.041	0.013	0.005	0.004	0.012	0.017	0.012	0.045	0.003	0.027	0.017	0.033	0.007	0.008
L	0.003	0.002	0.069	0.016	0.007	0.049	0.013	0.004	0.036	0.041	0.035	0.009	0.007	0.094	0.003
M	0.002	0.01	0.026	0.031	0.001	0.042	0.001	0.021	0.004	0.001	0.021	0.007	0.008	0.003	0.019
N	0.002	0.054	0.006	0.02	0.003	0.003	0.001	0.001	0.069	0.002	0.017	0.012	0.023	0.004	0.021
P	0.001	0.04	0.011	0.134	0.003	0.068	0.008	0.023	0.011	0.025	0.021	0.031	0.057	0.015	0.022
Q	0.002	0.039	0.002	0.002	0	0.013	0.268	0.012	0.007	0.164	0.011	0.008	0.01	0.024	0.004
R	0.006	0.017	0.06	0.002	0.094	0.049	0.016	0.036	0.008	0.019	0.01	0.006	0.029	0.045	0.011
S	0.001	0.077	0.065	0.002	0.041	0.011	0.016	0.007	0.001	0.029	0.003	0.04	0.037	0.002	0.026
T	0.008	0.003	0.015	0.017	0.167	0.005	0.011	0.006	0.032	0.016	0.076	0.013	0.009	0.004	0.085
V	0.004	0.058	0.026	0.007	0.002	0.007	0.009	0.008	0.006	0.003	0.117	0.027	0.013	0.006	0.012
W	0	0.006	0.032	0.013	0.042	0.063	0.063	0.13	0.048	0.023	0.006	0.015	0.013	0.007	0.001
Y	0.001	0.05	0.013	0	0.008	0.009	0.022	0.013	0.012	0.001	0.002	0.022	0.038	0.007	0.002

$\boldsymbol{\gamma} = 0.2$

		$\lvert\widehat{\boldsymbol{\Theta}}_j - \boldsymbol{\Theta}_j\rvert$									$\lvert\widehat{\widetilde{\boldsymbol{\Theta}}}_j - \widetilde{\boldsymbol{\Theta}}_j\rvert$				
Letter	$\lvert\widehat{\boldsymbol{\theta}_0} - \boldsymbol{\theta}_0\rvert$	$j=1$	$j=2$	$j=3$	$j=4$	$j=5$	$j=6$	$j=7$	$j=8$	$j=9$	$j=1$	$j=2$	$j=3$	$j=4$	$j=5$
A	0.007	0.018	0.002	0.006	0.052	0.017	0.031	0.001	0.005	0.019	0.142	0.058	0.002	0.008	0.014
C	0.001	0.006	0.02	0.02	0.033	0.003	0.011	0.008	0.006	0.008	0.009	0.004	0.007	0.014	0
D	0	0.001	0.043	0.003	0.006	0.011	0.002	0.02	0.007	0.002	0.049	0.224	0.014	0.019	0.007
E	0.002	0.016	0.002	0.015	0.003	0.011	0.006	0.008	0.014	0.012	0	0.09	0	0.002	0.038
F	0.003	0.083	0.017	0.023	0.039	0.011	0.001	0.027	0	0.022	0.002	0	0.017	0.056	0.004
G	0.004	0.026	0.001	0.027	0.006	0.01	0.009	0.008	0.016	0.013	0.03	0.004	0.001	0.026	0.039
H	0.001	0.013	0.118	0.034	0.021	0.003	0.001	0.032	0.007	0.006	0.027	0.013	0.011	0.011	0.024
I	0.003	0.004	0.003	0.03	0.043	0.002	0.008	0.039	0.014	0	0.014	0.022	0.003	0.02	0.001
K	0.004	0.042	0.032	0.008	0.005	0.009	0.004	0.025	0.001	0.001	0.084	0.01	0.048	0.015	0.068
L	0	0.003	0.009	0.043	0.007	0.007	0.005	0.068	0.025	0.031	0.011	0.026	0.011	0	0.011
M	0.002	0.004	0.026	0.027	0.004	0.154	0.045	0.002	0.025	0.006	0.02	0.11	0.098	0.152	0.004
N	0.002	0.023	0.013	0.008	0.01	0.036	0.058	0.006	0.016	0	0.002	0.058	0.065	0.002	0.025
P	0.002	0.058	0.039	0.007	0.02	0.059	0.011	0.001	0.035	0.024	0.024	0.046	0.031	0.026	0.011
Q	0.001	0.063	0.003	0.014	0.001	0.02	0.013	0.007	0.023	0.016	0.01	0.009	0.001	0.026	0.033
R	0.003	0	0.009	0.012	0.027	0	0.024	0.001	0.038	0.013	0.18	0.002	0.005	0.082	0.02
S	0.002	0.003	0.003	0.002	0.013	0.007	0.034	0.003	0.007	0	0.015	0.003	0.065	0.014	0.002
T	0.02	0.007	0	0.007	0.002	0.008	0.012	0.001	0.069	0.019	0.005	0.026	0.018	0.063	0.015
V	0.013	0.021	0.014	0.018	0.006	0.071	0.026	0.008	0.01	0.003	0.015	0.001	0.028	0.034	0.025
W	0.005	0.053	0.006	0.048	0.01	0.003	0.007	0.162	0.007	0.018	0.103	0.003	0.001	0.008	0.079
Y	0.001	0.011	0.048	0.03	0	0.023	0.016	0.001	0.111	0	0.006	0.002	0.003	0.002	0.003

Table 4: $\lambda = 0.5$ and $\boldsymbol{\eta} = 0.05$

$\boldsymbol{\gamma} = 0.05$															
		$\lvert\widehat{\boldsymbol{\Theta}}_j - \boldsymbol{\Theta}_j\rvert$									$\lvert\widetilde{\widehat{\boldsymbol{\Theta}}}_j - \widetilde{\boldsymbol{\Theta}}_j\rvert$				
Letter	$\lvert\widehat{\boldsymbol{\theta}_0} - \boldsymbol{\theta}_0\rvert$	$j=1$	$j=2$	$j=3$	$j=4$	$j=5$	$j=6$	$j=7$	$j=8$	$j=9$	$j=1$	$j=2$	$j=3$	$j=4$	$j=5$
A	0.01	0.009	0.016	0.006	0.007	0.016	0.009	0.008	0.054	0.003	0.03	0.027	0.001	0.017	0.009
C	0.001	0.029	0.004	0.019	0.009	0.008	0.026	0	0.005	0.004	0.006	0.023	0.005	0.005	0.036
D	0.008	0.009	0.003	0.011	0.004	0.025	0.002	0.004	0.011	0.001	0.095	0.02	0.046	0.004	0.06
E	0	0.008	0.036	0.02	0.002	0.011	0.001	0.133	0.01	0.003	0.021	0.002	0.011	0.006	0.025
F	0.003	0.011	0.044	0.045	0.01	0.007	0.016	0.002	0.015	0.047	0.018	0.005	0.01	0.009	0.158
G	0.001	0.18	0.024	0.005	0.083	0.069	0.029	0.001	0.061	0.017	0.002	0.006	0.002	0.001	0.002
H	0.011	0.024	0.008	0.006	0.002	0.014	0.024	0.096	0.009	0.01	0.012	0.003	0.044	0.003	0.006
I	0.02	0.018	0.004	0.01	0.029	0.018	0.016	0.127	0.009	0.007	0.004	0.045	0.003	0.004	0.007
K	0.006	0.013	0.016	0.041	0	0.001	0.183	0.04	0.01	0.012	0.02	0.023	0.047	0.035	0.016
L	0.008	0.004	0.009	0.012	0	0.002	0.028	0.07	0.116	0.015	0.067	0.024	0.001	0.013	0.122
M	0.003	0.002	0.004	0.014	0.001	0.03	0.028	0.026	0.01	0.023	0.239	0.211	0.006	0.063	0.002
N	0.001	0.001	0.024	0.02	0.021	0.003	0.007	0	0.076	0.001	0.002	0.014	0.008	0.034	0.009
P	0.006	0.014	0.028	0.005	0.022	0.007	0.009	0.013	0.004	0.013	0.014	0.004	0.017	0.007	0.009
Q	0.001	0.005	0.085	0.02	0.028	0.019	0.001	0.064	0.045	0.131	0.006	0.001	0.01	0.008	0.028
R	0.006	0	0.005	0.096	0.006	0.014	0.003	0.031	0.018	0.002	0.032	0.014	0.005	0.006	0.009
S	0.001	0	0.004	0.011	0.247	0.037	0	0.002	0.131	0.018	0.022	0.047	0.007	0.007	0.026
T	0.007	0.025	0.001	0.004	0.002	0.005	0.008	0.012	0.009	0.027	0.027	0.052	0.004	0.072	0.004
V	0.001	0.012	0.027	0.013	0	0.003	0.021	0.018	0.005	0.001	0.001	0.008	0.01	0.014	0.019
W	0.002	0.023	0.026	0.044	0.02	0.005	0.003	0.015	0.006	0.018	0.023	0.006	0.253	0.019	0.009
Y	0.001	0.022	0.054	0.001	0.002	0.28	0.002	0.002	0.011	0.001	0.012	0.01	0.016	0.007	0.005
$\boldsymbol{\gamma} = 0.1$															
		$\lvert\widehat{\boldsymbol{\Theta}}_j - \boldsymbol{\Theta}_j\rvert$									$\lvert\widetilde{\widehat{\boldsymbol{\Theta}}}_j - \widetilde{\boldsymbol{\Theta}}_j\rvert$				
Letter	$\lvert\widehat{\boldsymbol{\theta}_0} - \boldsymbol{\theta}_0\rvert$	$j=1$	$j=2$	$j=3$	$j=4$	$j=5$	$j=6$	$j=7$	$j=8$	$j=9$	$j=1$	$j=2$	$j=3$	$j=4$	$j=5$
A	0.002	0.011	0.007	0.002	0.039	0.003	0.011	0.012	0.035	0.01	0.004	0.002	0.011	0.014	0.017
C	0.001	0.021	0.021	0.001	0.006	0.06	0.005	0.067	0.016	0.018	0.047	0.105	0.008	0.005	0.028
D	0.005	0.001	0.02	0.017	0.038	0.003	0.014	0.015	0.026	0.024	0.004	0.003	0.005	0.006	0.055
E	0.008	0.015	0.001	0.004	0.055	0.002	0.02	0.388	0.029	0.034	0.007	0.01	0.015	0.029	0.002
F	0.003	0.054	0.014	0.014	0.003	0.024	0.004	0.022	0.019	0.022	0.021	0.005	0.002	0.026	0.03
G	0.002	0.039	0.004	0.081	0.013	0.021	0.003	0.012	0.001	0	0.159	0.097	0.009	0.01	0.014
H	0.003	0.004	0.001	0.009	0.01	0.025	0.006	0.001	0.002	0.045	0.014	0.038	0.006	0.013	0.032
I	0	0.217	0.008	0.007	0.019	0.205	0.102	0.007	0.017	0.007	0.003	0.005	0	0.091	0.009
K	0.01	0.01	0.008	0.06	0.241	0.009	0.015	0.043	0.02	0	0.012	0.014	0.003	0.013	0.003
L	0.008	0.021	0.021	0.028	0.005	0.022	0.008	0.006	0.182	0.01	0.022	0.012	0.028	0.003	0.004
M	0.003	0.009	0.004	0.019	0	0.047	0.01	0.034	0.013	0.01	0.013	0.01	0.011	0.007	0.017
N	0.002	0.039	0.015	0.014	0	0.01	0.138	0.024	0.003	0.022	0.004	0.001	0.003	0.029	0.032
P	0	0.011	0.011	0.038	0.021	0.088	0.02	0.026	0.033	0.008	0.016	0.04	0.037	0.006	0.005
Q	0.01	0.01	0.016	0.007	0.002	0.009	0.016	0.029	0.018	0.008	0.005	0.018	0.156	0.04	0.009
R	0.003	0.018	0.005	0.061	0.034	0.018	0.005	0.001	0.001	0.028	0.018	0.006	0.018	0.035	0.014
S	0.009	0.011	0.003	0.001	0.006	0.002	0.004	0.036	0.019	0.011	0.026	0.019	0.005	0.001	0.002
T	0.001	0	0.023	0.001	0.026	0.01	0.008	0.02	0.004	0.041	0.038	0.002	0.005	0.012	0.011
V	0.003	0.002	0.174	0.003	0	0.001	0.019	0.009	0.008	0.009	0.004	0.016	0.019	0.002	0.013
W	0.006	0.037	0.019	0.04	0.006	0.012	0.002	0.008	0.005	0.008	0.024	0.007	0.016	0.016	0.234
Y	0.001	0.007	0.013	0.015	0.033	0.007	0.082	0.02	0.032	0.277	0.02	0.088	0.001	0.043	0.022
$\boldsymbol{\gamma} = 0.2$															
		$\lvert\widehat{\boldsymbol{\Theta}}_j - \boldsymbol{\Theta}_j\rvert$									$\lvert\widetilde{\widehat{\boldsymbol{\Theta}}}_j - \widetilde{\boldsymbol{\Theta}}_j\rvert$				
Letter	$\lvert\widehat{\boldsymbol{\theta}_0} - \boldsymbol{\theta}_0\rvert$	$j=1$	$j=2$	$j=3$	$j=4$	$j=5$	$j=6$	$j=7$	$j=8$	$j=9$	$j=1$	$j=2$	$j=3$	$j=4$	$j=5$
A	0.002	0.008	0.024	0.012	0.015	0.001	0.004	0.016	0.007	0.002	0.048	0	0.022	0.026	0.018
C	0.002	0.013	0.018	0.008	0.001	0.004	0.045	0.002	0	0.113	0.031	0.005	0.027	0.033	0.01
D	0.005	0.015	0.005	0	0.005	0.016	0.005	0.01	0.011	0.007	0.027	0.037	0.001	0.052	0.045
E	0.005	0.064	0.055	0.021	0.003	0.005	0.045	0.018	0.004	0.006	0.022	0.001	0.005	0.003	0.006
F	0.001	0.007	0.034	0.02	0.024	0.011	0.002	0.002	0.005	0.005	0.042	0.003	0.012	0.041	0.007
G	0.002	0.032	0.001	0.003	0.007	0	0.004	0.001	0.001	0.123	0.001	0.009	0.004	0.06	0.001
H	0.014	0.015	0.138	0.003	0.041	0.097	0.004	0.017	0.197	0.043	0.009	0.02	0.019	0.002	0.023
I	0.001	0.007	0.013	0.022	0.005	0.009	0.003	0.001	0.006	0.015	0.015	0.03	0.007	0.026	0.018
K	0.007	0.158	0.004	0.007	0.003	0.003	0	0.027	0.011	0.008	0	0.014	0.03	0.056	0.029
L	0.017	0.019	0.002	0.011	0.008	0.002	0.008	0.007	0.002	0.031	0.015	0.006	0.028	0.017	0.057
M	0.002	0.026	0.019	0.056	0.015	0.021	0.021	0.003	0.01	0.022	0	0.137	0.005	0.03	0.014
N	0	0.093	0.06	0	0.024	0.007	0.005	0.007	0.049	0.007	0.007	0.026	0.008	0.014	0.014
P	0.011	0.077	0.013	0.047	0.003	0	0.171	0.007	0.008	0.007	0.036	0.001	0.005	0.002	0.012
Q	0.003	0.003	0.032	0.021	0.019	0.01	0.005	0.001	0.01	0.014	0.023	0.002	0.031	0.007	0.041
R	0.009	0.014	0.039	0	0.027	0.001	0.025	0.033	0	0.075	0.01	0.031	0.039	0.016	0.002
S	0.001	0.014	0.087	0.008	0.035	0.139	0.033	0.045	0.004	0.077	0.204	0.05	0.029	0.013	0.011
T	0.003	0.001	0.002	0.005	0.018	0.038	0.008	0	0.061	0.027	0.027	0.016	0.015	0.029	0.035
V	0.002	0.007	0.001	0.015	0.008	0.02	0.014	0.018	0.013	0.015	0.008	0.03	0.023	0.02	0.005
W	0.001	0.047	0.002	0.138	0.179	0	0.008	0.245	0.005	0.017	0.01	0.057	0.011	0.016	0.046
Y	0.005	0.009	0.024	0.01	0.081	0.001	0.007	0.029	0	0.01	0.023	0.013	0.136	0.007	0.034

Table 5: $\lambda = 0.5$ and $\boldsymbol{\eta} = 0.1$

$\boldsymbol{\gamma} = 0.05$

		$\lvert\widehat{\boldsymbol{\Theta}}_j - \boldsymbol{\Theta}_j\rvert$									$\lvert\widehat{\widetilde{\boldsymbol{\Theta}}}_j - \widetilde{\boldsymbol{\Theta}}_j\rvert$				
Letter	$\lvert\widehat{\boldsymbol{\theta}_0} - \boldsymbol{\theta}_0\rvert$	$j=1$	$j=2$	$j=3$	$j=4$	$j=5$	$j=6$	$j=7$	$j=8$	$j=9$	$j=1$	$j=2$	$j=3$	$j=4$	$j=5$
A	0.001	0.001	0.011	0.064	0.004	0.079	0.029	0.012	0.014	0.025	0.012	0.001	0.02	0.019	0.006
C	0.002	0.01	0.122	0.005	0.002	0.007	0.01	0.001	0.038	0.011	0.015	0.045	0.024	0.02	0.002
D	0.003	0.04	0.027	0.002	0.021	0.013	0.11	0.009	0.001	0.076	0.026	0.1	0.007	0.103	0.024
E	0.006	0.007	0.021	0.035	0.006	0	0.007	0.015	0.005	0.014	0.008	0.006	0.016	0.043	0.308
F	0.001	0.01	0.004	0.023	0.047	0.058	0.019	0.072	0.013	0.025	0.001	0.024	0.001	0.014	0.096
G	0.01	0.041	0.002	0.01	0.014	0.011	0.003	0.001	0.003	0.007	0.044	0.02	0	0.01	0.022
H	0	0.199	0.007	0	0.003	0.001	0.01	0.015	0.033	0.001	0.001	0.023	0.007	0.018	0.003
I	0.004	0.015	0.022	0.012	0.187	0.015	0.06	0.001	0.045	0.006	0.008	0.001	0.041	0.013	0.019
K	0.002	0.089	0.009	0.009	0.011	0.011	0	0.001	0.018	0	0.002	0.001	0.012	0.001	0.028
L	0.002	0.042	0.003	0	0.045	0.078	0.03	0.104	0	0.031	0.002	0.02	0.011	0.011	0.005
M	0	0.001	0.023	0.008	0.02	0.063	0.062	0.003	0.002	0.003	0.014	0.011	0.028	0.01	0.013
N	0.003	0.009	0.001	0	0.017	0.026	0.005	0.056	0.046	0.004	0.005	0.008	0.005	0.089	0
P	0.002	0.01	0.01	0.008	0.023	0.003	0.017	0.007	0.007	0.011	0.008	0.007	0.016	0.016	0.021
Q	0.004	0.01	0.014	0.005	0.03	0.018	0.004	0.014	0.174	0.004	0.008	0.124	0.001	0.018	0.017
R	0.001	0.034	0.031	0.006	0.015	0.006	0	0.006	0.005	0.024	0.242	0.013	0.201	0.017	0.024
S	0.001	0.005	0.004	0.011	0.007	0.024	0.03	0.037	0.003	0.106	0.014	0.013	0.005	0.008	0.021
T	0.004	0.005	0.028	0.006	0.002	0	0.012	0.01	0.046	0.008	0.005	0.002	0.03	0.007	0.001
V	0.001	0.006	0.004	0.001	0.039	0.014	0.117	0.023	0.03	0.007	0.059	0.009	0.001	0.026	0.003
W	0.004	0.001	0.051	0.011	0	0.035	0.012	0.005	0	0.031	0.001	0.001	0.005	0	0.021
Y	0	0.002	0.024	0.012	0.003	0.046	0.006	0.008	0.096	0.009	0.008	0.016	0.002	0.003	0.027

$\boldsymbol{\gamma} = 0.1$

		$\lvert\widehat{\boldsymbol{\Theta}}_j - \boldsymbol{\Theta}_j\rvert$									$\lvert\widehat{\widetilde{\boldsymbol{\Theta}}}_j - \widetilde{\boldsymbol{\Theta}}_j\rvert$				
Letter	$\lvert\widehat{\boldsymbol{\theta}_0} - \boldsymbol{\theta}_0\rvert$	$j=1$	$j=2$	$j=3$	$j=4$	$j=5$	$j=6$	$j=7$	$j=8$	$j=9$	$j=1$	$j=2$	$j=3$	$j=4$	$j=5$
A	0.002	0.001	0.009	0.029	0.043	0.018	0.004	0.009	0.114	0.205	0.074	0.044	0.024	0.03	0.042
C	0.004	0.026	0.002	0.029	0.001	0.004	0.008	0.098	0.04	0.005	0.161	0.002	0.014	0.004	0.016
D	0	0.004	0	0.031	0.002	0.149	0.015	0.018	0.003	0.013	0.003	0.02	0.019	0	0.001
E	0.001	0.014	0.026	0.051	0.072	0.008	0.082	0.039	0.033	0.003	0.001	0.001	0.092	0.024	0.008
F	0.013	0.02	0.019	0.019	0.012	0.002	0.013	0.014	0.021	0.01	0.002	0.018	0.002	0.004	0.004
G	0.001	0.088	0.002	0.006	0.007	0.015	0.002	0.025	0.001	0.003	0.047	0.014	0.015	0.006	0.015
H	0.008	0.041	0.003	0.001	0.006	0.011	0.06	0.016	0.006	0.002	0.007	0.036	0.033	0.032	0.01
I	0.014	0.011	0.001	0.004	0.018	0.07	0.013	0.01	0.02	0.012	0.007	0.003	0.009	0	0.007
K	0.001	0.009	0.03	0.01	0.012	0.002	0.015	0.005	0.013	0.048	0.024	0.019	0.008	0.016	0.153
L	0.003	0.164	0.082	0.006	0.07	0.008	0.069	0.013	0.008	0.011	0.004	0.129	0.022	0.001	0.006
M	0.001	0.06	0.005	0.001	0.015	0.014	0.009	0.008	0.022	0.015	0.005	0.01	0.001	0.018	0.018
N	0.001	0.002	0.001	0.007	0.001	0.029	0.01	0.009	0.025	0.04	0.006	0.037	0.001	0.018	0.006
P	0.001	0.068	0.029	0.002	0.001	0.078	0.172	0.193	0.025	0.016	0.007	0.003	0.012	0.014	0.035
Q	0.006	0.128	0.006	0.001	0.018	0.012	0.026	0.006	0.001	0.001	0.007	0.022	0.005	0.009	0.006
R	0.002	0.003	0.011	0.013	0.008	0.004	0.007	0.061	0.003	0.011	0.007	0.015	0.012	0.001	0.021
S	0	0.008	0.017	0.002	0.006	0.029	0.006	0.029	0.008	0.074	0.005	0.024	0.043	0.001	0.013
T	0.006	0.002	0.129	0.011	0.071	0.007	0.001	0.005	0.063	0.017	0.002	0.008	0.031	0.191	0.121
V	0.002	0	0.015	0.014	0.008	0.008	0.01	0.002	0.176	0.017	0.027	0.01	0.003	0.013	0.002
W	0	0.029	0.006	0.002	0.373	0.046	0.016	0.027	0.003	0.076	0.116	0.002	0.034	0.023	0.002
Y	0.006	0.026	0.007	0.015	0.002	0.007	0.007	0.032	0.005	0.009	0.023	0.038	0.004	0.023	0.007

$\boldsymbol{\gamma} = 0.2$

		$\lvert\widehat{\boldsymbol{\Theta}}_j - \boldsymbol{\Theta}_j\rvert$									$\lvert\widehat{\widetilde{\boldsymbol{\Theta}}}_j - \widetilde{\boldsymbol{\Theta}}_j\rvert$				
Letter	$\lvert\widehat{\boldsymbol{\theta}_0} - \boldsymbol{\theta}_0\rvert$	$j=1$	$j=2$	$j=3$	$j=4$	$j=5$	$j=6$	$j=7$	$j=8$	$j=9$	$j=1$	$j=2$	$j=3$	$j=4$	$j=5$
A	0.002	0.001	0.013	0.001	0.008	0.205	0.013	0.003	0.006	0.011	0.015	0.001	0.048	0.011	0.021
C	0	0.004	0.243	0.004	0.008	0.014	0.001	0	0.015	0.137	0.005	0.047	0.001	0.001	0.014
D	0.001	0.011	0.028	0.032	0.028	0.005	0.017	0.005	0.121	0.016	0.007	0.021	0.012	0.08	0.018
E	0.001	0.017	0.013	0.141	0.016	0.003	0.023	0	0.039	0.002	0.001	0.031	0.061	0.038	0.005
F	0.012	0.011	0.048	0.013	0.021	0.049	0.032	0.151	0.006	0	0.005	0.023	0.031	0	0.131
G	0.004	0.006	0.011	0.005	0.006	0.009	0.004	0.028	0.124	0.001	0.021	0.015	0.009	0.033	0.014
H	0.001	0.002	0.003	0.014	0.016	0.154	0.037	0.001	0.006	0.02	0.04	0.002	0.025	0.001	0.022
I	0.002	0.016	0	0.01	0.019	0.034	0.008	0.008	0.035	0.007	0.009	0.02	0.004	0.211	0.028
K	0.01	0.015	0.011	0.022	0.008	0.002	0.001	0.024	0.008	0.003	0.006	0.01	0.037	0.024	0.02
L	0.002	0.221	0.009	0.018	0.01	0.01	0	0.018	0.016	0.001	0.018	0.039	0.001	0	0.03
M	0.013	0	0.005	0.005	0.063	0.009	0.011	0.002	0.011	0.005	0.008	0.018	0.015	0.003	0.047
N	0.008	0.001	0.008	0.025	0.004	0.01	0.112	0.003	0.003	0.005	0.027	0.012	0.186	0	0.007
P	0.001	0.041	0.001	0.001	0.009	0.021	0.001	0.001	0.011	0.012	0.078	0.002	0.005	0.003	0.005
Q	0.009	0.01	0.017	0.059	0.014	0	0.004	0.002	0.199	0.017	0.019	0.01	0.019	0.008	0.024
R	0	0.008	0.009	0.037	0.04	0.069	0.023	0.036	0.002	0.012	0.027	0.01	0.02	0.022	0.003
S	0.001	0.002	0.012	0.02	0.011	0.015	0.026	0.01	0.013	0.007	0.013	0.181	0.018	0.019	0.029
T	0.002	0.008	0.01	0.051	0.036	0.016	0.021	0.015	0.006	0.003	0.021	0.045	0.001	0.008	0.043
V	0.007	0.027	0.003	0.002	0.038	0.001	0.005	0.019	0.019	0.032	0.005	0.039	0.002	0.003	0.009
W	0.001	0.07	0.022	0.023	0.011	0.033	0.019	0.003	0.037	0.043	0.024	0.016	0.066	0	0.006
Y	0.013	0.028	0.053	0.165	0.004	0.009	0.007	0.027	0.003	0.002	0.137	0.016	0.004	0.04	0.002

Table 6: $\lambda = 0.5$ and $\boldsymbol{\eta} = 0.2$

Letter	$\lvert\widehat{\boldsymbol{\theta}_0} - \boldsymbol{\theta}_0\rvert$	$\lvert\widehat{\boldsymbol{\Theta}}_j - \boldsymbol{\Theta}_j\rvert$									$\lvert\widehat{\widetilde{\boldsymbol{\Theta}}}_j - \widetilde{\boldsymbol{\Theta}}_j\rvert$				
$\boldsymbol{\gamma} = 0.05$															
Letter	$\lvert\widehat{\boldsymbol{\theta}_0} - \boldsymbol{\theta}_0\rvert$	$j = 1$	$j = 2$	$j = 3$	$j = 4$	$j = 5$	$j = 6$	$j = 7$	$j = 8$	$j = 9$	$j = 1$	$j = 2$	$j = 3$	$j = 4$	$j = 5$
A	0.001	0.009	0.004	0.005	0.004	0.078	0.001	0.029	0.012	0.034	0	0.008	0.012	0.01	0.027
C	0.001	0.102	0.072	0	0.001	0.022	0.021	0.01	0.028	0.015	0.034	0.014	0.049	0.02	0.003
D	0.003	0.107	0.034	0.126	0.042	0.007	0.005	0.001	0.002	0.011	0.02	0.009	0.009	0.018	0.014
E	0.005	0.011	0.017	0.039	0.009	0.028	0.035	0	0.023	0.004	0.013	0.009	0.052	0.031	0.003
F	0.001	0.008	0.029	0.118	0.015	0.002	0.051	0.006	0.007	0.06	0.321	0.013	0.14	0.004	0.014
G	0.005	0.027	0.028	0.105	0.012	0.033	0.013	0.019	0.007	0.059	0.028	0.005	0.121	0.011	0.002
H	0.003	0.003	0.012	0.045	0.005	0.003	0.011	0.004	0.001	0.001	0.004	0.293	0.057	0.013	0
I	0.002	0.029	0.066	0.002	0.001	0.013	0.005	0.029	0.004	0.049	0.057	0.002	0.022	0.015	0.017
K	0.002	0.002	0.001	0.009	0.007	0.018	0.002	0.166	0.034	0.008	0	0.006	0.002	0.002	0.027
L	0.011	0.099	0.022	0.007	0.008	0.007	0.021	0.015	0.005	0.045	0.021	0.008	0.002	0.023	0.02
M	0.005	0.025	0.002	0.001	0.074	0.057	0.008	0.004	0.001	0	0.001	0.005	0.023	0.021	0.034
N	0.011	0.047	0.015	0.037	0.021	0.013	0.015	0.007	0.032	0.027	0.003	0.003	0.027	0.018	0.043
P	0.001	0.032	0.019	0.014	0.019	0.051	0.01	0.035	0.033	0.064	0	0.094	0	0.087	0.009
Q	0	0.025	0.027	0.01	0.001	0.008	0.001	0.035	0.007	0.023	0.017	0.013	0.001	0.206	0.028
R	0.001	0.016	0.089	0.003	0.005	0.003	0.038	0.023	0.081	0.01	0.008	0.009	0.005	0.007	0.003
S	0.003	0.005	0.111	0.056	0.015	0.025	0.017	0.015	0.091	0.081	0.025	0.005	0.012	0.008	0.001
T	0.001	0.188	0	0.001	0.006	0.014	0.005	0.001	0.011	0.01	0.01	0.023	0.04	0.002	0.02
V	0.008	0.009	0.037	0.02	0.001	0.012	0.015	0.032	0.008	0.022	0.049	0.011	0.019	0.065	0.013
W	0.003	0.072	0.011	0.033	0.011	0.042	0.076	0.03	0.032	0.019	0.028	0.027	0.001	0.021	0.207
Y	0.001	0.005	0.017	0.013	0.008	0.01	0.003	0.022	0.017	0.01	0.002	0.059	0.006	0.029	0.014
$\boldsymbol{\gamma} = 0.1$															
		$\lvert\widehat{\boldsymbol{\Theta}}_j - \boldsymbol{\Theta}_j\rvert$									$\lvert\widehat{\widetilde{\boldsymbol{\Theta}}}_j - \widetilde{\boldsymbol{\Theta}}_j\rvert$				
Letter	$\lvert\widehat{\boldsymbol{\theta}_0} - \boldsymbol{\theta}_0\rvert$	$j = 1$	$j = 2$	$j = 3$	$j = 4$	$j = 5$	$j = 6$	$j = 7$	$j = 8$	$j = 9$	$j = 1$	$j = 2$	$j = 3$	$j = 4$	$j = 5$
A	0.004	0.099	0.012	0.022	0.007	0.009	0.032	0.04	0.023	0.005	0.01	0.012	0.014	0.01	0.02
C	0.018	0.002	0.029	0.017	0.004	0.008	0	0.005	0.011	0.006	0	0.031	0.016	0.034	0.009
D	0.013	0.072	0.02	0.009	0.008	0.016	0.029	0.035	0.002	0.133	0.008	0.026	0	0.051	0.105
E	0.011	0.058	0.021	0.003	0.017	0.003	0.003	0.059	0.151	0.017	0.013	0.078	0.062	0.061	0.022
F	0.001	0.052	0.037	0.016	0.027	0.021	0.008	0.009	0.025	0.003	0.002	0.007	0.043	0.01	0.014
G	0.011	0.038	0.038	0.021	0.044	0	0.003	0.011	0.039	0.065	0.014	0.002	0.002	0.147	0.003
H	0	0.022	0.022	0.008	0.056	0.161	0.096	0.069	0.049	0.011	0.117	0	0.002	0	0.002
I	0.002	0.027	0.01	0.007	0.015	0.018	0.088	0.04	0.013	0.015	0.006	0.005	0.043	0.029	0.039
K	0.006	0.001	0.059	0.008	0.01	0.02	0.006	0.005	0.017	0.008	0.005	0.005	0.005	0.01	0.03
L	0.016	0.027	0.001	0.015	0.022	0.001	0.023	0.028	0.085	0.029	0.019	0.037	0.026	0.012	0.079
M	0.006	0	0.026	0.036	0.293	0.002	0.001	0.088	0.068	0.034	0.001	0.005	0.014	0.002	0.018
N	0.013	0.011	0.049	0.071	0.024	0	0.028	0.001	0.057	0.158	0.004	0.023	0.039	0.064	0
P	0.007	0.051	0.033	0.01	0.07	0.022	0.001	0.007	0.005	0.022	0.024	0.011	0.008	0.06	0.029
Q	0.006	0.013	0.019	0.003	0	0.009	0.006	0.008	0.001	0.01	0.007	0.042	0.01	0.001	0.016
R	0.004	0.004	0.03	0	0.017	0.015	0.001	0.001	0.016	0.01	0.013	0.009	0.041	0.061	0.007
S	0.017	0.029	0	0.13	0.014	0.001	0.013	0.004	0.007	0.024	0.006	0.003	0.004	0.017	0.017
T	0.008	0.045	0.116	0.042	0.008	0.016	0.009	0.006	0	0.053	0.004	0.005	0.036	0.001	0
V	0.002	0.002	0.004	0.007	0.019	0.009	0.018	0.002	0.009	0.058	0.011	0.007	0.003	0.009	0.002
W	0	0.032	0.044	0	0.012	0.012	0.006	0.011	0.018	0.001	0.031	0.016	0.023	0.001	0.006
Y	0.01	0.017	0.016	0.011	0.012	0.014	0.038	0.029	0.004	0	0.014	0.028	0.002	0.017	0.017
$\boldsymbol{\gamma} = 0.2$															
		$\lvert\widehat{\boldsymbol{\Theta}}_j - \boldsymbol{\Theta}_j\rvert$									$\lvert\widehat{\widetilde{\boldsymbol{\Theta}}}_j - \widetilde{\boldsymbol{\Theta}}_j\rvert$				
Letter	$\lvert\widehat{\boldsymbol{\theta}_0} - \boldsymbol{\theta}_0\rvert$	$j = 1$	$j = 2$	$j = 3$	$j = 4$	$j = 5$	$j = 6$	$j = 7$	$j = 8$	$j = 9$	$j = 1$	$j = 2$	$j = 3$	$j = 4$	$j = 5$
A	0.002	0.056	0.003	0.002	0.009	0.001	0.001	0.034	0.011	0.044	0.005	0.002	0.018	0.014	0.006
C	0.001	0.001	0.015	0.035	0.076	0.019	0.057	0.006	0.002	0.067	0.092	0.026	0.012	0.099	0.001
D	0.014	0.018	0.015	0.018	0.12	0.015	0.01	0.025	0.012	0.001	0	0.012	0.002	0.002	0.007
E	0.004	0	0.02	0.029	0.101	0.036	0.002	0.012	0.017	0.007	0.006	0.056	0.005	0.001	0.01
F	0.001	0.002	0.014	0.001	0.027	0.045	0.006	0.106	0.001	0.116	0.029	0.023	0.007	0.036	0.01
G	0.001	0.028	0.019	0.024	0.002	0.003	0.006	0.025	0.005	0.012	0.012	0.021	0.034	0.004	0.032
H	0.005	0.003	0.009	0.024	0.001	0.002	0.062	0.033	0.015	0.095	0.02	0.017	0.048	0.042	0.017
I	0.014	0.028	0.004	0.008	0.019	0.051	0.003	0.01	0.046	0.01	0.012	0.001	0.027	0.049	0.02
K	0.002	0.031	0.02	0.001	0.017	0.004	0.001	0.005	0.043	0.003	0.004	0.006	0.002	0.002	0.018
L	0.001	0.107	0.015	0.051	0.01	0.048	0.048	0.001	0.007	0.015	0.093	0.025	0.015	0.009	0.009
M	0.004	0.007	0.008	0.014	0.01	0.021	0.013	0.058	0.023	0.028	0.015	0.02	0.021	0.003	0.003
N	0.009	0	0.075	0.003	0.111	0.008	0	0.05	0.027	0.023	0.039	0.009	0.003	0.02	0.11
P	0.002	0	0.006	0.013	0.034	0.046	0.023	0.008	0.052	0.112	0.015	0.01	0.009	0.001	0.035
Q	0.028	0.02	0.134	0.047	0.027	0.036	0.017	0.025	0.015	0.043	0.017	0.005	0.007	0.005	0.003
R	0.001	0.002	0.006	0.003	0.034	0.009	0.045	0.01	0.025	0.001	0.003	0.004	0.156	0.067	0.002
S	0	0.015	0.002	0.001	0.004	0.001	0.005	0.051	0.041	0.005	0.016	0.001	0.047	0.002	0.018
T	0.004	0.004	0.042	0.208	0.005	0.031	0.043	0.168	0.006	0.016	0.022	0.004	0.018	0.007	0.031
V	0.005	0.035	0.122	0.003	0.003	0.002	0.009	0.018	0.109	0.004	0.002	0.003	0.001	0.08	0.018
W	0.003	0.027	0.09	0.009	0.019	0.013	0.056	0	0.007	0.032	0.009	0.104	0.003	0.033	0.007
Y	0.001	0.001	0.038	0.004	0.042	0.033	0.001	0.031	0.003	0.032	0.116	0.006	0.068	0.021	0.033

Table 7: $\lambda = 1$ and $\boldsymbol{\eta} = 0.05$

$\boldsymbol{\gamma} = 0.05$

		$\lvert\widehat{\boldsymbol{\Theta}}_j - \boldsymbol{\Theta}_j\rvert$									$\lvert\widehat{\widetilde{\boldsymbol{\Theta}}}_j - \widetilde{\boldsymbol{\Theta}}_j\rvert$				
Letter	$\lvert\widehat{\boldsymbol{\theta}_0} - \boldsymbol{\theta}_0\rvert$	$j=1$	$j=2$	$j=3$	$j=4$	$j=5$	$j=6$	$j=7$	$j=8$	$j=9$	$j=1$	$j=2$	$j=3$	$j=4$	$j=5$
A	0.006	0.021	0.015	0.017	0.013	0.018	0.01	0.023	0	0.002	0.101	0.006	0.034	0.006	0.005
C	0.004	0.014	0.019	0.003	0.006	0.002	0.016	0.033	0.019	0.002	0.019	0.057	0.001	0.083	0.051
D	0.002	0.004	0.001	0	0.119	0.003	0.009	0.006	0.009	0.035	0.022	0.053	0.06	0.045	0
E	0.006	0.009	0.013	0.02	0.041	0.118	0.001	0.053	0.002	0.002	0.007	0.005	0.008	0.004	0.03
F	0.003	0.006	0.059	0.073	0.081	0.1	0.007	0.012	0.015	0.014	0.018	0.095	0.03	0.01	0.053
G	0	0.032	0.003	0.004	0.002	0.028	0.002	0.011	0.005	0	0.032	0.026	0.003	0.03	0.024
H	0.003	0.02	0.013	0.053	0.004	0.004	0.006	0.006	0.02	0.013	0.053	0.007	0.007	0.005	0.012
I	0.002	0.06	0.02	0.022	0.002	0.01	0.003	0.083	0.007	0.002	0.053	0.001	0.058	0.015	0.009
K	0.012	0.045	0.058	0.009	0.012	0.001	0.006	0.008	0.04	0.004	0.019	0	0.001	0.007	0.008
L	0.001	0.009	0.01	0.002	0.004	0.015	0.006	0.014	0	0.018	0.002	0.046	0.003	0.154	0.015
M	0.003	0.004	0.212	0.012	0.082	0.002	0.014	0.011	0.006	0.001	0.053	0.156	0.011	0.01	0.011
N	0.002	0.019	0.004	0.005	0.012	0.006	0.025	0.011	0.012	0.007	0.01	0.024	0.055	0.005	0.009
P	0.004	0.024	0.002	0.014	0.004	0.066	0.087	0.03	0.003	0.018	0.003	0	0.002	0.044	0.001
Q	0	0.002	0.02	0.005	0.001	0.01	0.04	0.002	0.003	0.033	0.002	0.001	0.013	0.016	0.01
R	0.004	0.017	0.008	0.005	0.149	0.022	0.078	0.02	0.01	0.026	0.017	0.003	0.013	0.004	0.054
S	0.012	0.239	0.035	0.021	0.021	0.009	0.02	0.038	0.095	0.016	0.014	0.007	0.066	0.015	0
T	0.013	0.076	0.02	0.059	0.005	0.011	0.001	0.006	0.034	0.005	0.002	0.014	0.18	0.027	0.031
V	0.004	0.024	0.011	0.134	0.008	0.07	0.033	0.003	0.052	0	0.023	0.005	0.004	0.005	0.184
W	0.004	0.001	0.023	0.003	0.059	0.005	0.225	0.01	0.062	0.024	0.001	0.004	0.002	0.015	0.021
Y	0.004	0.003	0.027	0.072	0.074	0.006	0.022	0.181	0.005	0.138	0.003	0.02	0.011	0.004	0.022

$\boldsymbol{\gamma} = 0.1$

		$\lvert\widehat{\boldsymbol{\Theta}}_j - \boldsymbol{\Theta}_j\rvert$									$\lvert\widehat{\widetilde{\boldsymbol{\Theta}}}_j - \widetilde{\boldsymbol{\Theta}}_j\rvert$				
Letter	$\lvert\widehat{\boldsymbol{\theta}_0} - \boldsymbol{\theta}_0\rvert$	$j=1$	$j=2$	$j=3$	$j=4$	$j=5$	$j=6$	$j=7$	$j=8$	$j=9$	$j=1$	$j=2$	$j=3$	$j=4$	$j=5$
A	0.005	0.146	0.016	0.003	0.002	0.013	0.004	0.023	0.002	0.015	0.002	0.005	0.003	0.087	0.007
C	0.004	0.025	0.039	0.002	0.024	0.009	0.03	0.188	0.026	0.012	0.016	0.076	0.003	0.029	0.023
D	0.005	0.006	0.004	0.011	0.025	0.025	0.024	0.007	0.029	0.019	0.006	0.019	0.016	0.004	0.031
E	0.007	0.019	0.016	0.034	0.003	0.009	0.004	0.027	0.062	0.057	0.062	0.012	0.012	0.067	0.021
F	0.016	0.095	0.11	0.055	0.005	0.017	0.003	0	0.025	0.002	0.05	0.031	0.029	0.045	0.054
G	0.001	0.013	0.047	0.011	0.01	0.013	0.062	0.02	0.026	0	0.032	0.043	0.011	0.107	0.001
H	0.019	0.005	0.001	0.014	0.005	0.03	0.018	0.002	0.004	0.002	0.028	0.018	0.004	0.026	0.009
I	0.002	0	0.002	0.001	0.016	0.001	0.01	0.027	0	0.022	0.003	0.02	0.002	0.002	0
K	0.011	0.006	0.005	0.007	0.035	0.017	0.205	0.001	0.073	0.008	0.008	0.08	0.006	0	0.013
L	0.008	0.02	0.004	0.121	0.036	0.017	0.003	0.003	0.012	0.017	0.23	0.037	0.014	0.013	0.004
M	0.004	0.012	0.027	0.049	0	0.013	0.011	0.011	0.002	0.011	0.011	0.01	0.001	0.028	0.032
N	0.002	0.004	0.034	0.005	0.042	0.005	0.006	0.004	0.003	0.002	0.006	0.035	0.012	0.001	0.007
P	0.001	0.006	0.003	0.001	0	0.087	0.013	0.006	0.014	0.002	0.01	0.017	0.044	0.025	0.002
Q	0.001	0.005	0.012	0.001	0.002	0.001	0.018	0.013	0.033	0.011	0.02	0.026	0.061	0.001	0.002
R	0.002	0.007	0.002	0.003	0.002	0.002	0.009	0.004	0.011	0.225	0.036	0.013	0.086	0.025	0.001
S	0.001	0.062	0.007	0.008	0.017	0.018	0.018	0.001	0.09	0.017	0	0.017	0.036	0.035	0.002
T	0.004	0.022	0.086	0.018	0.226	0	0.007	0.009	0.015	0.032	0.005	0	0.394	0.064	0.038
V	0.001	0.014	0.005	0.023	0.002	0.018	0.02	0.01	0.002	0.005	0.012	0.326	0.037	0.025	0.04
W	0.004	0.026	0.009	0.002	0.053	0.017	0.005	0.005	0.046	0.029	0.022	0.005	0.021	0.001	0.01
Y	0.004	0.002	0.005	0.058	0.017	0.261	0.016	0.03	0.028	0.021	0.052	0.013	0.076	0.028	0.051

$\boldsymbol{\gamma} = 0.2$

		$\lvert\widehat{\boldsymbol{\Theta}}_j - \boldsymbol{\Theta}_j\rvert$									$\lvert\widehat{\widetilde{\boldsymbol{\Theta}}}_j - \widetilde{\boldsymbol{\Theta}}_j\rvert$				
Letter	$\lvert\widehat{\boldsymbol{\theta}_0} - \boldsymbol{\theta}_0\rvert$	$j=1$	$j=2$	$j=3$	$j=4$	$j=5$	$j=6$	$j=7$	$j=8$	$j=9$	$j=1$	$j=2$	$j=3$	$j=4$	$j=5$
A	0.002	0.006	0.029	0.012	0.013	0.013	0.006	0.001	0.006	0.006	0.038	0.016	0.015	0.007	0.091
C	0.002	0.003	0.059	0.008	0.026	0.001	0.005	0.004	0.01	0.007	0.013	0.001	0.017	0.087	0
D	0.003	0.072	0.297	0.054	0	0.001	0.031	0.028	0.013	0.008	0.013	0.002	0.015	0.001	0.005
E	0.001	0.025	0.021	0.013	0.009	0	0.01	0.041	0.155	0.007	0.029	0.026	0.001	0.083	0.004
F	0.008	0.014	0.021	0.043	0.001	0.05	0.008	0.014	0.004	0.004	0.018	0.049	0.004	0.011	0.024
G	0.008	0.048	0.007	0.009	0.03	0.021	0.171	0.006	0.012	0.014	0.017	0.007	0.009	0.155	0.003
H	0.005	0.004	0.004	0.008	0.005	0.003	0.004	0.023	0.004	0.007	0.026	0.027	0.001	0.015	0.011
I	0.006	0.025	0.017	0	0.186	0.006	0.012	0.025	0.011	0.092	0.102	0.016	0.049	0.003	0.062
K	0.001	0.023	0.01	0.005	0.02	0.004	0.03	0.004	0.023	0.002	0.002	0.206	0.055	0.006	0.033
L	0.005	0.002	0.003	0.006	0.002	0.009	0.012	0.009	0	0.081	0.01	0	0.003	0.019	0.013
M	0.001	0.006	0.005	0.017	0.026	0.009	0.01	0.002	0.046	0.01	0.076	0.001	0.015	0.001	0.005
N	0.001	0.001	0.02	0.018	0.024	0.013	0.003	0.063	0.006	0.03	0.012	0.011	0.022	0.004	0.003
P	0.003	0.005	0.003	0.007	0.025	0.256	0.006	0.016	0.004	0.04	0.019	0.001	0.058	0.014	0.031
Q	0	0.006	0.006	0.005	0.01	0.024	0.018	0.007	0.006	0.011	0.008	0.039	0.022	0.001	0.019
R	0.003	0.038	0.051	0.011	0.016	0.017	0.007	0.005	0.032	0.01	0.003	0.022	0.011	0.012	0.002
S	0	0.066	0.022	0.003	0.005	0.047	0.011	0.032	0.022	0.006	0.103	0.002	0.079	0.003	0.004
T	0.003	0.003	0.003	0.134	0.014	0.005	0.015	0.001	0.041	0.004	0.001	0.009	0.002	0.023	0.061
V	0.002	0.013	0.004	0.002	0.04	0.015	0.006	0.003	0.008	0.077	0.021	0.001	0.098	0.002	0.015
W	0	0.126	0.021	0.024	0.01	0.013	0.016	0.051	0.026	0.006	0.023	0.005	0.004	0.013	0.031
Y	0.001	0.014	0.003	0.002	0.005	0.007	0.021	0.179	0.001	0.004	0.006	0.029	0.002	0.022	0.015

Table 8: $\lambda = 1$ and $\boldsymbol{\eta} = 0.1$

$\boldsymbol{\gamma} = 0.05$

Letter	$\lvert\widehat{\boldsymbol{\theta}_0} - \boldsymbol{\theta}_0\rvert$	$\lvert\widehat{\boldsymbol{\Theta}}_j - \boldsymbol{\Theta}_j\rvert$ $j=1$	$j=2$	$j=3$	$j=4$	$j=5$	$j=6$	$j=7$	$j=8$	$j=9$	$\lvert\widehat{\widetilde{\boldsymbol{\Theta}}}_j - \widetilde{\boldsymbol{\Theta}}_j\rvert$ $j=1$	$j=2$	$j=3$	$j=4$	$j=5$
A	0.004	0.016	0.205	0.014	0.001	0.009	0.056	0.007	0	0.017	0.002	0.085	0.026	0.008	0.002
C	0.005	0.029	0.023	0.109	0.011	0.002	0.036	0.039	0.001	0.05	0.002	0.004	0.025	0.009	0.055
D	0	0	0.026	0.077	0.006	0.072	0.005	0.048	0.002	0.01	0.008	0.004	0.006	0.014	0.035
E	0.013	0.006	0.016	0.01	0.011	0.06	0.004	0.014	0.012	0.004	0.006	0.007	0.01	0.006	0.003
F	0.003	0.028	0	0.021	0.026	0.037	0.047	0.019	0.109	0.025	0.017	0.021	0.004	0.056	0.312
G	0.002	0.051	0.021	0	0.018	0.011	0	0.038	0.011	0.021	0.018	0.01	0.03	0.039	0.005
H	0.003	0.012	0.004	0.005	0.026	0.001	0.003	0.004	0.038	0.016	0.281	0.066	0.015	0.012	0.01
I	0.003	0.062	0.004	0.041	0.005	0.003	0.012	0.081	0.009	0.002	0.007	0.007	0.003	0.021	0.037
K	0.002	0.007	0.026	0.04	0.013	0.003	0.075	0.018	0.008	0.019	0.018	0.018	0.007	0.003	0.046
L	0.005	0.02	0.025	0.007	0.021	0.001	0.009	0.04	0.04	0.041	0	0.34	0.106	0.155	0.014
M	0.005	0.097	0.023	0.009	0.006	0.004	0.011	0.02	0.014	0.001	0.014	0.004	0.015	0.006	0.006
N	0.002	0.129	0.054	0.05	0.009	0.007	0.021	0.005	0.02	0.001	0.001	0.003	0.01	0.01	0.012
P	0.001	0.019	0.03	0.005	0.049	0.034	0.02	0.017	0.008	0.049	0.022	0.004	0.007	0.045	0.011
Q	0.014	0.006	0.008	0.009	0.208	0.02	0.035	0.008	0.019	0.005	0.024	0.062	0.012	0.004	0.095
R	0.001	0.027	0.008	0.012	0.008	0.075	0.003	0.214	0.01	0.013	0.003	0.019	0.031	0.017	0.041
S	0	0.006	0.013	0.013	0.012	0.027	0.014	0.031	0.016	0.135	0.019	0.035	0.029	0.002	0.011
T	0.002	0.036	0.015	0.009	0.008	0.083	0.023	0.037	0.026	0.006	0.031	0.028	0.284	0.058	0.002
V	0.001	0	0.016	0.002	0.003	0.083	0.013	0.013	0.026	0.082	0.011	0.021	0.025	0.009	0.017
W	0.016	0.037	0.004	0.001	0.002	0.013	0.011	0.006	0.007	0.051	0.078	0.013	0.022	0.022	0.012
Y	0.001	0.014	0.004	0.022	0.014	0.018	0	0.002	0.012	0.03	0.04	0.072	0.01	0.011	0.015

$\boldsymbol{\gamma} = 0.1$

Letter	$\lvert\widehat{\boldsymbol{\theta}_0} - \boldsymbol{\theta}_0\rvert$	$\lvert\widehat{\boldsymbol{\Theta}}_j - \boldsymbol{\Theta}_j\rvert$ $j=1$	$j=2$	$j=3$	$j=4$	$j=5$	$j=6$	$j=7$	$j=8$	$j=9$	$\lvert\widehat{\widetilde{\boldsymbol{\Theta}}}_j - \widetilde{\boldsymbol{\Theta}}_j\rvert$ $j=1$	$j=2$	$j=3$	$j=4$	$j=5$
A	0	0.036	0.028	0.01	0.002	0.055	0.009	0.031	0.073	0.033	0.005	0.009	0.022	0.002	0.021
C	0.006	0.021	0.003	0.027	0.011	0.005	0.001	0.059	0.013	0.015	0.007	0.022	0.027	0.014	0.005
D	0	0.005	0.001	0	0.063	0.063	0.002	0.007	0.002	0.029	0.05	0.007	0.012	0.039	0.027
E	0.009	0.035	0.02	0.057	0.013	0.003	0.006	0.046	0.031	0.021	0.046	0.019	0.032	0.016	0.25
F	0.009	0.002	0.028	0.011	0.008	0.126	0.001	0.003	0.035	0.005	0.001	0.119	0.174	0.015	0.017
G	0.005	0.024	0.002	0.033	0.02	0.022	0.007	0.001	0.001	0.006	0.014	0.013	0	0.013	0.009
H	0.003	0.064	0.006	0.001	0.047	0.03	0.014	0.023	0.01	0.002	0.028	0.02	0.005	0.055	0
I	0.002	0.004	0.006	0.186	0.017	0.009	0.004	0.043	0.033	0.046	0.014	0.04	0	0.028	0.008
K	0.002	0.009	0.014	0.02	0	0.004	0	0.025	0.001	0.003	0.005	0.012	0.029	0.037	0.079
L	0.001	0.011	0.004	0.007	0.034	0.004	0.001	0.008	0.021	0.001	0.003	0.02	0.035	0.034	0.031
M	0.005	0.014	0.01	0.011	0.021	0.117	0.009	0.005	0.01	0.061	0.018	0.277	0.038	0.018	0.119
N	0.007	0.074	0.001	0.033	0.001	0.009	0.015	0.004	0.015	0.033	0.079	0.015	0.004	0.026	0.004
P	0.004	0.009	0.005	0.006	0.008	0.025	0.005	0.007	0.03	0.023	0.029	0.005	0.016	0.003	0.017
Q	0.004	0.034	0.014	0.016	0.001	0.005	0.012	0.089	0.036	0.023	0.039	0.005	0.001	0.004	0.022
R	0.001	0.011	0.015	0.024	0.01	0.009	0.088	0.021	0.013	0.125	0.123	0.113	0.026	0.035	0.004
S	0.002	0.006	0.048	0.028	0.065	0	0.056	0.009	0.004	0.012	0.001	0.035	0.002	0.025	0.005
T	0.008	0.006	0.026	0.051	0.01	0.021	0.151	0.035	0.1	0.008	0.073	0.015	0.02	0.228	0.066
V	0.002	0.126	0.116	0.005	0.087	0.008	0.067	0.008	0.018	0.01	0.032	0.03	0.003	0.01	0.008
W	0	0.005	0.002	0.017	0.048	0.001	0.037	0.203	0.013	0.004	0.021	0.002	0.042	0.053	0.054
Y	0.001	0.005	0.013	0.006	0	0.031	0.03	0.01	0.166	0.002	0.005	0.015	0.005	0.016	0.02

$\boldsymbol{\gamma} = 0.2$

Letter	$\lvert\widehat{\boldsymbol{\theta}_0} - \boldsymbol{\theta}_0\rvert$	$\lvert\widehat{\boldsymbol{\Theta}}_j - \boldsymbol{\Theta}_j\rvert$ $j=1$	$j=2$	$j=3$	$j=4$	$j=5$	$j=6$	$j=7$	$j=8$	$j=9$	$\lvert\widehat{\widetilde{\boldsymbol{\Theta}}}_j - \widetilde{\boldsymbol{\Theta}}_j\rvert$ $j=1$	$j=2$	$j=3$	$j=4$	$j=5$
A	0.002	0.069	0.011	0.009	0.09	0.011	0.014	0.005	0	0.001	0.111	0.044	0.117	0.01	0.158
C	0.008	0.073	0.011	0	0.005	0.012	0.002	0.002	0.011	0.002	0.054	0.086	0.022	0.09	0.002
D	0.003	0.007	0.051	0.029	0.001	0.047	0.004	0.008	0.009	0.003	0.013	0.006	0.1	0.301	0.008
E	0.004	0.018	0.049	0.041	0.028	0	0.005	0.002	0.001	0.008	0.033	0.037	0.181	0.034	0.001
F	0.005	0.004	0.011	0.014	0.095	0.001	0.022	0.002	0.064	0.021	0	0.079	0.032	0.024	0.044
G	0.001	0.01	0.016	0.001	0.011	0.014	0.001	0.051	0.016	0.013	0.005	0.041	0.023	0.026	0.053
H	0.007	0.025	0.013	0.017	0.001	0.016	0.051	0.038	0.036	0.012	0.006	0.027	0.034	0.007	0.055
I	0.002	0.025	0.006	0.029	0	0.008	0.025	0.013	0.009	0.012	0.007	0.131	0.006	0.046	0.012
K	0.002	0.011	0.09	0.005	0.003	0.095	0.003	0.04	0.036	0.008	0.265	0.061	0.065	0.06	0.001
L	0.004	0.012	0.005	0.005	0.006	0.017	0.003	0.007	0.002	0.018	0.033	0.031	0.005	0.026	0.002
M	0.009	0.073	0.006	0.022	0.018	0.011	0.104	0.005	0.009	0.001	0.027	0.03	0.005	0.009	0.006
N	0.001	0.018	0.014	0.024	0.001	0.019	0.048	0.006	0.018	0.042	0.013	0.015	0.016	0.003	0.001
P	0.002	0.014	0.031	0.021	0.018	0.065	0.012	0.004	0.045	0.233	0.029	0.031	0.007	0	0.003
Q	0.008	0.029	0.011	0.023	0.011	0.013	0.092	0.043	0.001	0.052	0.006	0.091	0.173	0.035	0.001
R	0.002	0.009	0.041	0.022	0.042	0.001	0.001	0.001	0.284	0.004	0.034	0.002	0.002	0.019	0.062
S	0.007	0.019	0.01	0.005	0.012	0.025	0.003	0.005	0.006	0.003	0.077	0.027	0.008	0.004	0.054
T	0.011	0.017	0.109	0.007	0.007	0.022	0.003	0.002	0.012	0.002	0.014	0.017	0.117	0.033	0.054
V	0.008	0.104	0.001	0.009	0.005	0.015	0.027	0.006	0.005	0.014	0.026	0.007	0.031	0.024	0.011
W	0.011	0.004	0.006	0.001	0.012	0.015	0.001	0.024	0	0.018	0.09	0.016	0.013	0.108	0.063
Y	0.003	0.017	0.018	0.159	0.029	0.247	0.016	0.005	0.003	0.041	0.002	0.001	0.013	0.017	0.006

Table 9: $\lambda = 1$ and $\boldsymbol{\eta} = 0.2$

$\boldsymbol{\gamma} = 0.05$

		$\lvert\widehat{\boldsymbol{\Theta}}_j - \boldsymbol{\Theta}_j\rvert$									$\lvert\widehat{\widetilde{\boldsymbol{\Theta}}}_j - \widetilde{\boldsymbol{\Theta}}_j\rvert$				
Letter	$\lvert\widehat{\boldsymbol{\theta}_0} - \boldsymbol{\theta}_0\rvert$	$j=1$	$j=2$	$j=3$	$j=4$	$j=5$	$j=6$	$j=7$	$j=8$	$j=9$	$j=1$	$j=2$	$j=3$	$j=4$	$j=5$
A	0.005	0.046	0.009	0.001	0.035	0.102	0.009	0.005	0.012	0.006	0.01	0.101	0.01	0.015	0.001
C	0.003	0.002	0.003	0.001	0.018	0.003	0.008	0.014	0.06	0.008	0.001	0.03	0	0.002	0.025
D	0.001	0.023	0.069	0.038	0.001	0.016	0.002	0.008	0.002	0.008	0.003	0.011	0.004	0.003	0.017
E	0.003	0.005	0.006	0.001	0.022	0.003	0.005	0.002	0.013	0.053	0.021	0.003	0.011	0.01	0.01
F	0.007	0	0.03	0.038	0	0.021	0.012	0.024	0.034	0.004	0.167	0.023	0.034	0.01	0.018
G	0.002	0.036	0.034	0.004	0.025	0.002	0.004	0.009	0.034	0.002	0.006	0.001	0.037	0.004	0.07
H	0.014	0.005	0.024	0.028	0.007	0.008	0.011	0.006	0.099	0.005	0.002	0.006	0.002	0.003	0.02
I	0.001	0.011	0.033	0.001	0.005	0.004	0.169	0.098	0.033	0.037	0.014	0.017	0.001	0.005	0.025
K	0.001	0.009	0.074	0.036	0.002	0.025	0.026	0.013	0.027	0.001	0.03	0.022	0.007	0.053	0.037
L	0.011	0.033	0.035	0.028	0.015	0.002	0.003	0.035	0.004	0.001	0.013	0	0.006	0.075	0.023
M	0.008	0.032	0.025	0.028	0.007	0.024	0.029	0.004	0.007	0.004	0.001	0.011	0.006	0.165	0.006
N	0.001	0.17	0.118	0.009	0.03	0.007	0.016	0.037	0.097	0.017	0.025	0.008	0.011	0.004	0.004
P	0.012	0.003	0.026	0.028	0.002	0.017	0.048	0.029	0	0.074	0.004	0.088	0.023	0.068	0.007
Q	0.005	0.016	0.009	0.003	0.025	0.021	0.05	0	0.015	0.015	0.031	0.008	0.017	0.024	0.006
R	0.003	0.002	0.027	0.017	0.019	0.009	0.003	0.007	0.001	0.149	0.003	0.017	0.013	0.008	0.002
S	0.001	0.028	0.014	0.162	0.06	0.035	0.048	0.002	0.031	0.035	0.016	0.002	0.008	0.008	0.006
T	0.005	0.021	0.005	0.024	0.123	0.011	0.011	0.022	0.07	0.012	0.009	0.014	0.002	0.006	0.003
V	0.013	0.059	0.033	0.013	0.032	0.02	0.006	0.03	0.002	0.006	0.031	0.015	0	0.015	0.006
W	0.001	0.002	0.026	0.03	0.017	0.002	0	0.017	0.004	0.006	0.001	0	0.019	0.001	0.012
Y	0.003	0.008	0.005	0.03	0.013	0.009	0.031	0.074	0.002	0.041	0.017	0.001	0.139	0.004	0.143

$\boldsymbol{\gamma} = 0.1$

		$\lvert\widehat{\boldsymbol{\Theta}}_j - \boldsymbol{\Theta}_j\rvert$									$\lvert\widehat{\widetilde{\boldsymbol{\Theta}}}_j - \widetilde{\boldsymbol{\Theta}}_j\rvert$				
Letter	$\lvert\widehat{\boldsymbol{\theta}_0} - \boldsymbol{\theta}_0\rvert$	$j=1$	$j=2$	$j=3$	$j=4$	$j=5$	$j=6$	$j=7$	$j=8$	$j=9$	$j=1$	$j=2$	$j=3$	$j=4$	$j=5$
A	0	0.014	0.051	0.016	0.027	0.01	0.012	0.025	0.093	0.002	0.017	0.024	0.007	0.005	0.01
C	0.005	0.018	0.008	0.021	0.013	0.048	0.017	0.004	0.023	0.055	0.045	0.001	0.002	0.012	0.007
D	0.004	0.018	0.014	0.003	0.032	0.011	0.012	0.017	0.039	0.013	0.182	0.003	0.001	0.007	0.001
E	0.001	0.018	0.004	0.014	0.062	0.03	0.024	0.005	0.01	0.024	0.013	0.007	0.028	0.017	0.016
F	0.008	0.003	0.045	0.028	0.012	0.006	0.001	0.002	0.021	0.007	0.048	0.108	0.054	0.014	0.018
G	0.006	0.033	0.079	0.006	0.052	0.018	0.018	0.008	0.001	0.016	0.019	0.021	0.005	0.094	0.009
H	0.019	0.041	0.005	0.012	0.011	0.033	0.163	0.011	0.002	0.041	0.066	0.021	0.015	0.006	0.031
I	0.003	0.05	0.083	0.049	0.019	0.008	0.001	0.001	0.099	0.016	0.007	0.02	0.056	0.013	0.043
K	0	0.005	0.006	0.007	0.001	0.093	0.016	0.011	0.015	0.068	0.013	0.016	0.011	0.011	0.009
L	0.001	0.009	0.008	0.053	0.006	0.027	0.036	0.086	0.021	0	0.002	0.157	0.027	0.011	0.001
M	0.001	0.042	0.004	0.096	0.032	0.014	0.008	0.001	0.005	0.036	0.011	0.011	0.024	0.002	0.055
N	0.001	0.009	0.011	0.002	0.08	0.014	0	0.033	0.003	0.008	0.002	0.018	0.012	0.006	0.018
P	0.004	0.031	0.001	0.024	0.005	0.041	0.003	0.024	0.019	0.04	0.006	0.005	0.063	0.02	0.119
Q	0.002	0.012	0.028	0.021	0.001	0.004	0.011	0.033	0.063	0.096	0.024	0.003	0.005	0.009	0.058
R	0.001	0.011	0.047	0.106	0.264	0.05	0.001	0.014	0.014	0.049	0.037	0.022	0.001	0.015	0.024
S	0.005	0.012	0.081	0.062	0.025	0.017	0.002	0.086	0.03	0.015	0.036	0.002	0.034	0.012	0.037
T	0.008	0.002	0.035	0.018	0.022	0.003	0.03	0.027	0.002	0.01	0.007	0.009	0.003	0.017	0.002
V	0	0.063	0.001	0.056	0.001	0.023	0.011	0.007	0.006	0.002	0.014	0.064	0.018	0.002	0.004
W	0.001	0.001	0.006	0.016	0.019	0.022	0.018	0.045	0.029	0.001	0.001	0.016	0.047	0.004	0.01
Y	0.005	0.01	0.009	0.016	0.002	0.001	0.021	0.004	0.137	0.019	0.004	0.004	0.005	0.018	0

$\boldsymbol{\gamma} = 0.2$

		$\lvert\widehat{\boldsymbol{\Theta}}_j - \boldsymbol{\Theta}_j\rvert$									$\lvert\widehat{\widetilde{\boldsymbol{\Theta}}}_j - \widetilde{\boldsymbol{\Theta}}_j\rvert$				
Letter	$\lvert\widehat{\boldsymbol{\theta}_0} - \boldsymbol{\theta}_0\rvert$	$j=1$	$j=2$	$j=3$	$j=4$	$j=5$	$j=6$	$j=7$	$j=8$	$j=9$	$j=1$	$j=2$	$j=3$	$j=4$	$j=5$
A	0.001	0.007	0.022	0.023	0.039	0.002	0.003	0.016	0.008	0.002	0.004	0.005	0	0.005	0.031
C	0	0.03	0.096	0.001	0.01	0.043	0.038	0.003	0.003	0.015	0	0.023	0.001	0.033	0.006
D	0.001	0.088	0.026	0.057	0.001	0.004	0.007	0.004	0.007	0.065	0.005	0.051	0.006	0.01	0.009
E	0.003	0.051	0.004	0.002	0.011	0.064	0.018	0.021	0.052	0.021	0.014	0.072	0.034	0.012	0.011
F	0	0.059	0.001	0.013	0.002	0.042	0.043	0.055	0.022	0.024	0.034	0.009	0.012	0.029	0.006
G	0.003	0.003	0.049	0.21	0.021	0.001	0.014	0.042	0.049	0.002	0.022	0.008	0.007	0.009	0.026
H	0.007	0.032	0.015	0.037	0.026	0	0.036	0.005	0.006	0.009	0.005	0.021	0.002	0.007	0.05
I	0	0.017	0.103	0.004	0.012	0.039	0.017	0.15	0.053	0.032	0.011	0.004	0.09	0.02	0.015
K	0	0.007	0.069	0.002	0.069	0.036	0.021	0.073	0.007	0.026	0.007	0.005	0.011	0.008	0.035
L	0.003	0.025	0.011	0.044	0.005	0.054	0.01	0	0.037	0.02	0.032	0.007	0.029	0.013	0.007
M	0.002	0.14	0.015	0.037	0.015	0.008	0.113	0.054	0.316	0.008	0.052	0.011	0.048	0.036	0.002
N	0.005	0.046	0.01	0.036	0.017	0.027	0.003	0	0.008	0.005	0.032	0.123	0.005	0.018	0.024
P	0.001	0.027	0.001	0.013	0.022	0.015	0.001	0.056	0.002	0.02	0.025	0.01	0.011	0.001	0.059
Q	0.013	0.002	0.044	0.022	0.139	0.084	0.001	0.012	0.037	0.003	0.015	0.051	0.088	0.007	0.026
R	0.002	0.013	0.027	0.013	0.034	0.004	0.021	0.041	0.03	0.026	0.003	0.008	0.008	0.004	0.015
S	0.004	0.101	0.02	0.001	0.005	0.058	0.01	0.021	0.034	0.015	0.047	0.012	0.034	0.01	0.021
T	0.005	0.005	0	0.003	0.012	0.024	0.018	0.008	0.034	0.047	0.007	0.004	0.024	0.025	0
V	0.007	0.009	0.111	0.031	0.009	0.014	0.001	0.02	0.004	0.055	0.002	0.01	0.007	0.002	0.023
W	0.005	0.001	0	0.035	0	0.009	0.02	0.124	0.014	0.008	0.076	0.013	0.011	0.05	0.008
Y	0.002	0.004	0.004	0.015	0.022	0.039	0.026	0.002	0.033	0.018	0.006	0.005	0.05	0.006	0.001

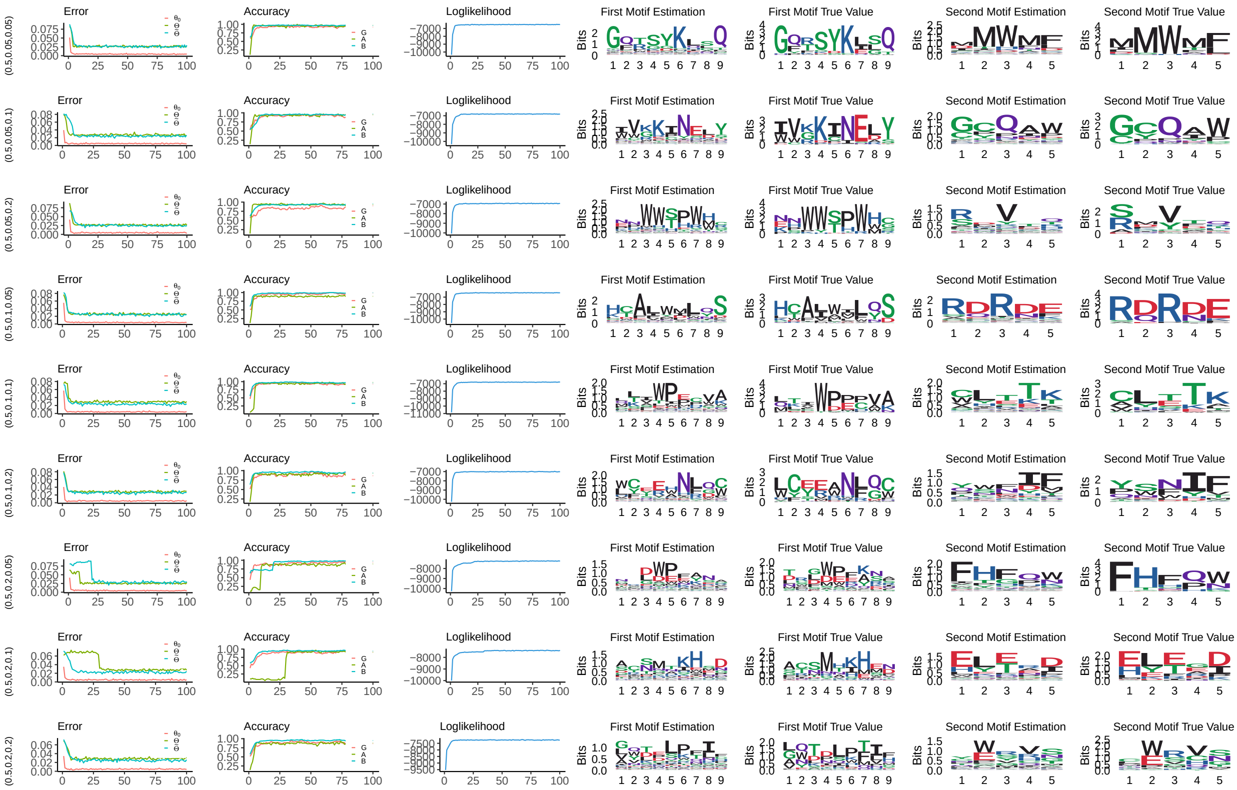

Figure 2: Results for the case of $\lambda = 0.5$.

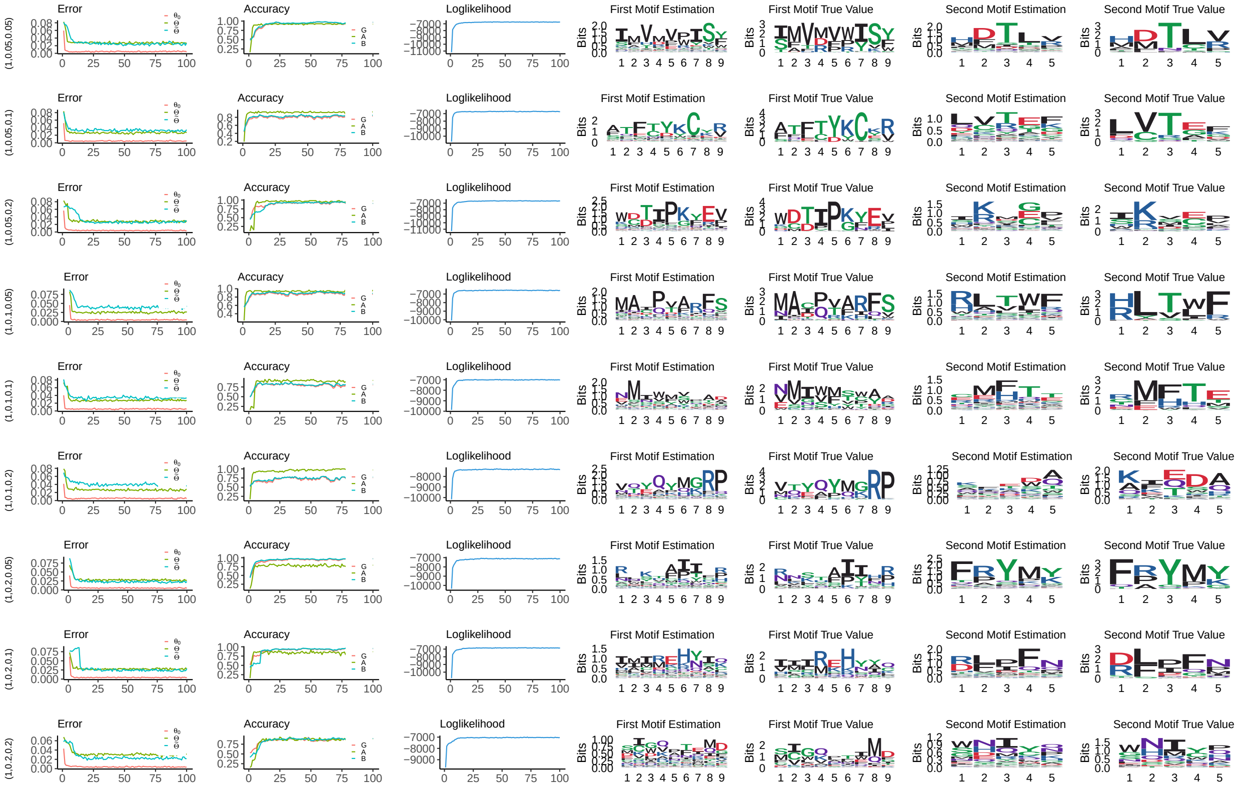

Figure 3: Results for the case of $\lambda = 1$.

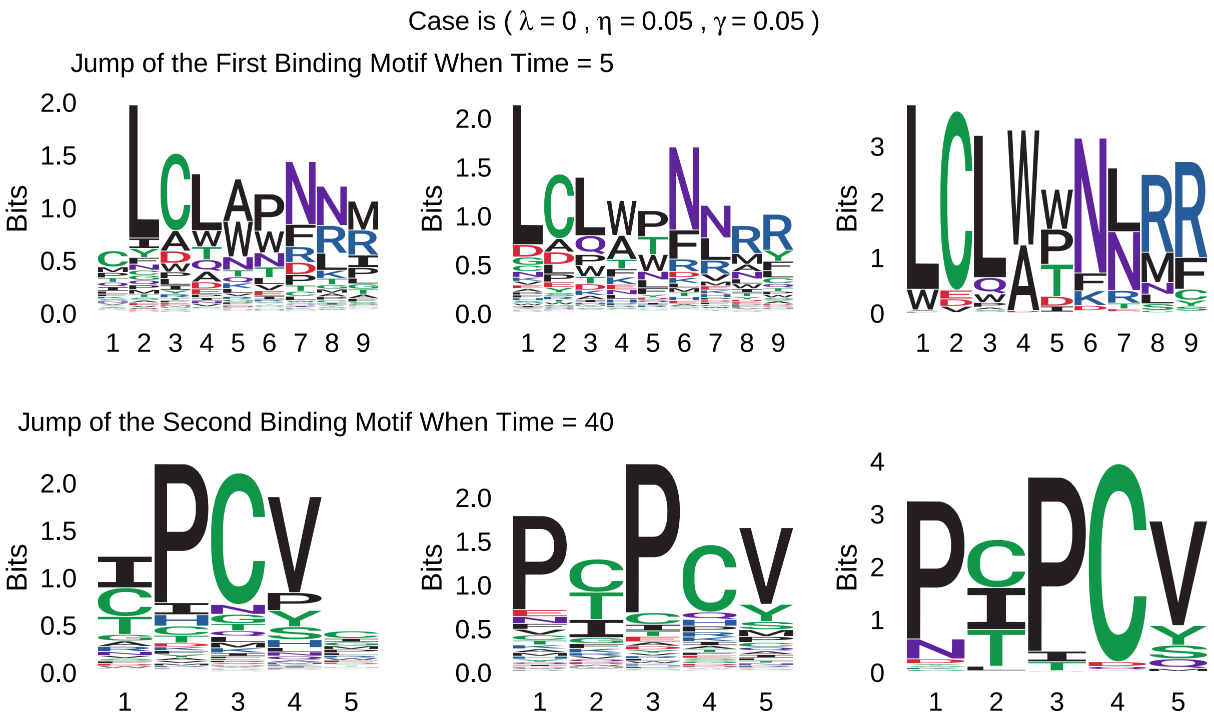

Figure 4: Jump for the case of $\lambda = 0$, $\boldsymbol{\eta} = 0.05$ and $\boldsymbol{\gamma} = 0.05$.

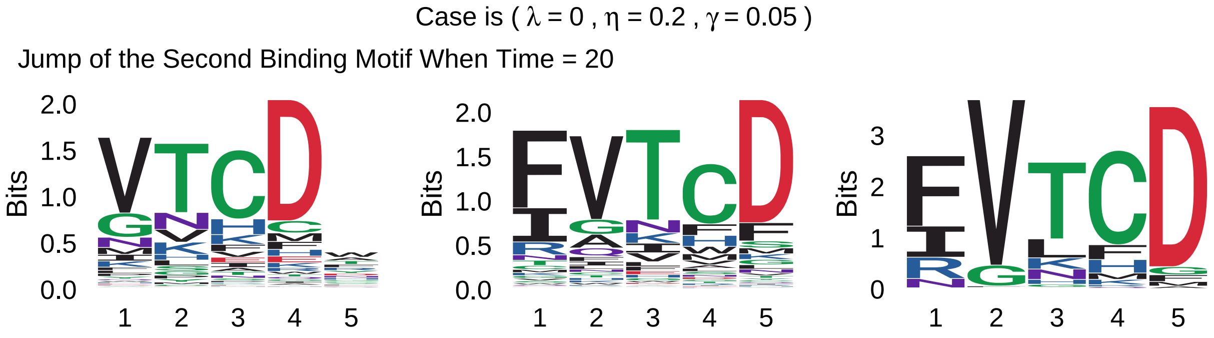

Figure 5: Jump for the case of $\lambda = 0$, $\boldsymbol{\eta} = 0.2$ and $\boldsymbol{\gamma} = 0.05$.

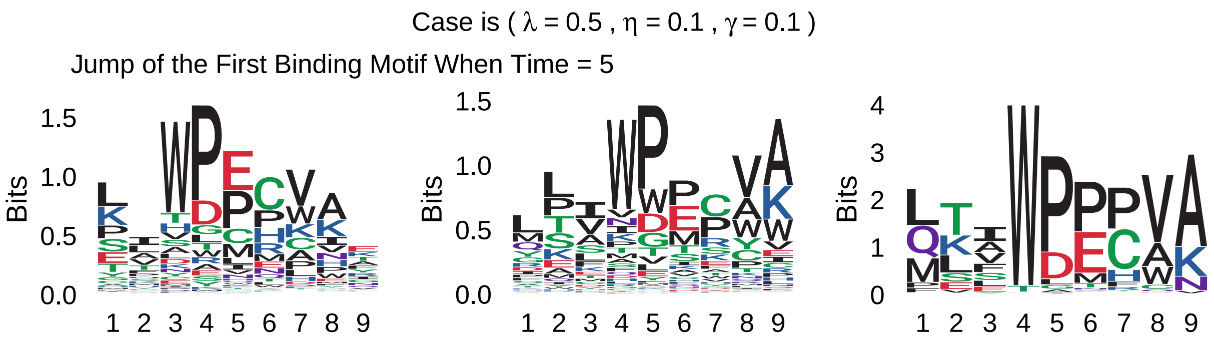

Figure 6: Jump for the case of $\lambda = 0.5$, $\boldsymbol{\eta} = 0.1$ and $\boldsymbol{\gamma} = 0.1$.

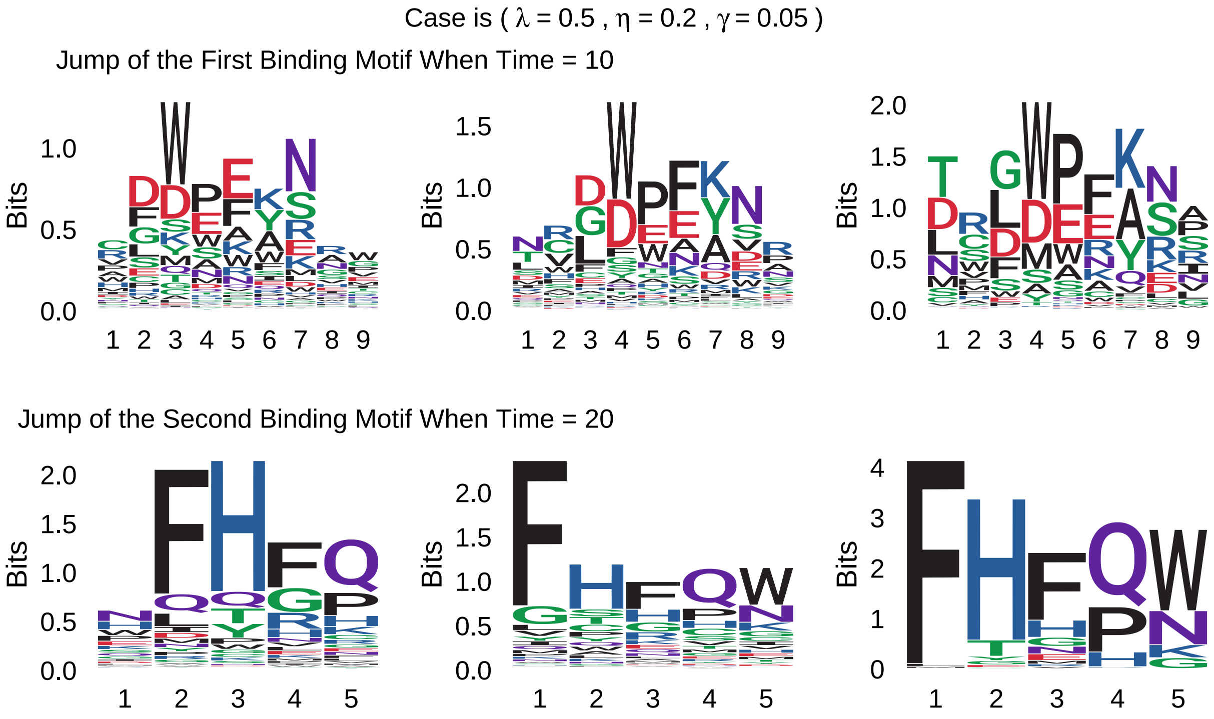

Figure 7: Jump for the case of $\lambda = 0.5$, $\boldsymbol{\eta} = 0.2$ and $\boldsymbol{\gamma} = 0.05$.

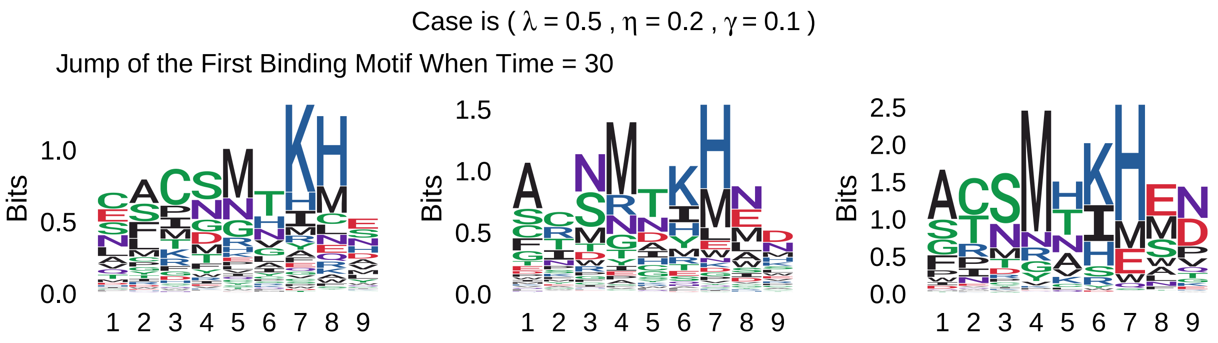

Figure 8: Jump for the case of $\lambda = 0.5$, $\boldsymbol{\eta} = 0.2$ and $\boldsymbol{\gamma} = 0.1$.

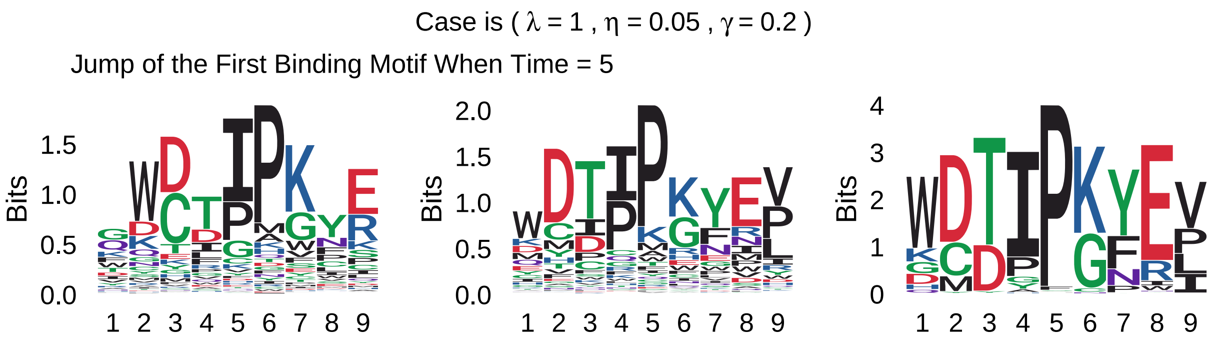

Figure 9: Jump for the case of $\lambda = 1$, $\boldsymbol{\eta} = 0.05$ and $\boldsymbol{\gamma} = 0.2$.

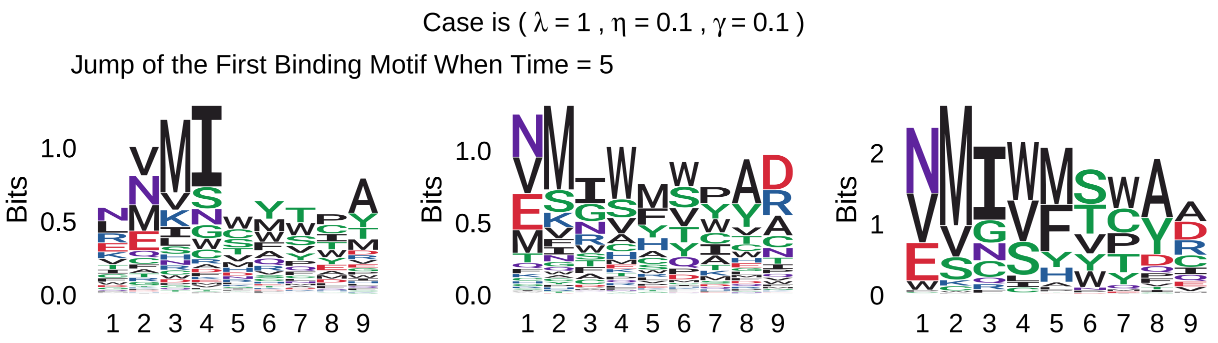

Figure 10: Jump for the case of $\lambda = 1$, $\boldsymbol{\eta} = 0.1$ and $\boldsymbol{\gamma} = 0.1$.

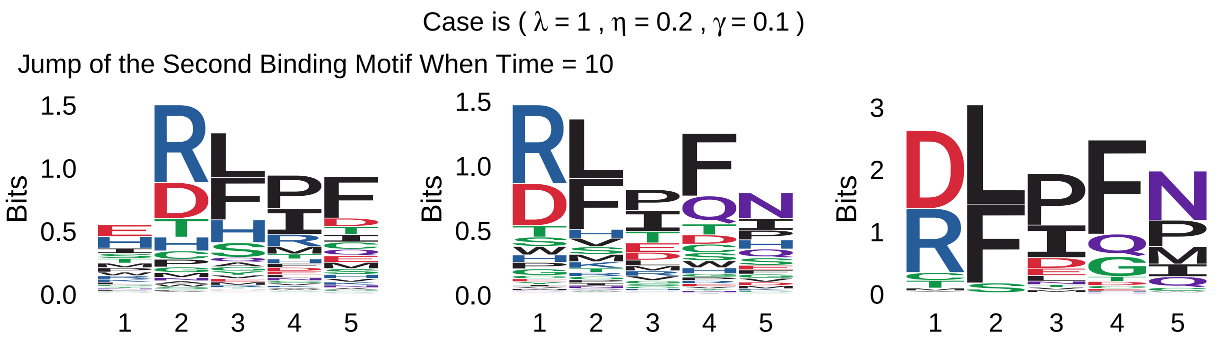

Figure 11: Jump for the case of $\lambda = 1$, $\boldsymbol{\eta} = 0.2$ and $\boldsymbol{\gamma} = 0.1$.

2. Performance under Different Number of Sequences and Sequence Length

The absolute errors for $\widehat{\boldsymbol{\theta}}_0$, $\widehat{\boldsymbol{\Theta}}$, and $\widehat{\widehat{\boldsymbol{\Theta}}}$ in the case of $\lambda = 1$, $\boldsymbol{\eta} = 0.05$, and $\boldsymbol{\gamma} = 0.05$ under different numbers of sequences and sequence lengths are presented in Tables 10 through 12. Figure 12 illustrates the mean error curves for parameters $\boldsymbol{\theta}_0$, $\boldsymbol{\Theta}$, and $\widetilde{\boldsymbol{\Theta}}$ across different sequence numbers and lengths. It also shows accuracy curves for latent variables $\boldsymbol{G}$, $\boldsymbol{A}$, and $\boldsymbol{B}$, the likelihood curve, and the estimated vs. true values for both $\boldsymbol{\Theta}$ and $\widetilde{\boldsymbol{\Theta}}$. Figures 13 to 15 depict the jumps for the first and second motifs across different sequence numbers and lengths.

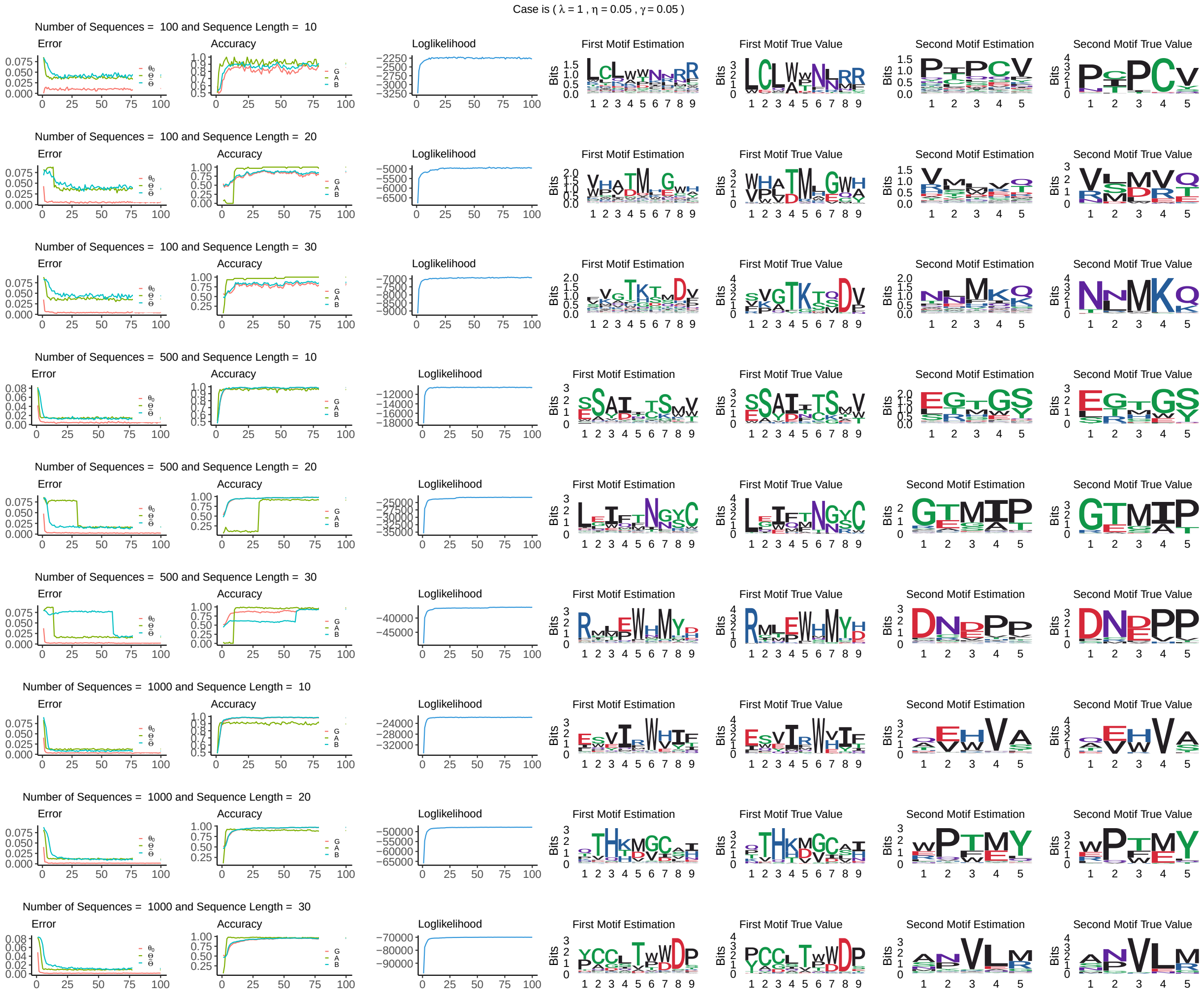

Figure 12: Results for the case of $\lambda = 1$, $\eta = 0.05$ and $\gamma = 0.05$.

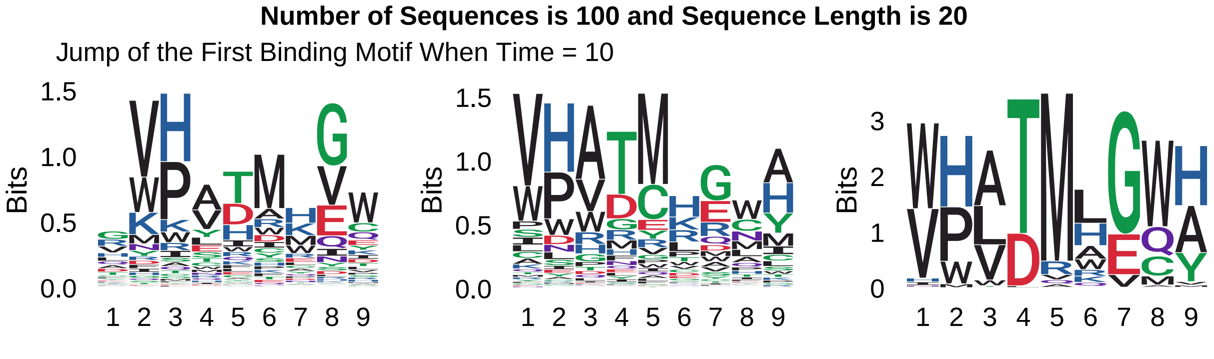

Figure 13: Jump for the case of the number of sequences is 100 and sequence length is 20.

Table 10: Number of Sequences is 100

Sequence Length is 10															
		$\lvert\widehat{\boldsymbol{\Theta}}_j - \boldsymbol{\Theta}_j\rvert$									$\lvert\widehat{\widetilde{\boldsymbol{\Theta}}}_j - \widetilde{\boldsymbol{\Theta}}_j\rvert$				
Letter	$\lvert\widehat{\boldsymbol{\theta}_0} - \boldsymbol{\theta}_0\rvert$	$j=1$	$j=2$	$j=3$	$j=4$	$j=5$	$j=6$	$j=7$	$j=8$	$j=9$	$j=1$	$j=2$	$j=3$	$j=4$	$j=5$
A	0.009	0.004	0.009	0.004	0.135	0.118	0.034	0.022	0.025	0	0.016	0.041	0.028	0.008	0.051
C	0	0.017	0.216	0.005	0.003	0.035	0.007	0.009	0.056	0.036	0.228	0.359	0.572	0.933	0.022
D	0.002	0.007	0.031	0.023	0.033	0.003	0.006	0.012	0.001	0.014	0.001	0.057	0.009	0.02	0.088
E	0.008	0.013	0.025	0.027	0	0.019	0.026	0.006	0.014	0.014	0.055	0.052	0.037	0.007	0.033
F	0.007	0.003	0.043	0.085	0.003	0.003	0.054	0.041	0.002	0.049	0.04	0.068	0.013	0.084	0.017
G	0.006	0.004	0.007	0.015	0.027	0.011	0.038	0.01	0.046	0.002	0.009	0.029	0.02	0.098	0.029
H	0.002	0.004	0.005	0.003	0.005	0.011	0.009	0.023	0.003	0.006	0.01	0.001	0.045	0.026	0.075
I	0.002	0.042	0.005	0.021	0.014	0.005	0.001	0.022	0.013	0.01	0.14	0.258	0.01	0.002	0.009
K	0.018	0.01	0.016	0.027	0.007	0.01	0.062	0.005	0.026	0.047	0.034	0.004	0.015	0.028	0.055
L	0.023	0.2	0.038	0.364	0.019	0.052	0.001	0.216	0.041	0.013	0.029	0.023	0.029	0	0.087
M	0.01	0.006	0.003	0.019	0.009	0.071	0.009	0.022	0.076	0.008	0.01	0	0.006	0.008	0.037
N	0	0.037	0.072	0.008	0.007	0.006	0.362	0.083	0.043	0.058	0.106	0.006	0.005	0.001	0.025
P	0.016	0.057	0.032	0.031	0.002	0.151	0.038	0.024	0.015	0.004	0.76	0.584	0.881	0.034	0.131
Q	0.015	0.005	0.027	0.011	0.042	0.036	0.059	0.011	0.01	0.04	0.001	0.001	0.02	0.041	0.063
R	0.013	0.007	0.01	0.034	0.01	0.011	0.023	0.006	0.186	0.171	0.017	0.006	0.029	0.017	0.069
S	0.008	0.012	0.002	0.007	0.049	0.007	0.019	0.002	0.045	0.003	0.031	0.004	0.001	0.024	0.026
T	0.004	0.004	0.001	0.02	0.016	0.08	0.025	0.071	0.009	0.026	0.217	0.261	0.021	0.014	0.015
V	0	0.029	0.021	0.03	0.001	0.017	0.027	0.03	0.024	0.01	0.025	0.008	0.016	0.443	0.617
W	0.01	0.046	0.02	0.013	0.121	0.169	0.023	0.001	0.014	0.015	0.009	0.02	0.019	0.002	0.021
Y	0.016	0.001	0.006	0.026	0.006	0	0.021	0.006	0.052	0.014	0.013	0.026	0.005	0.12	0.059
Sequence Length is 20															
		$\lvert\widehat{\boldsymbol{\Theta}}_j - \boldsymbol{\Theta}_j\rvert$									$\lvert\widehat{\widetilde{\boldsymbol{\Theta}}}_j - \widetilde{\boldsymbol{\Theta}}_j\rvert$				
Letter	$\lvert\widehat{\boldsymbol{\theta}_0} - \boldsymbol{\theta}_0\rvert$	$j=1$	$j=2$	$j=3$	$j=4$	$j=5$	$j=6$	$j=7$	$j=8$	$j=9$	$j=1$	$j=2$	$j=3$	$j=4$	$j=5$
A	0	0.007	0.057	0.001	0.014	0.006	0.077	0	0.028	0.158	0.042	0.035	0.017	0.02	0.039
C	0.005	0.003	0.01	0.007	0.045	0.005	0.017	0.003	0.012	0.003	0.002	0.033	0.01	0.056	0.001
D	0.007	0.014	0.003	0.021	0.056	0.055	0.029	0.015	0.005	0.001	0.071	0.029	0.204	0.081	0.084
E	0.005	0.001	0.013	0.012	0.012	0.005	0.008	0.038	0.047	0.024	0.017	0.004	0.001	0.021	0.139
F	0.005	0.047	0.005	0.032	0.003	0.012	0.007	0.003	0.024	0.043	0.014	0.005	0	0.019	0.031
G	0.006	0.019	0.071	0.007	0.004	0.012	0.009	0.149	0.035	0.036	0.018	0.01	0.011	0.002	0.012
H	0.013	0.016	0.085	0.027	0.006	0.006	0.028	0.003	0.001	0.118	0.108	0.007	0.025	0.061	0.008
I	0.003	0.017	0.018	0.049	0.006	0.024	0.028	0.002	0.004	0.021	0.005	0.005	0.01	0.018	0.015
K	0.003	0.046	0.037	0.004	0.006	0.014	0.083	0.001	0.061	0.041	0.01	0.017	0.044	0.013	0.022
L	0.008	0.01	0.001	0.164	0.006	0.007	0.147	0.032	0.006	0.041	0.008	0.144	0.069	0.01	0.008
M	0.002	0.022	0.025	0.001	0.004	0.162	0	0.01	0.02	0.014	0.007	0.017	0.295	0.024	0.04
N	0.006	0.001	0.023	0.005	0.022	0.019	0	0.04	0.037	0.013	0.102	0.021	0.035	0.015	0.017
P	0.01	0.017	0.107	0.014	0.01	0.011	0.014	0.009	0.028	0.002	0.009	0.012	0.065	0.019	0.015
Q	0.005	0.004	0.01	0.008	0.001	0.015	0.002	0.03	0.139	0.005	0.008	0.008	0.001	0.007	0.179
R	0.007	0.014	0.039	0.003	0	0.046	0.049	0.003	0.028	0	0.069	0.004	0.021	0.16	0.056
S	0.003	0.007	0.006	0.008	0.014	0.001	0.019	0	0.008	0.012	0.001	0.157	0.018	0.054	0.036
T	0.003	0.012	0.008	0.002	0.184	0.017	0.044	0.028	0.007	0.028	0.026	0.091	0.058	0.027	0.045
V	0.012	0.049	0.003	0.04	0.036	0.014	0.006	0.021	0.01	0.019	0.183	0.001	0.027	0.261	0.02
W	0.002	0.241	0.095	0.004	0.037	0.006	0.014	0.018	0.203	0.051	0.004	0.018	0.088	0.001	0.006
Y	0.005	0.005	0.01	0.001	0.027	0.008	0.033	0.011	0.019	0.039	0.041	0.087	0.003	0.001	0.003
Sequence Length is 30															
		$\lvert\widehat{\boldsymbol{\Theta}}_j - \boldsymbol{\Theta}_j\rvert$									$\lvert\widehat{\widetilde{\boldsymbol{\Theta}}}_j - \widetilde{\boldsymbol{\Theta}}_j\rvert$				
Letter	$\lvert\widehat{\boldsymbol{\theta}_0} - \boldsymbol{\theta}_0\rvert$	$j=1$	$j=2$	$j=3$	$j=4$	$j=5$	$j=6$	$j=7$	$j=8$	$j=9$	$j=1$	$j=2$	$j=3$	$j=4$	$j=5$
A	0.001	0.056	0.045	0.128	0	0.003	0.005	0.006	0.043	0.018	0.047	0.051	0.026	0.006	0.019
C	0.012	0.05	0.009	0.037	0.001	0.009	0.012	0.014	0.014	0.002	0.005	0.041	0.006	0.003	0.01
D	0	0.007	0.012	0.035	0.032	0.002	0.053	0.02	0.33	0.033	0.015	0.006	0.006	0.019	0.003
E	0.001	0.01	0.016	0.009	0.006	0.007	0.005	0.008	0.031	0.023	0.022	0.122	0.002	0.025	0.017
F	0.003	0.037	0.014	0.015	0.006	0.002	0.005	0.005	0.01	0.136	0.014	0.006	0.109	0.02	0.008
G	0.002	0.006	0.005	0.27	0.006	0.045	0.088	0.012	0.026	0.007	0.013	0.003	0.01	0.006	0.037
H	0.01	0.008	0.052	0.006	0.054	0.14	0.037	0.004	0.076	0.026	0.019	0.063	0.07	0.015	0.049
I	0.005	0.007	0.038	0.009	0.021	0.004	0.008	0.052	0.02	0.018	0.013	0.013	0.005	0.12	0.002
K	0.004	0.053	0.078	0.074	0.005	0.398	0.007	0.008	0.014	0.01	0.018	0.018	0.009	0.503	0.021
L	0.001	0.016	0.029	0.01	0.005	0.021	0.035	0.019	0.017	0.007	0.001	0.147	0.008	0.04	0.018
M	0	0.022	0.079	0.006	0.017	0.005	0.002	0.01	0.001	0.017	0.031	0.012	0.317	0.019	0.004
N	0.003	0.02	0.002	0.03	0.006	0.04	0.014	0	0.004	0.015	0.437	0.166	0.004	0.04	0.028
P	0.001	0.011	0.17	0.001	0.011	0.033	0.006	0.017	0.002	0.049	0.103	0.021	0.042	0.021	0.018
Q	0.001	0.035	0.004	0.022	0.003	0.014	0.006	0.22	0.03	0.011	0.048	0.011	0.021	0.112	0.271
R	0.001	0.046	0.004	0.024	0.03	0.028	0.001	0.051	0.028	0.002	0.006	0.051	0.05	0.025	0.015
S	0.001	0.191	0.003	0.008	0.001	0.028	0.11	0.132	0.004	0.005	0.026	0.003	0.004	0.008	0.034
T	0.002	0.013	0.01	0.014	0.278	0.002	0.115	0.06	0.008	0.016	0.006	0.006	0.015	0.008	0.009
V	0.003	0.052	0.092	0.085	0.034	0.005	0.012	0.014	0.005	0.296	0.005	0.025	0.006	0.036	0.006
W	0.01	0.024	0.007	0	0.042	0.024	0.041	0.004	0.013	0.016	0.042	0.042	0.016	0.029	0.009
Y	0.005	0.01	0.009	0.012	0.008	0.014	0.005	0.048	0.014	0.013	0.002	0.014	0.045	0.013	0.005

Table 11: Number of Sequences is 500

Sequence Length is 10															
		$\lvert\widehat{\boldsymbol{\Theta}}_j - \boldsymbol{\Theta}_j\rvert$									$\lvert\widehat{\widetilde{\boldsymbol{\Theta}}}_j - \widetilde{\boldsymbol{\Theta}}_j\rvert$				
Letter	$\lvert\widehat{\boldsymbol{\theta}_0} - \boldsymbol{\theta}_0\rvert$	$j=1$	$j=2$	$j=3$	$j=4$	$j=5$	$j=6$	$j=7$	$j=8$	$j=9$	$j=1$	$j=2$	$j=3$	$j=4$	$j=5$
A	0.005	0.007	0.012	0.04	0.016	0.065	0.006	0.002	0.076	0.005	0.005	0.001	0.018	0.001	0.001
C	0	0.006	0.008	0.001	0.008	0.046	0.014	0.003	0.001	0.002	0.005	0.001	0.001	0.003	0.001
D	0.009	0.001	0.007	0.005	0.011	0.002	0.004	0	0.005	0.004	0.016	0.006	0.015	0.012	0.011
E	0	0.003	0.001	0.014	0.015	0.004	0.002	0.004	0.03	0.005	0.097	0.001	0.004	0.006	0.002
F	0.009	0.003	0.001	0.003	0.018	0.063	0.003	0.001	0.004	0.009	0.013	0.014	0.006	0.008	0.002
G	0.012	0.006	0.003	0	0.006	0.003	0.005	0.047	0.032	0	0.001	0.009	0.006	0.078	0.001
H	0.004	0.002	0.01	0.012	0.004	0.008	0.021	0.006	0.002	0	0.007	0.008	0.078	0.004	0.012
I	0.008	0.001	0.008	0.002	0.07	0.082	0.015	0.011	0.001	0.023	0.009	0.006	0.009	0.003	0.002
K	0.011	0.002	0	0.003	0.027	0.005	0.018	0.02	0.021	0.004	0.002	0.004	0.023	0.017	0
L	0.005	0.005	0.01	0.008	0.001	0.004	0.019	0.004	0.067	0.009	0.014	0	0.019	0.019	0.011
M	0	0.008	0.009	0.021	0.01	0.01	0.011	0.034	0.107	0.002	0.002	0.001	0.006	0	0.012
N	0.001	0.003	0.015	0.002	0.012	0.029	0.004	0.001	0.009	0	0.007	0.011	0.003	0.003	0.006
P	0.005	0.019	0.002	0.008	0.003	0.014	0.007	0.012	0.008	0.007	0.002	0.002	0.001	0.007	0.011
Q	0.002	0.006	0.005	0.01	0.001	0.011	0.004	0	0.001	0.001	0.002	0.011	0.002	0.008	0.008
R	0.009	0	0.012	0.007	0.004	0.024	0.009	0.002	0.005	0.011	0.008	0.02	0.002	0.004	0.001
S	0	0.082	0.046	0.01	0.005	0.061	0.034	0.09	0.014	0.001	0.008	0.01	0.007	0.004	0.046
T	0	0.005	0.007	0.002	0.006	0.045	0.158	0.018	0.092	0.01	0.005	0.056	0.009	0.02	0.012
V	0.016	0.005	0.001	0.017	0.029	0.011	0	0	0.021	0.098	0.011	0.004	0.001	0.004	0.009
W	0	0.008	0.002	0.004	0.003	0.006	0.012	0.011	0.035	0.001	0.005	0.001	0.016	0.011	0.007
Y	0.003	0.012	0.001	0.063	0	0.003	0.004	0.008	0.003	0.024	0.012	0.005	0.004	0.001	0.06
Sequence Length is 20															
		$\lvert\widehat{\boldsymbol{\Theta}}_j - \boldsymbol{\Theta}_j\rvert$									$\lvert\widehat{\widetilde{\boldsymbol{\Theta}}}_j - \widetilde{\boldsymbol{\Theta}}_j\rvert$				
Letter	$\lvert\widehat{\boldsymbol{\theta}_0} - \boldsymbol{\theta}_0\rvert$	$j=1$	$j=2$	$j=3$	$j=4$	$j=5$	$j=6$	$j=7$	$j=8$	$j=9$	$j=1$	$j=2$	$j=3$	$j=4$	$j=5$
A	0	0.015	0.003	0.027	0	0.035	0.004	0.022	0.005	0.024	0.01	0.034	0.006	0.065	0.002
C	0.003	0.007	0.005	0.002	0.073	0.002	0.002	0.03	0.003	0.124	0.002	0.004	0.002	0.006	0.001
D	0.005	0.016	0.009	0.006	0.017	0.004	0.005	0.009	0.001	0.004	0.003	0.007	0.001	0.001	0.013
E	0.008	0.023	0.012	0.07	0.008	0.009	0.005	0.016	0.008	0.012	0.004	0.023	0.008	0	0.003
F	0.002	0.001	0	0.027	0.032	0.051	0.005	0.038	0.002	0.01	0.015	0.018	0.002	0.006	0.003
G	0	0.006	0.078	0.008	0.004	0.013	0.002	0.144	0.001	0.014	0.128	0.009	0.008	0.005	0.003
H	0	0.007	0.01	0.006	0.006	0.015	0.005	0.005	0.004	0.003	0	0.017	0.002	0.001	0.004
I	0.003	0.032	0.008	0.023	0.004	0.059	0.047	0.004	0.014	0.008	0.003	0.002	0.004	0.042	0.004
K	0.002	0.008	0.001	0.004	0.007	0.002	0	0.002	0.003	0.017	0.011	0.001	0.005	0.007	0.002
L	0.002	0.177	0.009	0.001	0.011	0.002	0.006	0.012	0.001	0.004	0.001	0.002	0.001	0.001	0.005
M	0.002	0.01	0.01	0.002	0.056	0.08	0	0	0.003	0.002	0.01	0.001	0.093	0.018	0.023
N	0.003	0.019	0.005	0.002	0.005	0.004	0.08	0.035	0.007	0.012	0.007	0.004	0.003	0.002	0.008
P	0.001	0.008	0.02	0.001	0.023	0.001	0.002	0.002	0.004	0.001	0.007	0.002	0.008	0.017	0.13
Q	0	0.004	0.025	0.008	0.063	0.001	0.009	0.003	0.012	0.004	0.009	0.004	0.003	0	0.012
R	0	0.004	0.009	0.014	0.005	0.021	0	0.012	0.064	0.002	0.002	0.017	0.005	0.014	0.009
S	0.005	0.01	0.017	0.021	0.018	0.01	0.012	0.012	0.028	0.002	0.012	0.004	0.014	0	0.003
T	0	0.003	0.001	0.005	0.003	0.012	0.032	0.004	0.003	0.007	0.001	0.165	0.001	0.005	0.026
V	0.001	0.007	0.001	0.002	0.007	0.025	0.001	0.001	0.003	0.002	0.023	0.005	0.01	0.004	0.008
W	0.009	0.006	0.002	0.048	0.011	0.009	0	0.006	0.029	0.009	0.009	0.006	0.004	0.012	0.005
Y	0.001	0.003	0.006	0.006	0.007	0.006	0.006	0.002	0.01	0.008	0	0.004	0.01	0.007	0.003
Sequence Length is 30															
		$\lvert\widehat{\boldsymbol{\Theta}}_j - \boldsymbol{\Theta}_j\rvert$									$\lvert\widehat{\widetilde{\boldsymbol{\Theta}}}_j - \widetilde{\boldsymbol{\Theta}}_j\rvert$				
Letter	$\lvert\widehat{\boldsymbol{\theta}_0} - \boldsymbol{\theta}_0\rvert$	$j=1$	$j=2$	$j=3$	$j=4$	$j=5$	$j=6$	$j=7$	$j=8$	$j=9$	$j=1$	$j=2$	$j=3$	$j=4$	$j=5$
A	0	0.004	0.028	0.003	0.001	0	0.001	0	0.008	0.005	0.002	0.001	0.001	0.004	0.01
C	0.002	0.001	0.011	0.005	0.001	0.001	0.001	0.006	0	0.006	0.002	0.05	0.004	0.003	0.023
D	0.003	0.015	0.004	0.013	0	0.009	0.005	0.007	0.003	0.052	0.03	0.012	0.038	0.006	0.003
E	0	0.019	0.008	0.006	0.123	0.012	0.003	0.018	0.012	0.019	0.004	0.01	0.133	0.003	0.002
F	0.002	0.001	0.015	0.001	0.001	0.007	0.001	0	0.003	0.011	0.015	0	0.005	0	0.027
G	0.002	0.013	0.003	0.001	0	0.007	0.003	0.002	0.005	0.008	0	0.031	0.001	0.005	0.002
H	0.001	0.002	0.007	0.003	0.003	0	0.098	0.031	0	0.143	0.008	0.005	0.002	0.009	0.008
I	0.003	0.005	0.021	0.001	0.006	0.005	0.001	0.007	0.005	0.001	0.004	0.002	0.008	0.019	0.021
K	0.004	0.019	0.027	0.003	0	0.007	0.037	0	0.001	0.009	0.011	0.004	0.006	0.024	0.022
L	0	0.005	0.002	0.135	0.018	0.012	0.022	0.004	0.002	0.004	0.002	0.003	0.008	0.003	0.049
M	0.001	0.008	0.17	0.083	0.009	0	0.022	0.103	0.06	0.001	0.005	0.021	0.013	0.004	0.003
N	0.002	0.006	0.006	0.005	0.001	0.004	0.03	0.004	0.01	0.02	0.008	0.152	0.011	0.003	0.005
P	0.001	0.044	0.056	0.072	0.029	0.012	0.007	0.008	0.001	0.008	0.035	0.001	0.007	0.1	0.243
Q	0.001	0.002	0	0.033	0.02	0	0.004	0.001	0.002	0.017	0.018	0.008	0.004	0.01	0.002
R	0	0.169	0.015	0.021	0.005	0.01	0.001	0	0.004	0.012	0.005	0.013	0.002	0.02	0.018
S	0	0.016	0.007	0.003	0.001	0.002	0.016	0.002	0.003	0.001	0.018	0	0.002	0.001	0.004
T	0	0.006	0.003	0.024	0.002	0.006	0	0.004	0.037	0.001	0.001	0.009	0.008	0.053	0.001
V	0.001	0.002	0.028	0.001	0.01	0.003	0.012	0.003	0.003	0.023	0	0	0.068	0.063	0.024
W	0.006	0.013	0.005	0.013	0.002	0.042	0.004	0.008	0.004	0.022	0.014	0.011	0.009	0.008	0.007
Y	0.001	0.003	0.001	0.003	0.017	0.002	0.005	0.001	0.133	0.065	0.001	0.015	0.011	0.006	0.02

Table 12: Number of Sequences is 1000

Sequence Length is 10															
		$\lvert\widehat{\boldsymbol{\Theta}}_j - \boldsymbol{\Theta}_j\rvert$									$\lvert\widehat{\widetilde{\boldsymbol{\Theta}}}_j - \widetilde{\boldsymbol{\Theta}}_j\rvert$				
Letter	$\lvert\widehat{\boldsymbol{\theta}_0} - \boldsymbol{\theta}_0\rvert$	$j=1$	$j=2$	$j=3$	$j=4$	$j=5$	$j=6$	$j=7$	$j=8$	$j=9$	$j=1$	$j=2$	$j=3$	$j=4$	$j=5$
A	0.002	0.003	0.003	0.012	0.007	0.009	0.009	0	0.004	0.002	0.021	0.002	0.003	0.001	0.011
C	0.005	0.013	0.001	0.001	0.002	0.005	0.003	0	0.004	0.007	0	0.002	0.012	0.003	0.004
D	0.004	0.006	0	0.002	0	0.006	0	0.001	0.005	0.001	0.001	0.003	0.006	0	0
E	0.003	0.132	0.041	0.04	0.001	0.026	0	0.058	0.009	0.002	0.012	0.003	0	0.002	0.017
F	0.001	0.049	0.001	0.001	0.002	0.007	0	0	0.003	0.04	0.004	0.002	0	0.012	0.004
G	0.001	0.001	0.001	0.006	0	0.016	0.001	0.002	0.004	0.002	0.001	0.006	0.001	0.004	0.001
H	0.003	0.004	0.006	0.001	0.007	0.016	0.009	0.055	0.005	0.006	0.01	0.002	0.028	0.003	0.003
I	0	0.032	0.018	0.016	0.064	0.002	0.006	0.014	0.131	0.027	0.024	0.004	0.009	0.002	0.002
K	0.002	0.002	0.003	0.003	0.005	0.015	0.01	0.01	0.006	0	0	0	0	0.005	0
L	0.001	0.001	0.001	0.003	0.005	0.017	0.012	0.001	0.002	0.001	0.001	0.004	0.001	0.009	0.013
M	0.003	0.013	0.001	0.011	0.029	0.017	0.001	0.005	0.008	0	0	0.013	0.005	0.003	0.008
N	0.001	0.001	0.009	0.006	0.001	0.001	0.004	0.003	0.002	0.011	0.017	0.003	0.008	0.01	0.001
P	0.008	0	0.02	0.009	0.005	0.011	0.002	0.003	0.001	0.002	0	0.004	0.044	0.001	0.008
Q	0.007	0.007	0.044	0.039	0.003	0.012	0.01	0	0.002	0.008	0.021	0.004	0.003	0.007	0.004
R	0.011	0.003	0.005	0.002	0.007	0.092	0.013	0.002	0.009	0.009	0.006	0.003	0.004	0.006	0
S	0.001	0.02	0.08	0.016	0.003	0.002	0.002	0.002	0.011	0.002	0.012	0.005	0	0.004	0.058
T	0.003	0.016	0.009	0.005	0.008	0.014	0.018	0.003	0.005	0.031	0.008	0.01	0.002	0.004	0.027
V	0.009	0.004	0.003	0.003	0.016	0.004	0.009	0.076	0.042	0.002	0.002	0.078	0.006	0.085	0.006
W	0.001	0.007	0.001	0.004	0.006	0.005	0.093	0.026	0.003	0.011	0.008	0.008	0.07	0.008	0.002
Y	0.003	0.014	0.001	0.017	0.014	0.008	0.002	0.005	0.023	0.005	0.003	0.004	0.002	0.002	0.002

Sequence Length is 20															
		$\lvert\widehat{\boldsymbol{\Theta}}_j - \boldsymbol{\Theta}_j\rvert$									$\lvert\widehat{\widetilde{\boldsymbol{\Theta}}}_j - \widetilde{\boldsymbol{\Theta}}_j\rvert$				
Letter	$\lvert\widehat{\boldsymbol{\theta}_0} - \boldsymbol{\theta}_0\rvert$	$j=1$	$j=2$	$j=3$	$j=4$	$j=5$	$j=6$	$j=7$	$j=8$	$j=9$	$j=1$	$j=2$	$j=3$	$j=4$	$j=5$
A	0.004	0.004	0.01	0.002	0.007	0.001	0.002	0.001	0.051	0.02	0.009	0.003	0.011	0	0.008
C	0	0.004	0.002	0.007	0.002	0.002	0.004	0.027	0.003	0.007	0.003	0.002	0.001	0.002	0.003
D	0.001	0.001	0.009	0.006	0	0.121	0.006	0.01	0.013	0.01	0	0.001	0.002	0.003	0.008
E	0	0.005	0.019	0.012	0.002	0.011	0.003	0.001	0.007	0.031	0.02	0.001	0.004	0.056	0.014
F	0	0.004	0.004	0.002	0.006	0.003	0.012	0.017	0.012	0.001	0.001	0	0.048	0.006	0.004
G	0	0.016	0.002	0	0.001	0.006	0.059	0.006	0.011	0.016	0.001	0.002	0.001	0	0.002
H	0	0.003	0	0.072	0.005	0.016	0.003	0.004	0.009	0.011	0	0.023	0.003	0.003	0.001
I	0.002	0.016	0.004	0.003	0.004	0.006	0	0.01	0.026	0.068	0.003	0.01	0.003	0.012	0.006
K	0	0.05	0.005	0	0.083	0.011	0.01	0.005	0.003	0.012	0.001	0.005	0	0	0.002
L	0.002	0.024	0.006	0.001	0.005	0.01	0	0.001	0.037	0.001	0.007	0.001	0	0.001	0.017
M	0	0.005	0.002	0.013	0.005	0.097	0.004	0.015	0.011	0.001	0.012	0.003	0.003	0.019	0.006
N	0.001	0.032	0.001	0.006	0.006	0.003	0.002	0.001	0.006	0.034	0.028	0.001	0.003	0.002	0.001
P	0.001	0.066	0.005	0.002	0.003	0.008	0.002	0.013	0.003	0.003	0.003	0.099	0.005	0	0.005
Q	0.005	0.031	0.006	0.02	0	0.005	0.011	0	0.011	0.023	0.01	0.003	0	0.013	0.023
R	0.001	0.012	0.008	0.001	0.002	0	0.012	0.007	0	0.006	0.008	0.005	0.001	0.003	0.001
S	0.002	0.024	0.003	0.01	0.004	0.014	0.002	0.002	0.011	0.001	0.003	0.001	0.003	0.011	0.005
T	0.001	0.022	0.061	0.003	0.04	0.007	0.006	0.002	0.002	0.005	0.003	0.016	0.052	0.002	0.019
V	0.004	0.013	0.012	0	0.006	0.057	0.023	0.012	0.004	0.008	0	0.007	0	0.003	0.002
W	0.001	0	0.002	0.002	0.004	0	0.005	0.012	0.026	0.003	0.063	0.007	0.047	0.004	0.001
Y	0.002	0.008	0.008	0.002	0.004	0.002	0.003	0.005	0.002	0.001	0.022	0.01	0.002	0.01	0.091

Sequence Length is 30															
		$\lvert\widehat{\boldsymbol{\Theta}}_j - \boldsymbol{\Theta}_j\rvert$									$\lvert\widehat{\widetilde{\boldsymbol{\Theta}}}_j - \widetilde{\boldsymbol{\Theta}}_j\rvert$				
Letter	$\lvert\widehat{\boldsymbol{\theta}_0} - \boldsymbol{\theta}_0\rvert$	$j=1$	$j=2$	$j=3$	$j=4$	$j=5$	$j=6$	$j=7$	$j=8$	$j=9$	$j=1$	$j=2$	$j=3$	$j=4$	$j=5$
A	0.001	0.003	0.038	0.007	0.025	0.011	0.001	0.005	0.005	0.001	0.043	0.01	0.008	0.05	0.007
C	0.003	0	0.091	0.039	0.009	0	0.003	0.003	0.002	0	0.017	0.007	0.006	0.006	0
D	0	0.03	0.005	0.026	0.004	0.025	0.002	0.006	0.079	0.009	0.002	0.004	0.012	0.002	0.015
E	0.003	0.005	0.005	0.002	0.001	0.007	0.003	0	0.003	0.025	0.005	0.012	0.001	0.002	0.002
F	0	0	0.004	0.008	0	0	0.019	0	0.002	0.025	0.001	0.003	0	0.001	0.001
G	0.001	0.004	0.002	0.026	0.003	0.001	0.001	0.002	0.004	0.002	0.002	0.022	0.008	0.016	0.016
H	0	0.006	0.008	0.002	0	0.001	0.002	0	0.004	0.001	0.001	0.012	0.006	0.002	0.01
I	0.001	0.001	0.002	0.028	0.017	0	0	0.004	0.01	0.008	0.005	0.006	0.013	0.011	0.003
K	0.002	0.009	0.002	0.002	0.002	0	0.009	0.002	0.003	0.001	0.01	0.009	0.007	0.007	0
L	0.001	0.003	0.016	0.009	0.04	0.015	0.001	0.002	0	0.015	0.004	0.005	0.001	0.095	0.006
M	0.003	0.017	0	0.017	0.003	0.002	0.002	0.003	0.003	0.001	0.025	0.001	0.005	0.001	0.123
N	0	0.009	0.001	0.003	0.002	0	0.011	0.004	0.007	0.002	0.022	0.08	0	0.019	0.012
P	0.001	0.09	0.01	0	0.006	0.001	0.036	0.014	0.006	0.064	0	0.067	0.001	0.004	0.024
Q	0.001	0	0.001	0	0.01	0.001	0.003	0	0.001	0.002	0.005	0.008	0	0.001	0.001
R	0	0.014	0.003	0.007	0.009	0.001	0.004	0.002	0.001	0.003	0	0.002	0.004	0.003	0.039
S	0.001	0.003	0.002	0.003	0.025	0.003	0.001	0.001	0.01	0.007	0.003	0.001	0.01	0.005	0.002
T	0.002	0.025	0.009	0.007	0	0.044	0.037	0.003	0.002	0.006	0.001	0.009	0.001	0.009	0.001
V	0.003	0.007	0.007	0.003	0.006	0.009	0.01	0.005	0.013	0.011	0.006	0.01	0.086	0.001	0.001
W	0.001	0.002	0.007	0.007	0.015	0.002	0.031	0.054	0.006	0.008	0.001	0.02	0.001	0.002	0.003
Y	0.001	0	0.03	0	0.011	0.002	0.004	0.002	0.009	0.002	0.002	0.049	0.004	0	0.006

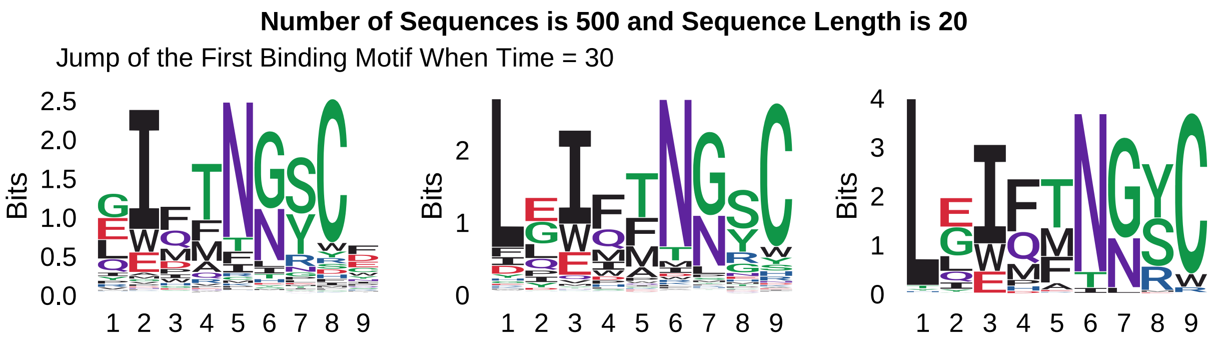

Figure 14: Jump for the case of the number of sequences is 500 and sequence length is 20.

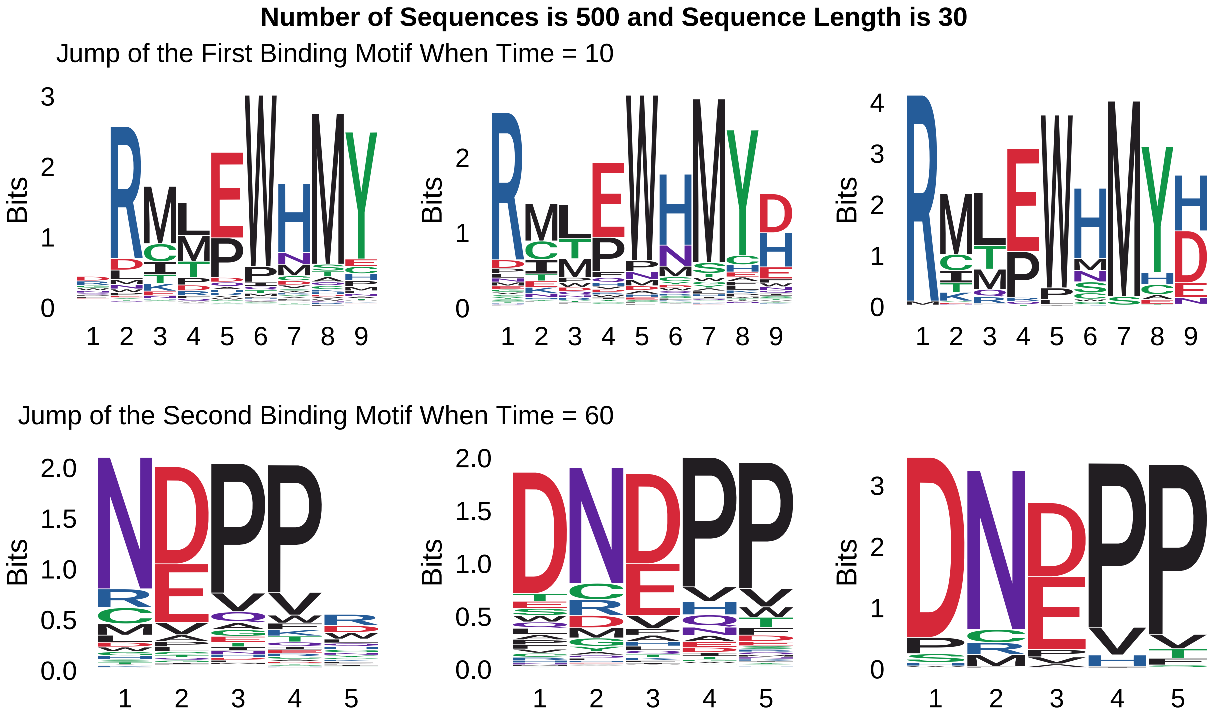

Figure 15: Jump for the case of the number of sequences is 500 and sequence length is 30.
